# Unmasking the common enemy: drug resistance mechanisms across three different EGFR inhibitor generations are associated with co-targetable alterations in extracellular matrix signaling

**DOI:** 10.1186/s12964-026-02927-8

**Published:** 2026-05-06

**Authors:** Yu Zhang, Okan Gültekin, Rudolf Kupčík, Youssif Budagaga, Dimitrios Vagiannis, Ziba Sabet, Kaisa Lehti, Jakub Hofman

**Affiliations:** 1 Department of Pharmacology and Toxicology, Faculty of Pharmacy in Hradec Králové, Charles University, Akademika Heyrovského 1203, Hradec Králové, 500 05 Czech Republic; 2 Department of Microbiology, Tumor and Cell Biology, Karolinska Institutet, Solnavägen 9, Solna, 171 65 Sweden; 3 Department of Oncology-Pathology, Karolinska Institutet, Anna Steckséns gata 30A, D2:04, Solna, 171 64 Sweden; 4 Biomedical Research Centre, University Hospital Hradec Králové, Sokolská 581, Hradec Králové, 500 05 Czech Republic; 5 Department of Biomedical Laboratory Science, Norwegian University of Science and Technology, Erling Skjalgssons gate 1, Trondheim, 7491 Norway

**Keywords:** Non-small cell lung cancer, Epidermal growth factor receptor tyrosine kinase inhibitor, Acquired drug resistance, Drug combination, Extracellular matrix

## Abstract

**Background:**

Epidermal growth factor receptor-tyrosine kinase inhibitors (EGFR-TKIs) have transformed non-small cell lung cancer (NSCLC) treatment, offering substantial survival benefits. However, acquired resistance remains a significant obstacle, undermining long-term efficacy. While specific mechanisms of EGFR-TKI resistance have been reported, potential shared mechanisms across EGFR-TKI generations have remained unclear.

**Methods:**

Drug-resistant HCC827 and NCI-H1975 cells were developed by a 10-month stepwise selection using gefitinib, dacomitinib, and osimertinib, representing first, second, and third EGFR-TKI generations, respectively. Global proteomes of the resistant and parental NSCLC cell lines were compared using liquid chromatography tandem mass spectrometry. After bioinformatic pathway analyses, the candidate protein and gene alterations were validated by western blotting and droplet digital PCR. Drug (cross-)resistance patterns and reversal responses were quantified by MTT assay after single and combination drug treatments, or gene silencing by small interfering RNA. Invasive cell activities were evaluated in spheroid models within three-dimensional (3D) collagen matrices. Bioinformatics analyses of open-access transcriptomic datasets were applied to explore gene expression alterations linked to clinical EGFR mutations and disease prognosis and relapse.

**Results:**

We established EGFR-TKI-resistant NSCLC cell lines for all three drug generations, demonstrating cross-resistance and significantly enhanced invasive potential in 3D collagen. Notably, extracellular matrix components and signaling proteins (FN1, FAK, YAP1) were found altered and validated as synergistically targetable resistance drivers across all three drug generations, irrespective of cellular background. Moreover, the second- and third-generation models shared co-targetable dysregulations in cancer stemness regulators (Hedgehog, Notch), anti-apoptotic protein BCL-2, and the drug efflux transporter ABCG2. Finally, transcriptomic analysis showed that in human EGFR-positive NSCLC tumors, overexpression of FN1 and collagen-related genes was associated with post-treatment relapse and poor patient survival.

**Conclusions:**

This study identifies/validates key mechanisms of EGFR-TKI resistance and suggests combinatorial therapeutic strategies as potential interventions against EGFR-TKI-resistant lung cancers, with promising implications for improving clinical outcomes.

**Supplementary Information:**

The online version contains supplementary material available at 10.1186/s12964-026-02927-8.

## Abstract (plain language summary)

Drugs that block the epidermal growth factor receptor (EGFR) have greatly improved the treatment of a common form of lung cancer called non-small cell lung cancer (NSCLC). Many patients live longer thanks to these medicines. However, after some time, the cancer cells often stop responding to the treatment and begin to grow again. This is known as “drug resistance.” In our study, we grew lung cancer cells in the laboratory and exposed them for several months to three generations of EGFR-targeting drugs. Over time, the cells became resistant not only to the drug they were exposed to, but also to other drugs of the same group. We then compared these resistant cells with their original, drug-sensitive versions to find out what had changed inside them. We discovered that the resistant cancer cells had altered the structure and composition of their surroundings — the so-called extracellular matrix — and had become more mobile and invasive. These changes were linked to increased levels of several key proteins that help cancer cells survive, move, and avoid being killed by treatment. When we blocked these proteins or their signaling pathways, the resistant cells became sensitive to EGFR-targeting drugs again. Our findings suggest that drug resistance in lung cancer involves shared biological changes across different treatments. Combining EGFR-targeting drugs with medicines that block these resistance-related pathways could make future therapies more effective and help patients live longer.

**Supplementary Information:**

The online version contains supplementary material available at 10.1186/s12964-026-02927-8.

## Background

Lung cancer remains the leading cause of cancer mortality worldwide, being responsible for approximately 18% of all cancer-related deaths [1]. Among lung cancer cases, around 80–85% are classified as non-small cell lung cancer (NSCLC), the most common clinical subtype [2]. Traditional chemotherapy has long been used to treat NSCLC; however, its limited efficacy and significant side effects often constrain substantial clinical benefit for many patients [3]. In recent years, therapeutic strategies have increasingly pivoted toward targeted treatments with enhanced safety profiles. Notably, over 60% of NSCLC patients exhibit epidermal growth factor receptor (EGFR) overexpression or mutation, positioning EGFR as a key therapeutic target [4]. Since 2003, EGFR-tyrosine kinase inhibitors (EGFR-TKIs) have marked a significant advancement in NSCLC treatment, demonstrating marked improvements in progression-free survival (PFS) and overall survival (OS) over standard chemotherapy. However, despite initial responses, nearly all patients eventually develop resistance to EGFR-TKI therapy, with a median PFS of 9.2–18.9 months, highlighting the ongoing challenge of therapeutic resistance [5–8].

Three generations of EGFR-TKIs have been developed to address these resistance mechanisms in NSCLC. In the early 2000s, the FDA approved gefitinib and erlotinib, both first-generation EGFR-TKIs, which selectively bind to the ATP-binding site of wild-type EGFR and common activating mutations (exon 19 deletions and L858R) [9–12]. However, resistance developed rapidly, with limited survival benefits, leading to restricted clinical use and eventual regulatory withdrawal [13, 14]. Mechanistic studies identified the EGFR T790M mutation in exon 20 in approximately 50–60% of resistant cases, which spurred the development of second-generation EGFR-TKIs, including dacomitinib and afatinib, which irreversibly inhibit EGFR and other members of the human epidermal growth factor receptor family, including T790M mutants [15–17]. Yet, response rates remained suboptimal, and disease recurrence continued to pose a significant challenge [18]. Third-generation EGFR-TKIs, such as osimertinib and olmutinib, were subsequently introduced to overcome T790M-mediated resistance through selective and irreversible inhibition of this mutation, achieving notable PFS and OS improvements. Nevertheless, acquired resistance still remains an obstacle to sustained therapeutic efficacy [19].

Resistance mechanisms to EGFR-TKIs in NSCLC can be classified as EGFR-dependent or EGFR-independent [20]. EGFR-dependent resistance is often driven by additional target mutations (e.g., T790M, C797S) or gene amplification [5, 18]. In addition to genetic alterations, activation of resistance-related signaling pathways and their interactions with tumor microenvironment (TME) significantly contributes to EGFR-TKI resistance. Such interactions are mainly mediated by extracellular matrix (ECM), a complex network of proteins like collagen and fibronectin, which shapes the TME and provides structural and biochemical cues that support NSCLC growth, metastasis, and drug resistance [21]. Although some specific EGFR-independent mechanisms have been previously described [22, 23], current knowledge is substantially fragmented and verification of their real impact for tumor defense is still missing. In addition, no single study has investigated possible shared resistance mechanisms, which might be universal across the EGFR-TKI’s generations. Such knowledge could be a valuable platform for the design of effective resistance-reversal strategies and prediction of resistance patterns in the future generations of EGFR-TKIs.

In our study, we generated NSCLC cell lines resistant to each generation of EGFR-TKIs (first-generation gefitinib, second-generation dacomitinib, and third generation osimertinib) and conducted investigations into potentially shared resistance mechanisms and cross-resistance patterns. We focused on characterizing invasive and metastatic potentials regulated by ECM-related signaling, which may play a central role in acquired resistance. Importantly, unlike conventional transcriptomic or proteomic analyses [24], we validated key candidate pathways through resistance-reversal assays, confirming their role in EGFR-TKI resistance. These experiments provided a solid foundation for designing pharmacotherapeutic strategies that may be beneficial to oncology patients experiencing EGFR-TKI resistance. Finally, we performed complex bioinformatics analyses to compare our findings with existing studies and evaluate the implications of the tested pathways in lung tumorigenesis and EGFR mutation-associated NSCLC relapse.

## Methods

### Chemicals and reagents

Afatinib (cat. no. S7810), dacomitinib (cat. no. S2727), erlotinib (cat. no. S7786), gefitinib hydrochloride (cat. no. S5098), olmutinib (cat. no. S8294), osimertinib (cat. no. S7297) and sonidegib (cat. no. S2151) were from Selleckchem (Houston, TX, USA). RO4929097 (cat. no. HY-11102), defactinib (cat. no. HY-12289), verteporfin (cat. no. HY-B0146), Ko143 (cat. no. HY-10010) and venetoclax (cat. no. HY-15531) were from MedChem Express (New Jersey, NJ, USA). Primary antibodies against human protein targets were from Santa Cruz Biotechnology (Dallas, TX, USA), Cell Signaling Technology (Danvers, MA, USA), Abcam (Cambridge, UK) and Abclonal (Woburn, MA, USA), respectively (Table S1). Secondary anti-mouse antibody (cat. no. sc-516102) and anti-rabbit antibody (cat. no. sc-2357) were from Santa Cruz Biotechnology. 10 × Minimum Essential Medium (MEM) and Opti-MEM were from Gibco BRL Life Technologies (Rockville, MD, USA). Lipofectamine™ 3000 transfection reagent was bought from Invitrogen (Waltham, MA, USA). Small interfering RNA targeting human fibronectin (siRNA-FN1; cat. no. 4392420; assay: s223585) and Silencer™ Select Negative Control (siRNA-NC; cat. no. 4390843) were obtained from Ambion (Austin, TX, USA). TRI Reagent was accessed from the Molecular Research Center (Cincinnati, OH, USA). Oligo(dT) was purchased from Generi Biotech (Hradec Králové, Czech Republic). TaqMan probes targeting *EGFR*, *BCL2*, *BAX*, *BAD*, *BIRC5*, *XIAP*, *TRAIL*, *B2M* and *GAPDH* were purchased from Applied Biosystems Life Technologies (Carlsbad, CA, USA). Droplet digital PCR (ddPCR) oils and ddPCR supermix for probes (no dUTP) were obtained from Bio-Rad Laboratories (Hercules, CA, USA). Q5^®^ Hot Start High-Fidelity DNA Polymerase (supplied with 5X Q5^®^ Reaction Buffer and 5X Q5^®^ High GC Enhancer), ProtoScript^®^ II Reverse Transcriptase and Deoxynucleotide (dNTP) Solution Mix (for ddPCR assay) were obtained from New England Biolabs (Ipswich, MA, USA). NucleoSpin Gel and PCR Clean‑up Kit was purchased from Macherey-Nagel (Düren, Germany). Triethylammonium bicarbonate (TEAB), formic acid (FA), acetonitrile (ACN), water for nano-flow circuit of nanoLC system, Pierce™ Trifluoroacetic Acid (TFA) and tris(2-carboxyethyl)phosphine hydrochloride were obtained from Thermo Fisher (Waltham, MA, USA). 4-(2-hydroxyethyl)-1-piperazineethanesulfonic acid (HEPES) solution, sodium pyruvate solution, glucose solution, collagen from rat tail (type I), sodium deoxycholate (SDC), s-methyl methanethiosulfonate, Benzonase^®^ Nuclease, ethyl acetate, protease inhibitor, Roswell Park Memorial Institute medium 1640 (RPMI-1640), 3-(4,5-dimethylthiazol-2-yl)-2,5-diphenyltetrazolium bromide (MTT), dimethyl sulfoxide (DMSO), fetal bovine serum (FBS), 10 mM dNTPs (for PCR) and phosphate buffered saline (PBS) were bought from Sigma Aldrich (St. Louis, MO, USA). ACN for samples processing and for loading line of nanoLC system was from Honeywell (Charlotte, NC, USA).

### Cell lines and generation of drug-resistant cells

The HCC827 (C827/par) and NCI-H1975 (H1975/par) cell lines were purchased from the American Type Culture Collection (Manassas, VA, USA). C827 cells harbor a basic activating *EGFR* exon 19 deletion mutation, while H1975 cells contain both the gatekeeper EGFR-T790M and activating L858R mutations. These cell lines were selected based on the specific affinities of investigated EGFR-TKIs to the mutated *EGFR* forms, as described in the background [25–27].

Gefitinib-resistant C827 (C827/GEF), dacomitinib-resistant H1975 (H1975/DAC), and osimertinib-resistant H1975 (H1975/OSI) cell lines were established in our laboratory using a standard stepwise selection method. The stepwise selection method for generating drug-resistant cell lines involved determining the IC_50_ values of the EGFR-TKIs for the corresponding parental cell lines. These IC_50_ values were then used as the starting concentrations for the first round of drug exposure. After 72-hour treatment, dead cells were removed by washing with PBS, and the surviving cells were cultured in drug-free media to allow recovery. When cell confluence reached 70%, the same concentration of EGFR-TKIs was re-applied for the next round of selection. The IC_50_ values were monitored by MTT assays after every 3 rounds of selection, and the drug concentrations were progressively increased based on the determined IC_50_ values to induce robust and stable resistance. After approximately 10 months of continuous drug treatment, the acquired drug-resistant cell lines were successfully established.

The C827/par and H1975/par cell lines were cultured in RPMI-1640 medium supplemented with 10% FBS, 10 mM HEPES, 1 mM sodium pyruvate, and 15 mM glucose. For drug-resistant lines, C827/GEF, H1975/DAC, and H1975/OSI were maintained in RPMI-1640 medium supplemented with 10 nM gefitinib, 1.5 µM dacomitinib, and 1.5 µM osimertinib, respectively, to sustain the acquired resistance. To prevent any potential interference during experiments, EGFR-TKIs were withdrawn from the media at least 5 days prior to experimentations. All the used cell lines were maintained at a passage number of < 25 and incubated at 37 °C in a 5% CO_2_ atmosphere. *Mycoplasma* contamination was monitored regularly using PCR tests. The solvent DMSO (used to dissolve EGFR-TKIs and other compounds) was kept at concentrations below 0.5% in all experiments, and vehicle controls were included to mitigate any potential effects caused by DMSO.

### Three-dimensional (3D) spheroid cell culture in collagen matrices

The invasive potential of the cell lines was evaluated using a 3D spheroid cell culture assay, with minor modifications to previously described methods [28]. C827/par, H1975/par, and their drug-resistant variants were seeded at a density of 2.5 × 10³ cells per well in ultra-low attachment 96-well plates (PerkinElmer, Waltham, MA, USA) to form spheroids.

To prepare the collagen matrix, collagen from rat tail (type I) was dissolved in 0.05% acetic acid at 4 ˚C to a final concentration of 4.5 mg/mL. The collagen-based hydrogel was then prepared by mixing 2 × MEM and the collagen stock solution in a 1:1 ratio on ice, as previously described [29]. The hydrogel mixture was neutralized using NaOH to a final pH of 7.5-8.

Each spheroid was suspended in 30 µL of collagen-based hydrogel and rapidly embedded onto a 48-well plate. The hydrogel was allowed to form solid crosslinks for 1 h at 37 ˚C. After solidification, 300 µL of culture media, with or without inhibitors (2.5 µM defactinib or 0.05 µM verteporfin), was added to each well.

Images of the cancer spheroids were captured using an inverted microscope (TCM 400; Labo America, Fremont, CA, USA), with image acquisition supported by QuickPHOTO CAMERA software (version 3.2; PROMICRA, Prague, Czech Republic). To assess the proliferative and invasive changes in the spheroids, the QuPath software (version 0.4.2) [30], an open-source image analysis tool, was used to quantify the results.

### MTT cell viability assay

Cell viability was assessed based on the MTT assay as described [31]. Cells were seeded in 96-well plates at the following densities: H1975/par, H1975/DAC, and H1975/OSI at 9.0 × 10³ cells/well; C827/par and C827/GEF at 4.0 × 10⁴ cells/well, except for FN1 knockdown experiments (H1975/par, H1975/DAC, and H1975/OSI at 5.0 × 10³ cells/well) and resistance reversal studies focusing on the Hedgehog and Notch pathways (H1975/par and its drug-resistant variants at 3.0 × 10³ cells/well).

After 24 h of incubation, cells were exposed to serial dilutions of drugs or drug combinations for the durations specified in the figure legends. Following treatment, cells were rinsed with prewarmed 1 × PBS solution, and a freshly prepared MTT solution (1 mg/mL) in Opti-MEM was added for a 1-hour incubation period.

After incubation, the MTT solution was removed, and the resulting formazan crystals were dissolved in DMSO for 10 min at 37 ˚C. Absorbance was measured at 570/690 nm using a microplate reader (Tecan Infinite M200, Männedorf, Switzerland). Background absorbance at 690 nm was subtracted from the data obtained at 570 nm before normalizing the data. Medium containing vehicle or 40% DMSO were defined as 100% or 0% cell viability controls, respectively.

### Resistance reversal

Drug combination studies were conducted in established EGFR-TKI-resistant cell lines. Initially, antiproliferative activities of potential resistance modulators were evaluated to determine optimal concentrations for follow-up reversal studies. Based on the outcomes from the MTT assay, the following modulators were selected for further testing: Ko143 (1 µM) for inhibiting ABCG2; venetoclax (1 µM) for inhibiting BCL-2; sonidegib (5 µM) for Hedgehog pathway inhibition; RO4929097 (10 µM) for Notch pathway inhibition; defactinib (2.5 µM) for FAK inhibition; and verteporfin (0.05 µM) for YAP1 signaling inhibition.

For the drug combination experiments, cells were seeded in 96-well plates at the previously mentioned densities and incubated for 24 h. Following this incubation, drugs were serially diluted in growth media and applied according to specific treatment protocols. The concentrations of modulators, preincubations and exposure times were carefully selected and validated prior to experiments, with consideration for the timing of inhibition, as certain targets, such as ABCG2, are inhibited quickly, while the inhibition of pathways, like Hedgehog and Notch, requires longer treatment periods. In the case of ABCG2/BCL-2 inhibition, cells were pre-incubated with Ko143 or venetoclax for 2 h, followed by the treatment with EGFR-TKIs or the modulators alone, or their combinations, for 48 h. For Hedgehog pathway inhibition, cells were exposed to EGFR-TKIs or sonidegib alone or in combination for 120 h. In Notch pathway inhibition studies, cells were pre-incubated with RO4929097 for 24 h, followed by the treatment with EGFR-TKIs or RO4929097 alone or their combinations for 72 h. Finally, for FAK and YAP1 pathway inhibition, cells were treated with EGFR-TKIs or defactinib/verteporfin alone, or in combination, for 48 h.

After the treatment intervals, the MTT assay was performed, and data were collected using a Tecan Infinite M200 microplate reader as described earlier. The combination effects were quantified using the Chou-Talalay method in CompuSyn 3.0.1 software (ComboSyn Inc.; Paramus, NJ, USA), with combination index (CI) values calculated to characterize the drug interactions as synergistic (< 0.9), additive (0.9–1.1), or antagonistic (> 1.1) [32].

### siRNA transfection for FN1 knockdown

Since no pharmacological inhibitor is available for the FN1 protein, related resistance reversal studies were conducted using siRNA-mediated knockdown. For the FN1 knockdown assay, H1975/par, H1975/DAC, and H1975/OSI cell lines were plated in 96-well plates at a density of 5.0 × 10³ cells/well and incubated for 24 hours. Following this, cells were transfected with Lipofectamine™ 3000 mixed with siRNA-FN1 at a final concentration of 25 nM for 24 hours, according to the supplier’s protocol. To control potential interference from the transfection process, parallel samples were transfected with scrambled siRNA-NC (negative control) at the same concentration. After transfection, dacomitinib or osimertinib were applied to both the drug-resistant and their parental counterparts at varying concentrations. After 48 hours of drug treatment, cell viability was assessed using the MTT assay. The siRNA sequence used for FN1 knockdown was 5’-GGCUCAGCAAAUGGUUCAGtt-3’. Gene knockdown efficiency was confirmed by western blotting, and the results are shown in Fig. S3A-C.

### Global liquid chromatography/tandem mass spectrometry (LC-MS/MS)

Global LC-MS/MS proteomic analysis was performed as an initial screening for resistance-related protein candidates in our EGFR-TKI-resistant cell lines. Cells were washed by cold 1 × PBS solution and lysed in 200 mM TEAB buffer containing 3% SDC. Equivolume pooled samples from three independent preparations were generated for each of the examined cell subline. Benzonase^®^ Nuclease (1 µL/mL) was added, then the cell suspension was centrifuged at 15,000 g at 4 ˚C for 10 min. For protein digestion, the equal aliquots of resulting supernatants were incubated with 5 mM tris(2-carboxyethyl)phosphine hydrochloride at 60 ˚C for 1 h, then with 10 mM s-methyl methanethiosulfonate at room temperature for 10 min. Subsequently, ice-cold acetone (-20 °C) was added into the samples and protein precipitation proceeded at -20 ˚C for 4 h. Then, the mixture was centrifuged at 8000 g for 10 min at 4 ˚C and supernatant was discarded. Dried pellets were re-dissolved in 100 mM TEAB buffer containing 0.1% SDC. Afterwards, Lys-C/Trypsin Mix (Promega; Madison, WI, USA) was added into the samples for an overnight-incubation at 37 ˚C. SDC was removed from the samples through phase-transfer extraction using water-saturated ethyl acetate [33]. Digested and dried protein samples were labelled using a TMTsixplex™ Isobaric Label Reagent Kit (Thermo Fisher, Waltham, MA, USA), according to the protocol introduced by the manufacturer. Equal amounts of samples were pooled together and desalted using Discovery^®^ DSC-18 solid-phase extraction cartridges (Sigma Aldrich; pre-processed with methanol, then with 5% ACN/0.1% TFA solution). Peptides were eluted with 50% ACN/0.1% TFA and evaporated to dryness using vacuum concentrator (Labconco; Kansas City, MO, USA).

For LC-MS/MS analysis, a two-instrument coupled system consisting of UltiMate 3000 RSLCnano system and Q-Exactive Plus mass spectrometer (both Thermo Scientific; Bremen, Germany) was employed. The sample multiplex was re-dissolved in the loading solvent (2% ACN and 0.1% TFA). 1 µg of peptide sample was first captured by the PepMap100 C18 trap column (75 μm × 20 mm, 3 μm), then separated with on PepMap RSLC C18 analytical column (75 μm × 250 mm, 2 μm). A linear segmented gradient, based on the mixture of phase A (0.1% FA in 2% ACN) and phase B (0.1% FA in 80% ACN), was performed at 250 nL/min flow rate for a total 240-min with settings as follows: [1] from 2% to 9% phase B: 57 min; [2] from 9% to 34.5% phase B: 160 min; [3] from 34.5% to 45% phase B: 23 min. Eluting peptides were directly introduced into the Q-Exactive Plus mass spectrometer through Nanospray Flex ion source (Thermo Scientific). Positive ion full scan MS spectra were acquired on Orbitrap within 350–1600 m/z using 3 × 10^6^ automated gain control (AGC) target at 70,000 full width at half maximum (FWHM) resolution with a maximum ion injection time of 100 ms. In MS2 mode, the top 10 precursors with minimal AGC target of 1 × 10^3^ and charge state (≥ 2 and ≤ 8) were fragmented and analyzed at resolution 35,000 FWHM. Isolation window was set to 1.6 m/z and normalized collision energy to 33. MS2 AGC target was 1 × 10^5^ and a maximum injection time of 120 ms. Fixed first mass was set to 100 m/z and dynamic exclusion window was 17 s. Sample multiplex was technically analyzed in triplicate.

For the data processing, MaxQuant software (version 1.6.14.0) and its search engine, Andromeda, was used [34, 35]. UniProtKB Human Reference Proteome database (downloaded on 18/06/2021; 78,139 entries) was employed for comparing the measurements from MS/MS spectra. To processing data from Tandem Mass Tag, the group specific parameter was defined as the reporter ion in MS2. The intensities of the reporter ions were calibrated by the Tandem Mass Tag correction factor offered by the manufacturer. The fixed modification was defined as the alkylation of cysteine sulfhydryl groups to dithiomethane, while the acetylation, methionine and oxidation on protein N-term were considered as the variable modifications. Trypsin/P with maximum 2 missed cleavages was indicated as a protease. Other remaining parameters were kept as their default settings in MaxQuant. The exported files from MaxQuant were processed via Perseus analytical platform (version 1.6.2.1; [36]). Corrected data were transformed into log2 values with a median normalization. Only proteins with at least two technically replicated valid value in each Tandem Mass Tag channel were accepted for further analysis. Data were visualized in scatterplots which were designed in R [37]. The log of sum of normalized protein levels were presented on Y-axis, while the relevant log2 fold changes were shown on X-axis. Two-tailed significance B test (with a 95% significance level) was performed, and for those targets, which passed the test, were highlighted (blue color: ECM-related proteins; red color: other proteins).

### Western blotting

The expression of proteins of interest was detected by western blotting, which was performed with modifications according to protocols reported in our recent papers [31, 38]. Briefly, cells were collected into cell lysis buffer (20 mM Tris, 150 mM NaCl, 12.8 mM ethylenediamine tetraacetic acid (EDTA), 1 mM ethylene glycol-bis(β-aminoethyl ether)-N, N,N′,N′-tetraacetic acid (EGTA), 4.2 mM Na-pyrophosphate, 1 mM Na_3_VO_4_, and 1% Triton X-100) containing 1% protease inhibitor, and then subjected to 30-minute centrifugation at 12,000 g at 4˚C. The total protein concentration was quantified using a Bradford assay reagent. Protein samples, using a total loading amount of 20 µg, were separated by 8% or 10% sodium dodecyl sulfate polyacrylamide gel electrophoresis (SDS-PAGE) gels. After electrophoresis, the separated proteins were transferred to polyvinylidene difluoride (PVDF) membranes using the Trans-Blot Turbo™ Transfer System (Bio-Rad Laboratories, Hercules, CA, USA). Unspecific signals were blocked by incubating the membranes with 1 × Tris-buffered saline with 0.1% Tween 20 detergent (TBST) solution (containing 5% non-fat milk) for 90 min at room temperature. Specific primary antibodies, diluted in 1 × TBST solution as detailed in Table S1, were applied to the membrane for overnight incubation at 4˚C. The following day, membranes were washed three times with 1 × TBST solution. Secondary antibodies, horseradish peroxidase (HRP)-conjugated anti-mouse or anti-rabbit (diluted 1:2000 in 1 × TBST solution), were then applied to the membranes for a 1.5-hour incubation at room temperature. Before the final detection, the membranes were rinsed three more times with 1 × TBST solution. Immobilon Western Chemiluminescent HRP Substrate (EMD Millipore; Billerica, MA, USA) was applied to the membranes, which were then scanned using the Chemi Doc™ MP Imaging System (Bio-Rad Laboratories, Hercules, CA, USA) for visualization of the bands. The densitometric analysis of the obtained bands was conducted using ImageJ software (version 1.46r; National Institutes of Health; Bethesda, MD, USA).

### Droplet digital PCR (ddPCR) for mRNA expression

Absolute quantification of gene expression was performed using the ddPCR technique. NSCLC parental cell lines (C827/par and H1975/par) and their EGFR-TKI-resistant counterparts (C827/GEF, H1975/DAC, and H1975/OSI) were seeded in 12-well plates. When cells reached full confluence, total RNA was extracted using TRI reagent. The RNA concentration of the obtained samples was measured using the NanoDrop ND-1000 spectrophotometer (American Laboratory Trading; East Lyme, CT, USA). cDNA was synthesized using a two-step reverse transcription protocol (total volume of 20 µL), as previously described [33]. A total of 1 µg of RNA was hybridized with 5 µM oligo(dT) at 65 ˚C for 5 min in a T100 Thermal Cycler (Bio-Rad Laboratories, Hercules, CA, USA). Then, a mastermix consisting of 4 µL of 5 × ProtoScript^®^ II Reverse Transcriptase Reaction Buffer, 2 µL of 10 × dithiothreitol, 1 µL of 10 mM dNTP Solution Mix, and 0.5 µL of 200 U/µL ProtoScript^®^ II Reverse Transcriptase was added to the sample. The mixture was subjected to the following cycling protocol: 42 ˚C for 50 min followed by 65 ˚C for 20 min.

Next, TaqMan probes-based ddPCR experiments were conducted using the prepared cDNA. Droplets were generated in a total volume of 90 µL (25 ng cDNA/20 µL of reaction system and 70 µL of generation oil droplets) using the QX200 Droplet Generator (Bio-Rad Laboratories, Hercules, CA, USA) according to the manufacturer’s protocol. The generated droplets were carefully pipetted into 96-well PCR plates (40 µL/well) and sealed using the PX1 PCR Plate Sealer (Bio-Rad Laboratories, Hercules, CA, USA) at 180 ˚C. Sealed plates were placed into the T100 Thermal Cycler for PCR amplification under conditions recommended by the TaqMan probes’ supplier. Finally, plates were read, and the absolute number of gene copies was calculated using the QX200 Droplet Reader and its accompanying software QuantaSoft™ version 1.7 (Bio-Rad Laboratories, Hercules, CA, USA).

### DNA sequencing of EGFR

Sanger-based DNA sequencing, provided by SEQme (Dobris, Czech Republic), was used for the detection of clinically relevant *EGFR* mutations. Template cDNA samples were prepared as described above. A total of 25 µL of the reaction mixture, consisting of 5 µL of 5X Q5^®^ Reaction Buffer, 0.5 µL of 10 mM dNTPs, 5 µL of 5X Q5^®^ High GC Enhancer, 0.25 µL of Q5^®^ Hot Start High-Fidelity DNA Polymerase, 1 µL of template DNA, 1.25 µL of 10 µM F primer, 1.25 µL of 10 µM R primer, and 10.75 µL of Nuclease-Free Water, was transferred into an MJ Mini™ Thermal Cycler (Bio-Rad Laboratories, Hercules, CA, USA). PCR was performed according to the cycling protocol recommended by the polymerase manufacturer.

The PCR products were screened using agarose gel electrophoresis and subsequently purified with the NucleoSpin Gel and PCR Clean-Up Kit before being sent for DNA sequencing. The F and R primers, designed to amplify the target region of the *EGFR* gene and used for DNA sequencing, were synthesized by Generi Biotech (Hradec Králové, Czech Republic). The sequences of the primers were: F primer (5’ – 3’): CCGTCGCTATCAAGGAAT, and R primer (5’ – 3’): CTGCTGTGGGATGAGGTA.

### Bioinformatic analysis

The publicly available microarray raw data obtained from NSCLC patients were collected from Gene Expression Omnibus (GEO) databases (GSE31210) [39]. The groups were normalized and compared by using the free Affymetrix Expression Console Software provided by Thermo Fisher (Waltham, MA, USA).

### Statistical analysis

Statistical analyses of the obtained data were performed using a two-tailed unpaired *t*-test, or for the comparison of the gene expression within groups and intragroup, non-parametric ANOVA (Kruskal–Wallis test) and one-way ANOVA testing were used, respectively. The overall survival probabilities were estimated and presented by Kaplan–Meier survival curve with GraphPad Prism software version 9.4.1 (GraphPad Software Inc.; La Jolla, CA, USA). In bioinformatic analyses, *p*-values were automatically generated by the respective analytical platform. Statistical significance was considered when p-values were less than 0.05. The *p*-values in the presented tables, figures, and supplementary file are indicated as follows: ns (not significant), * *p* < 0.05, ** *p* < 0.01, *** *p* < 0.001, and **** *p* < 0.0001. Quantitative data are presented as mean ± SD. All experiments were independently performed at least three times.

## Results

### Neither EGFR expression changes nor additional mutations confer acquired resistance to gefitinib, dacomitinib, and osimertinib in developed NSCLC models

**Fig. 1:**
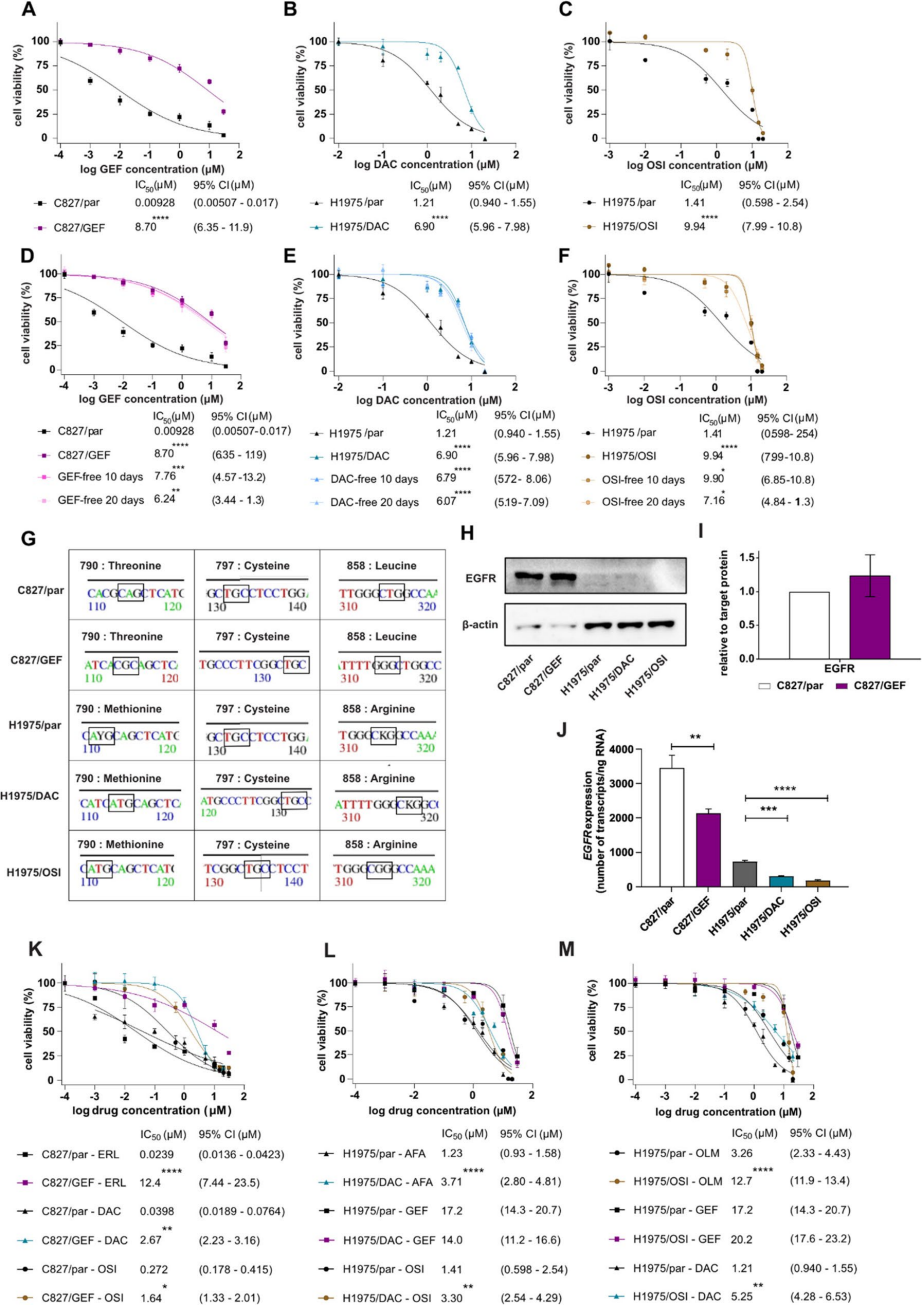
Acquired EGFR-TKI resistance after 10 months shows reduced *EGFR* expression without new *EGFR* mutations. **A-C** EGFR-TKI dose-response curves of parental (C827/par) and gefitinib-resistant (C827/GEF) C827 cells (**A**), parental (H1975/par) and dacomitinib-resistant (H1975/DAC) H1975 cells (**B**), as well as parental (H1975/par) and osimertinib-resistant H1975 cells (H1975/OSI) (**C**), representing the viabilities after 48-hour treatment with increasing concentrations of the corresponding EGFR-TKIs as indicated. **D**-**F** Cell viability curves for GEF-free, DAC-free, and OSI-free conditions (10 and 20 days post-treatment withdrawal) in C827/GEF, H1975/DAC, and H1975/OSI cells, illustrating the stability of resistance patterns. **G** Amino acid (AA) sequence at positions 790, 797, and 858 in EGFR for the C827 and H1975 parental and resistant cell lines, highlighting the presence of clinically relevant common activating mutation L858R and mutations associated with the resistance toward first and second/third EGFR-TKIs generations (T790M and C797S, respectively). **H** Representative images from EGFR protein detection by Western blotting in parental and resistant cell lines. **I** Related quantitative densitometric analysis in C827/par vs. C827/GEF cells, showing non-significant difference. **J***EGFR* mRNA expression levels, with significant reduction in GEF, DAC and OSI resistant cells compared to their parental counterparts. **K**-**M** MTT-based viability curves in C827 parental and GEF-resistant cells treated with erlotinib (ERL), DAC, and OSI (**K**), H1975 parental and DAC-resistant cells treated with afatinib (AFA), GEF, and OSI (**L**), and H1975 parental and OSI-resistant cells treated with olmutinib (OLM), GEF, and DAC (**M**), illustrating cross-resistance profiles

To systematically investigate acquired resistance to EGFR-TKIs and potential unique and universal resistance mechanisms across the EGFR-TKI generations, we established three resistant cell lines: C827/GEF (gefitinib-resistant), H1975/DAC (dacomitinib-resistant), and H1975/OSI (osimertinib-resistant) after 10 months-lasting selection procedure. Compared to the parental C827, C827/GEF displayed 938-fold increased gefitinib resistance (Fig. 1A), whereas H1975/DAC and H1975/OSI exhibited 5.70- and 7.05-fold increased resistance to dacomitinib and osimertinib, respectively, relative to H1975/par (Fig. 1B and C). Resistance stability was assessed by measuring IC_50_ values 10 and 20 days after drug withdrawal. Notably, in all the resistant cell lines, IC_50_ levels remained essentially unaltered upon the drug-free culture (Fig. 1D-F).

To explore possible target alterations behind the adaptative resistance mechanisms, we evaluated *EGFR* mutational status and expression. By DNA sequencing, we identified no T790M, C797S, or L858R mutations in C827/par or C827/GEF (Fig. 1G and S1A). In H1975/par, intrinsic T790M and L858R mutations were detected along with heterozygous *EGFR* wild-type subpopulation, with no evidence of C797S; this mutational profile remained unchanged after the acquisition resistance in H1975/DAC. In H1975/OSI, we observed the loss of heterozygosity in *EGFR* (amino acid 790 and 858) with and elimination of wild type subpopulation and the maintenance of T790M and L858R, while no additional C797S mutation was detected (Fig. 1G and S1B). Notably, ddPCR analysis showed significantly reduced *EGFR* mRNA expression in drug-resistant cell lines compared to the parental counterparts (Fig. 1J). In contrast, comparable EGFR levels were detected between C827/par and C827/GEF by Western blotting, whereas in the parental and resistant H1975 cells, EGFR was below detection limit (Fig. 1H, I). Moreover, EGFR expression ratios of 1.0207, 1.0082 and 0.9159 for C827/GEF to C827/par, H1975/DAC to H1975/par and H1975/OSI to H1975/par, respectively, were recorded in proteomics data (data stored in Zenodo repository, see Data availability section). In summary, these results suggest that the developed resistant NSCLC models did not acquire additional *EGFR* mutations or EGFR expression changes.

### Cross-resistance between EGFR-TKIs: gefitinib, dacomitinib, and osimertinib resistance confers reduced sensitivity to alternative EGFR inhibitors

To determine whether EGFR-TKI resistance is drug-specific or class-wide phenomenon, we conducted cross-resistance studies. Alternative EGFR-TKIs from each generation were tested in each resistant cell line, with results compared to their parental counterparts. Notably, gefitinib resistance in C827/GEF was associated with a 519-fold, 67.1-fold, and 6.03-fold reduction in erlotinib, dacomitinib, and osimertinib cytotoxicity, respectively (Fig. 1K). In H1975/DAC, we observed a 3.02- and 2.34-fold decreases in sensitivity toward the treatment with afatinib and osimertinib, respectively (Fig. 1L). Similarly, H1975/OSI exhibited 3.90- and 4.34-fold resistance to olmutinib and dacomitinib, respectively (Fig. 1M). However, no significant gefitinib-response difference was recorded in H1975/DAC and H1975/OSI, compared to H1975/par (Fig. 1L and M). These findings indicate that cross-resistance is a universal and robust phenomenon across all EGFR-TKI generations.

### Global proteomic analysis reveals target-independent resistance mechanisms in EGFR-TKI-resistant NSCLC cells

**Fig. 2:**
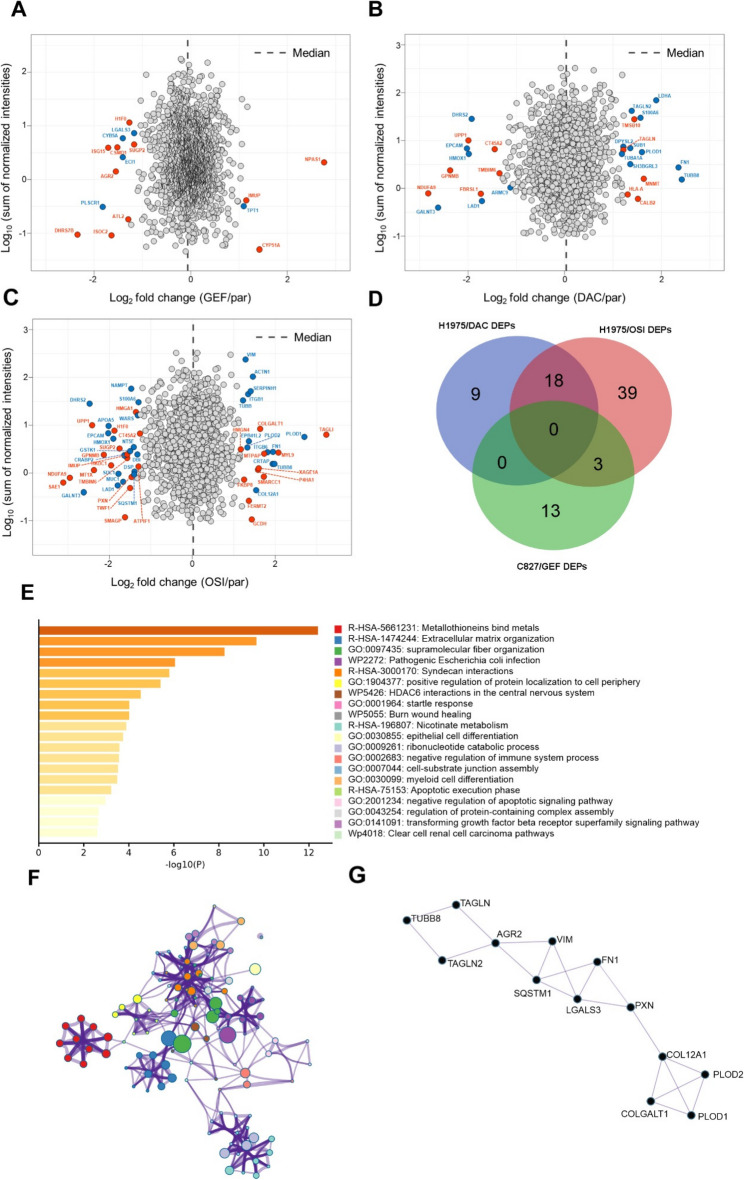
Proteomic analysis identifies shared and distinct resistance pathways in gefitinib, dacomitinib, and osimertinib-resistant cells. **A-C** Volcano plots showing the log2 fold change in protein expression between parental and treatment-resistant cell lines (GEF, DAC, OSI) versus log10 sum of normalized intensities. Key matrix related proteins are highlighted in C827/GEF (**A**), H1975/DAC (**B**), and H1975/OSI (**C**) comparisons. **D** Venn diagram illustrating the overlap of differentially expressed proteins (DEPs) between H1975/DAC, H1975/OSI, and C827/GEF cell lines. **E** Bar chart of significantly enriched pathways identified by functional enrichment analysis of shared DEPs, including pathways such as extracellular matrix organization (R-HSA-1474244), regulation of apoptotic signaling (GO-0042981), and metallothioneins binding metals (R-HSA-5661231). **F** Network analysis of the top enriched pathways from the shared DEP dataset, highlighting the interactions between key proteins and pathways involved in drug resistance mechanisms. **G** Interaction network of key shared DEPs involved in cytoskeletal organization, extracellular matrix remodeling, and cell adhesion, with connections between proteins such as TAGLN, FN1, VIM, and COL12A1

To explore possible activation of target-independent mechanisms governing the EGFR-TKI resistance, we conducted a global LC-MS/MS proteomic analysis to quantify protein expression in drug-resistant and parental NSCLC cell lines. A total of 1786–1793 proteins were quantified, and the differential expression patterns and the most differentially expressed proteins (DEPs) indicated in the scatter plots (Fig. 2A-C). These plots illustrate prominent and variable changes in protein expression upon the development of EGFR-TKI resistance, with specific proteins showing either significantly increased or decreased expression compared to the parental cell lines. Of note, H1975/DAC and H1975/OSI showed major overlap with 18 shared DEPs, whereas C827/GEF with the strongest fold-induction of resistance relative to parental cells, showed the lowest number of altered proteins and only shared three common DEPs with H1975/OSI (Fig. 2D). Full list of DEPs can be found in Zenodo repository (see Data availability section).

To gain insights into the biological processes and signaling pathways involved in the adaptive EGFR-TKI resistance, we performed pathway enrichment analysis using the shared DEPs. Markedly, extracellular matrix organization, cell adhesion and junctions, as well as apoptotic, differentiation and TGF-beta signaling, all related to cancer cell survival and metastasis, were represented among the enriched pathways (Fig. 2E). Moreover, network analysis of the most significantly altered proteins revealed key signaling hubs, including an interaction network focusing on proteins such as VIM, FN1, and PLOD1 (Fig. 2F and G). Along with the above enriched mechanisms, these may be used by NSCLC cells to evade the effects of EGFR-targeted therapies.

### BCL-2 overexpression contributes to EGFR-TKI resistance in NSCLC: potential for venetoclax combination therapy

**Fig. 3:**
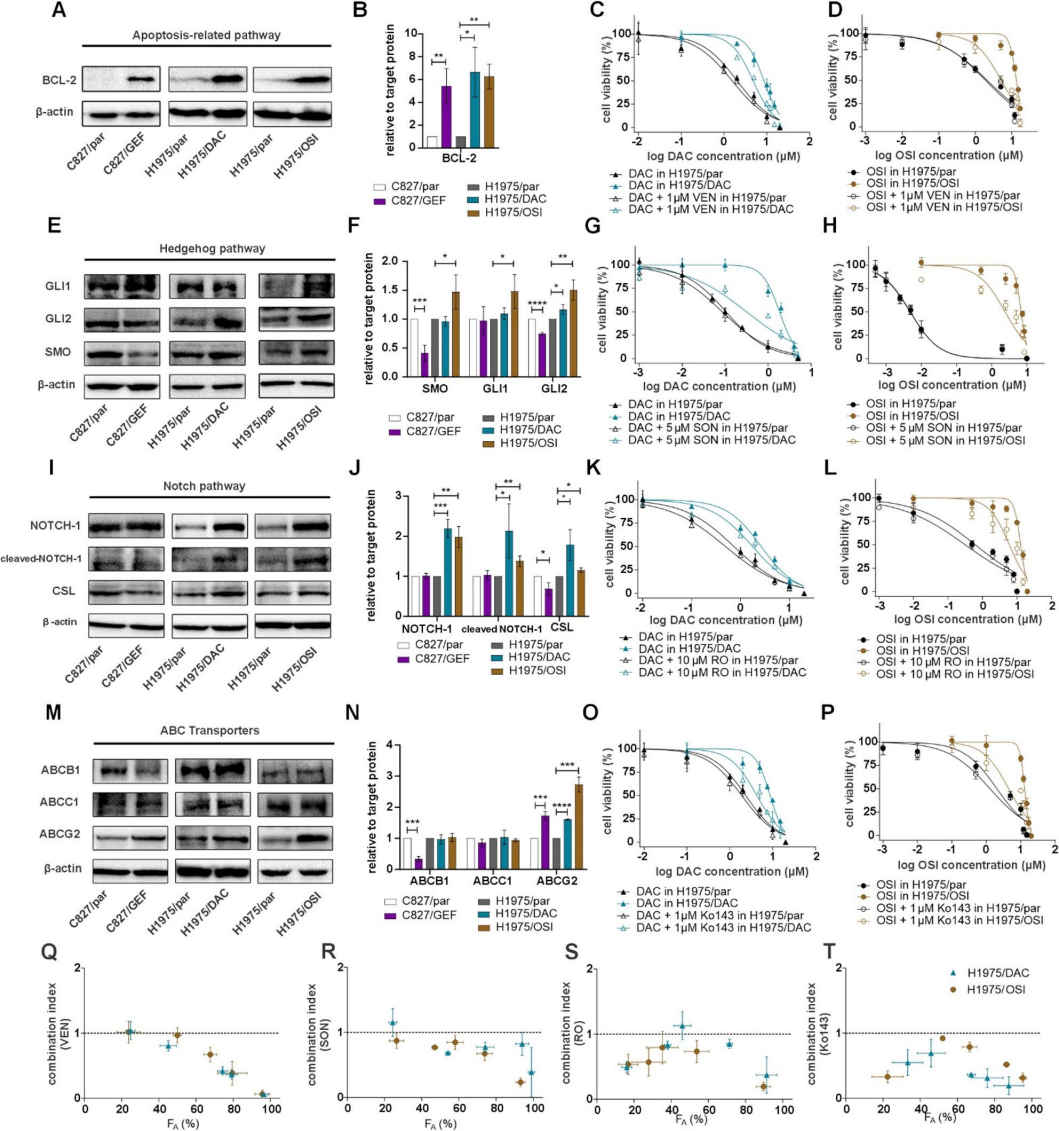
Targeting apoptotic, Hedgehog, Notch signaling pathways, and ABC transporters enhances sensitivity in EGFR-TKI-resistant cells. **A** Western blot analysis of BCL-2 expression in the apoptosis-related pathway across C827 and H1975 cell lines, including treatment-resistant variants (GEF, DAC, and OSI), with β-actin used as a loading control. **B** Quantification of BCL-2 protein expression relative to β-actin. (**C-D**) Dose-response curves showing cell viability with increasing concentrations of dacomitinib (**C**) and osimertinib (**D**) in H1975 parent cell line along with DAC- and OSI-resistant sublines, with and without 1 µM venetoclax (VEN) treatment. **E** Western blot analysis of Hedgehog pathway proteins SMO, GLI1, and GLI2 in C827 and H1975 cell lines, with β-actin as a loading control. **F** Quantification of Hedgehog pathway protein expression relative to β-actin. **G**-**H** Dose-response curves showing cell viability with increasing concentrations of dacomitinib (**G**) and osimertinib (**H**) in H1975 cells, including resistant sublines, with and without 5 µM sonidegib (SON) treatment. **I** Western blot analysis of Notch pathway proteins NOTCH-1, cleaved NOTCH-1, and CSL in C827 and H1975 cell lines, with β-actin as a loading control. **J** Quantification of Notch pathway protein expression relative to β-actin. (K–L) Dose-response curves showing cell viability with increasing concentrations of dacomitinib (**K**) and osimertinib (**L**) in H1975 parental and H1975 resistant cell lines, with and without 10 µM RO4929097 (RO) treatment. **M** Western blot analysis of ABC transporters ABCB1, ABCC1, and ABCG2 across C827 and H1975 parental and resistant cell lines, with β-actin used as a loading control. **N** Quantification of ABC transporter protein expression relative to β-actin. **O-P** Dose-response curves showing cell viability with increasing concentrations of dacomitinib (**O**) and osimertinib (**P**) in H1975 parent and resistant cell lines with and without 1 µM Ko143 treatment. (Q–T) Combination index (CI) plots for various treatment combinations in H1975 cell lines, showing drug combination effects across the range of fractional of cells affected (F_A_)

With respect to the results from pathway analysis (Fig. 2E), we started the mechanistic validation from apoptotic-related pathways. By ddPCR of six apoptosis-related genes, we revealed a significant increase in *BCL2* mRNA in all tested drug-resistant models compared to the corresponding parental cell lines (Fig. S2A and B). This increase in *BCL2* expression was further confirmed at the protein level through western blotting, which showed elevated BCL-2 protein levels in C827/GEF, H1975/DAC, and H1975/OSI cell lines (Fig. 3A and B).

To test if this BCL-2 overexpression provides a target for resistance-reversal, we used the selective, clinically used BCL-2 inhibitor, venetoclax. The drug combination studies demonstrated that inhibiting BCL-2 significantly enhanced the cytotoxicity of dacomitinib in the H1975/DAC cell line (Resistance Ratio (R_R_) = 1.82), and to a lesser extent in its parental counterpart (R_R_ = 1.33). Similarly, a combination of venetoclax and osimertinib significantly enhanced the cytotoxicity in H1975/OSI cells (R_R_ = 2.64), though no significant shift in IC_50_ was observed in the parental cells. However, venetoclax failed to induce the sensitization of cancer cells to gefitinib (insignificant changes in C827/par and C827/GEF) (Fig. 3C and D, Fig. S2C and Table 1).

**Table 1:** IC_50_-shift analysis for resistance-reversal studies

Target (Inhibitor)	Cell line	Single drug or NC IC_50_ ± SD (µM)	Drug combination or siRNA IC_50_ ± SD (µM)	*R*_*R*_
FAK (DEF)				
	C827/P-GEF	0.00950 ± 0.000623	0.00740 ± 0.000451^*^	1.28
	C827/R-GEF	14.7 ± 1.05	1.37 ± 0.286^**^	10.7
	H1975/P-DAC	1.99 ± 0.0702	1.30 ± 0.357^ns^	1.53
	H1975/R-DAC	6.36 ± 0.528	1.56 ± 0.184^**^	4.08
	H1975/P-OSI	1.72 ± 0.212	1.47 ± 0.216^ns^	1.17
	H1975/R-OSI	11.0 ± 0.226	3.27 ± 0.388^****^	3.36
YAP1 (VER)				
	C827/P-GEF	0.0101 ± 0.000653	0.00710 ± 0.00107^*^	1.42
	C827/R-GEF	13.4 ± 1.289	0.687 ± 0.270^**^	19.6
	H1975/P-DAC	1.41 ± 0.489	1.19 ± 0.293^ns^	1.18
	H1975/R-DAC	6.92 ± 1.09	3.40 ± 0.466^*^	2.04
	H1975/P-OSI	1.40 ± 0.167	1.18 ± 0.121^ns^	1.19
	H1975/R-OSI	11.2 ± 0.573	4.87 ± 0.705^***^	2.30
FN1 (siRNA-FN1)				
	H1975/P-DAC	0.683 ± 0.0708	0.631 ± 0.190^ns^	1.08
	H1975/R-DAC	1.41 ± 0.481	0.127 ± 0.0383^*^	11.1
	H1975/P-OSI	0.431 ± 0.108	0.318 ± 0.0786^ns^	1.36
	H1975/R-OSI	6.82 ± 0.616	0.582 ± 0.201^****^	11.7
BCL-2 (VEN)				
	C827/P-GEF	0.0121 ± 0.00109	0.0101 ± 0.000291^ns^	1.20
	C827/R-GEF	16.4 ± 1.12	14.8 ± 1.05^ns^	1.11
	H1975/P-DAC	2.02 ± 0.0695	1.52 ± 0.201^*^	1.33
	H1975/R-DAC	8.73 ± 0.285	4.80 ± 0.278^****^	1.82
	H1975/P-OSI	2.11 ± 0.282	1.91 ± 0.272^ns^	1.10
	H1975/R-OSI	13.3 ± 0.265	5.03 ± 0.456^****^	2.64
Hedgehog (SON)				
	H1975/P-DAC	0.103 ± 0.0143	0.0858 ± 0.0256^ns^	1.20
	H1975/R-DAC	1.85 ± 0.225	0.321 ± 0.0927^**^	5.76
	H1975/P-OSI	0.00497 ± 0.000507	0.00485 ± 0.000156^ns^	1.02
	H1975/R-OSI	6.87 ± 0.522	2.51 ± 0.691^**^	2.74
Notch (RO)				
	H1975/P-DAC	0.747 ± 0.205	0.498 ± 0.0149^ns^	1.50
	H1975/R-DAC	2.65 ± 0.111	1.77 ± 0.208^**^	1.50
	H1975/P-OSI	0.657 ± 0.229	0.342 ± 0.118^ns^	1.92
	H1975/R-OSI	12.8 ± 0.360	6.27 ± 2.42^*^	2.04
ABCG2 (Ko143)				
	C827/P-GEF	0.0124 ± 0.000616	0.0101 ± 0.00211^ns^	1.23
	C827/R-GEF	15.9 ± 0.945	14.6 ± 0.504^ns^	1.08
	H1975/P-DAC	2.33 ± 0.346	1.83 ± 0.759^ns^	1.27
	H1975/R-DAC	8.67 ± 0.210	4.29 ± 0.653^**^	2.02
	H1975/P-OSI	2.17 ± 0.678	1.32 ± 0.355^ns^	1.64
	H1975/R-OSI	13.0 ± 0.244	4.27 ± 0.848^**^	3.04

**Fig. 4:**
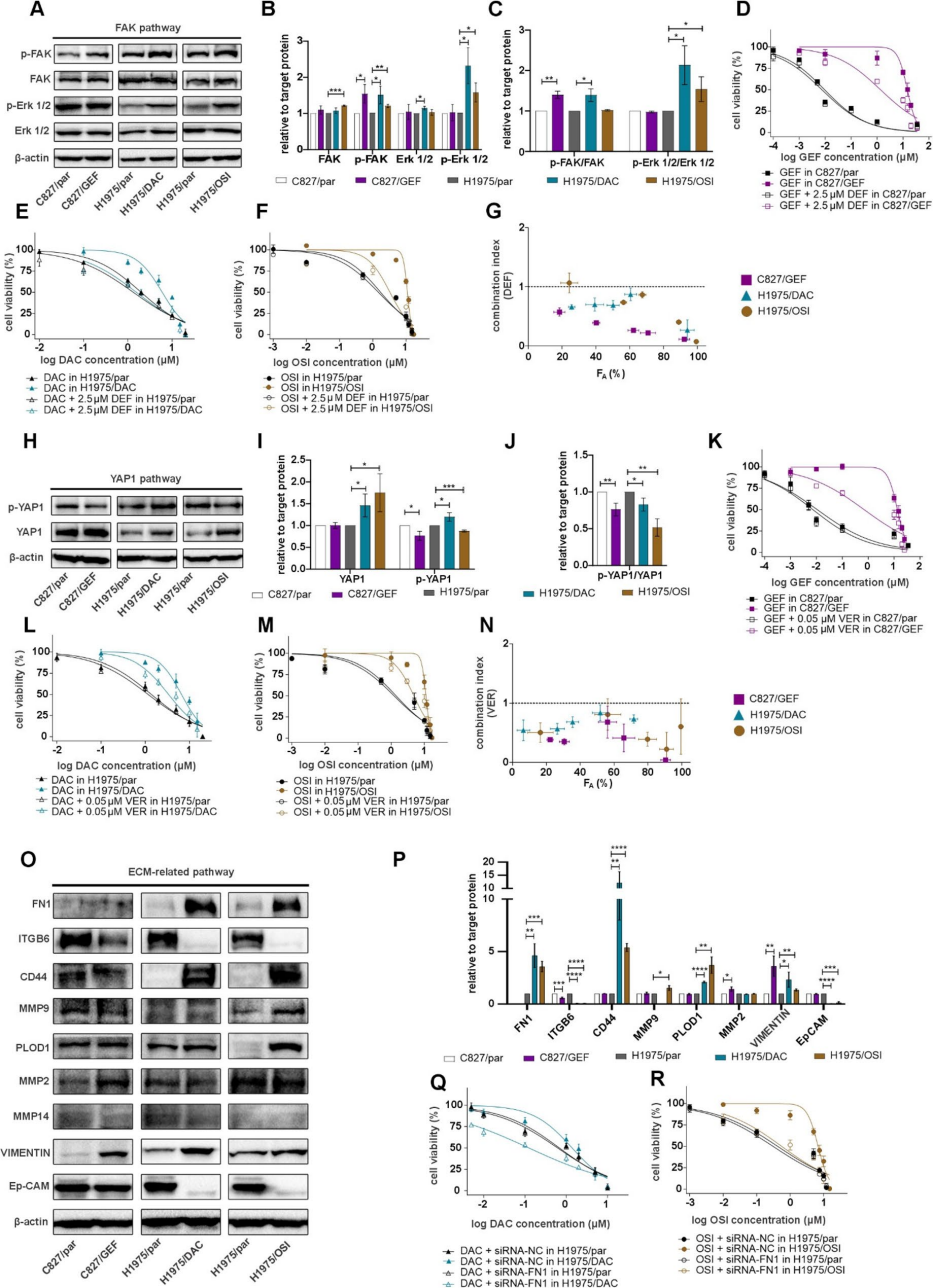
Targeting FAK, YAP1, and FN1 signaling to overcome EGFR-TKI resistance in NSCLC cells. **A** Western blot analysis of the FAK pathway in C827 and H1975 cell lines, including treatment-resistant variants (GEF, DAC, and OSI). Blots show p-FAK, FAK, p-Erk1/2, and Erk1/2, with β-actin as a loading control. **B-C** Quantification of protein expression in the FAK pathway for (**B**) C827 and (**C**) H1975 cell lines, showing the ratio of phosphorylated to total protein. **D-F** Dose-response curves showing cell viability with increasing concentrations of (**D**) gefitinib, (**E**) dacomitinib, and (**F**) osimertinib for various cell lines, including conditions with 2.5 µM defactinib (DEF) treatment. **G** CI plot for DEF combination with different cell lines at varying F_A_ levels. **H** Western blot analysis of the YAP1 pathway in C827 and H1975 cell lines, including GEF, DAC, and OSI variants, showing p-YAP1 and YAP1 protein levels. β-actin is used as a loading control. **I-J** Quantification of YAP1 and p-YAP1 protein levels. **K**-**M** Dose-response curves for cell viability with increasing concentrations of (**K**) gefitinib, (**L**) dacomitinib, and (**M**) osimertinib in the presence of 0.05 µM verteporfin (VER). **N** CI plot for VER combination across different cell lines at varying F_A_ levels. **O** Western blot analysis of the ECM-related pathway in C827 and H1975 cell lines, including GEF, DAC, and OSI variants, showing expression of proteins FN1, ITGB6, CD44, MMP9, PLOD1, MMP2, MMP14, VIMENTIN, and Ep-CAM, with β-actin as a loading control. **P** Quantification of ECM-related protein expression. **Q-R** Dose-response curves for cell viability with increasing concentrations of (**Q**) dacomitinib and (**R**) osimertinib in the presence of siRNA knockdown of FN1 in H1975 parental and resistant cell lines

IC_50_ values were calculated based on the cell viability results from Figs. 3 and 4 and Fig. S2. Two-tailed unpaired *t*-test was applied for determining statistical difference between IC_50_ values within particular sublines (compared to gefitinib/dacomitinib/osimertinib alone). Reversal ratio (R_R_) is used to evaluate the extent of drug resistance reversal from the combination treatment in each of the cell line. R_R_ was calculated according to the formula: R_R_ = IC_50 single drug_/IC_50 drug combination_

*DAC* Dacomitinib, *DEF* Fefactinib, *GEF* Gefitinib, *OSI* Osimertinib, *RO* RO4929097, *SON* Sonidegib, *VEN* Venetoclax, *VER* Verteporfin, *NC* Negative control, *P* Parental, *R* Resistant, ^*^p < 0.05, ^**^ *p* < 0.01, ^***^p < 0.001, ^****^p < 0.0001, ^ns^ not significant

Additionally, Chou-Talalay analysis of the drug combination effects revealed that the combinations of venetoclax with EGFR-TKIs were predominantly defined as synergism in dacomitinib- and osimertinib-resistant cell lines (Fig. 3Q). These results suggest that BCL-2 plays a critical role in EGFR-TKI resistance and may represent an effective therapeutic target for overcoming resistance to dacomitinib and osimertinib in NSCLC.

### Targeting cancer stemness-related pathways: Hedgehog and Notch inhibition restores sensitivity to EGFR-TKIs in drug-resistant NSCLC cells

Cancer stem cells (CSCs), known for their self-renewal potential and intrinsic drug resistance, often form the “invincible” tumor core in various cancers [40]. Considering connections of stem cell regulation to “burn wound healing” and the “transforming growth factor beta receptor superfamily signaling” pathways found in our results (Fig. 2E), we investigated whether CSC-related signaling pathways contribute to EGFR-TKI resistance. Western blot quantification revealed significant upregulation of the Hedgehog pathway proteins GLI1, GLI2 and SMO in H1975/DAC and H1975/OSI cells compared to their parental counterparts. In contrast, no significant changes in the expression of key Hedgehog pathway proteins were observed in the gefitinib-resistant C827/GEF (Fig. 3E and F). Combination treatment with sonidegib, a clinically used Hedgehog pathway inhibitor, demonstrated significant re-sensitization of the cells resistant to dacomitinib and osimertinib, with R_R_s of 5.76 for H1975/DAC and 2.74 for H1975/OSI. However, in parental cells (H1975/par), sonidegib did not significantly enhance the cytotoxicity of either EGFR-TKI (Fig. 3G and H; Table 1).

Similarly, the levels of both NOTCH1, and cleaved NOTCH1 as well as CSL were significantly increased in H1975/DAC and H1975/OSI (Fig. 3I and J). Treatment with RO4929097, a Notch pathway inhibitor, restored the responsiveness of H1975/DAC (R_R_ = 1.50) and H1975/OSI (R_R_ = 2.04) to the antiproliferative effects of the respective EGFR-TKIs (Fig. 3K and L; Table 1).

Resistance-reversal data from Hedgehog and Notch pathways were further analyzed using the Chou-Talalay method to quantify combination effects. Synergistic effects were observed across a broad range of the fractional affected (F_A_) in all analyzed drug combinations for H1975/DAC and H1975/OSI (Fig. 3R and S). Taken together, these results suggest that blockade of key CSC-related pathways can alleviate EGFR-TKI resistance, a strategy that may be applicable to advanced generations of EGFR-TKIs.

### ABCG2 overexpression contributes to EGFR-TKI resistance: potential for Ko143-mediated sensitization in higher-generation inhibitors

Apart from the pharmacodynamic factors, ATP-binding cassette (ABC) drug efflux transporters are often linked to the development of multidrug resistance and poor prognosis in cancer patients [41, 42]. Therefore, we next investigated possible contribution of pharmacokinetic mechanisms to the establishment of EGFR-TKI resistance. Western blot analysis revealed that the protein expression levels of ABCG2 were significantly increased in all three EGFR-TKI-resistant cell lines (Fig. 3M and N).

Our data showed that Ko143, a model inhibitor of ABCG2, significantly sensitized H1975/DAC (R_R_ = 2.02) and H1975/OSI (R_R_ = 3.04) to dacomitinib and osimertinib, respectively. However, Ko143 failed to enhance the anticancer activity of gefitinib in C827/GEF, with non-significant IC_50_ shifts observed in both parental cell lines (Fig. 3O and P, Fig. S2D and Table 1).

Further analysis of the data using the Chou-Talalay algorithm demonstrated synergistic effects when Ko143 was combined with EGFR-TKIs in dacomitinib- and osimertinib-resistant cell lines (Fig. 3T). Taken together, these findings suggest that ABCG2 may be an important pharmacokinetic resistance factor that universally affects the antitumor activities of higher-generation EGFR-TKIs.

### ECM signaling pathways FAK, YAP1, and FN1 as universal targets for overcoming EGFR-TKI resistance

Our bioinformatic analysis ranked “Extracellular matrix organization” as the second most involved pathway, underscoring its dominant role in the acquired EGFR-TKI resistance (Fig. 2E). Evidence suggests that FAK and YAP1 signaling actively regulate ECM dynamics and, consequently, the tumor microenvironment [43, 44]. Significantly, phosphorylated FAK (p-FAK) was increased as a universal consequence of resistance development for EGFR-TKIs across all three generations. Additionally, the levels of phosphorylated Erk 1/2 (p-Erk 1/2) and p-Erk 1/2 to Erk 1/2 ratio were significantly elevated in both dacomitinib- and osimertinib-resistant cell lines (Fig. 4A-C).

To test whether FAK signaling might be a druggable target to counter EGFR-TKI resistance, we conducted resistance reversal assays. Markedly, defactinib, a specific inhibitor of FAK signaling, effectively potentiated the sensitivities of all three drug-resistant cell lines to EGFR-TKI, with R_R_ of 10.7 in C827/GEF, 4.08 in H1975/DAC, and 3.36 in H1975/OSI. In contrast, no significant IC_50_ shifts were observed in parental cell lines, with a slight R_R_ of 1.28 observed in C827/par cells under co-treatment with gefitinib and defactinib (Fig. 4D-F; Table 1). The combination effects of defactinib with EGFR-TKIs were quantified using the Chou-Talalay method, which revealed synergistic effects in all three resistant cell models (Fig. 4G).

Downstream from FAK, both H1975/DAC and H1975/OSI cells showed significant increases in YAP1 expression, with a corresponding decrease in the ratio of phosphorylated YAP1 (p-YAP1)/YAP1 compared to H1975/par. In C827/GEF cell line, despite of a non-significant change of total YAP1 expression, p-YAP1 was significantly reduced, leading to a significant decrease in the p-YAP1/YAP1 ratio (Fig. 4H-J). As a result, YAP1 signaling was overactivated in all three EGFR-TKI-resistant cell lines. Combination assays showed that verteporfin, an inhibitor of YAP1 signaling, significantly alleviated resistance in C827/GEF (R_R_ = 19.6), H1975/DAC (R_R_ = 2.04), and H1975/OSI (R_R_ = 2.30) cells to their respective EGFR-TKIs (Fig. 4K-M; Table 1). Synergistic effects were confirmed by Chou-Talalay analysis across the entire F_A_ range in all drug-resistant variants (Fig. 4N). However, verteporfin had minimal or negligible effects on the sensitivity of parental cell lines to EGFR-TKIs’ treatments (Fig. 4K-M; Table 1).

In our proteomic data, FN1 was as possibly aberrantly overexpressed in dacomitinib- and osimertinib-resistant cell lines (Fig. 2B and C). Bioinformatics analysis suggested that dysregulation of ECM-associated signaling, with FN1 as a critical factor, may play an essential role in EGFR-TKI resistance. Western blot analysis of ECM-related proteins further revealed that several ECM signaling proteins, including FN1 among the proteomics hits increased in H1975/DAC and H1975/OSI, were upregulated particularly in these resistant cells (Fig. 4O and P). Following FN1 knockdown using siRNA, H1975/DAC and H1975/OSI cells exhibited significantly reduced tolerance to dacomitinib and osimertinib, respectively (Fig. 4Q and R; Table 1).

In summary, ECM and its associated signaling networks were found to be significantly involved in the establishment of resistance to all generations of clinically used EGFR-TKIs. FAK, YAP1, and FN1 signaling pathways were identified as promising and universal targets for overcoming EGFR-TKI resistance.

### Enhanced invasive and metastatic potentials of EGFR-TKI-resistant NSCLC cells in 3D collagen-rich cultures

**Fig. 5:**
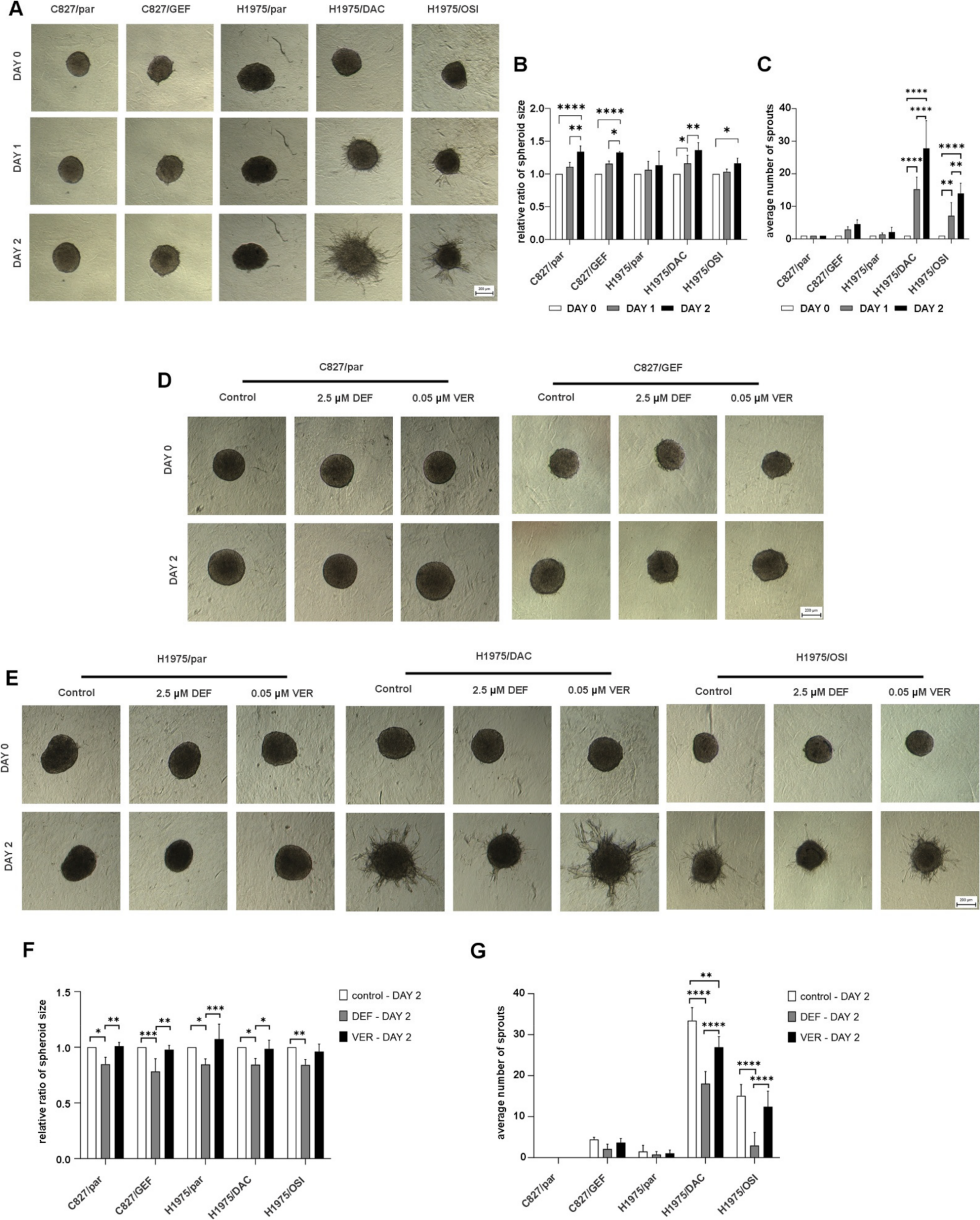
Impact of FAK and YAP1 inhibition on spheroid formation, growth, and invasiveness in EGFR-TKI-resistant cells. **A** Representative images of spheroids derived from C827 and H1975 cell lines, including treatment-resistant variants: gefitinib-resistant (GEF), dacomitinib-resistant (DAC), and osimertinib-resistant (OSI), over time (Day 0, Day 1, and Day 2). Scale bar: 200 μm. **B** Quantification of relative spheroid size (without peripheral sprouts; reflecting spheroid growth) in C827 and H1975 cell lines, including treatment-resistant variants, across three days. **C** Average number of peripheral sprouts (characterizing collagen invasive potential) per spheroid in different cell lines, including treatment-resistant variants, over two days. **D** Spheroid morphology of C827 cell lines treated with control, 2.5 µM defactinib (DEF), and 0.05 µM verteporfin (VER) at Days 0 and 2. **E** Spheroid morphology of H1975 cell lines, including dacomitinib-resistant and osimertinib-resistant cells, under control, 2.5 µM DEF, and 0.05 µM VER treatments at Days 0 and 2. Scale bar: 200 μm. **F** Quantification of relative spheroid size in C827 and H1975 cell lines, including treatment-resistant variants, treated with DEF and VER on Day 2, compared to control. **G** Average number of invasive sprouts per spheroid in C827 and H1975 cell lines, including treatment-resistant variants, under DEF and VER treatments on Day 2

To assess the relation of the above pathway alterations to the ECM-dependent call phenotypes and functions, we cultured the resistant cells as 3D spheroids in a collagen environment. After 2 days of cultivation, the spheroids exhibited remarkably varying degrees of morphological changes (Fig. 5A). The obtained results were quantified by measuring the size of the core part of spheroids (without sprouts, reflecting spheroid growth) and the number of sprout-like structures growing on the periphery (characterizing invasive and metastatic potentials). Significant increases in the core size of tumoroids were observed in all tested sublines, except for H1975/par (Fig. 5B). Furthermore, H1975/DAC and H1975/OSI formed a significantly higher number of sprouts compared to their corresponding parental cells. Over time, sprouting of C827/GEF spheroids also showed an increasing trend, although nonsignificant (Fig. 5C). Taken together, these data suggested that NSCLC cells exhibit substantial invasive/metastatic potentials after developing resistance to EGFR-TKIs, especially to drugs from higher generations.

### FAK signaling regulates invasive and metastatic behaviors in EGFR-TKI-resistant NSCLC cells

In addition to the proliferation-oriented studies described previously, defactinib and verteporfin were utilized to further investigate whether FAK and YAP1 pathways contribute to the invasive properties of drug-resistant cells and whether these characteristics can be reversed. Following 48-hour treatment, spheroid cultures exhibited varying degrees of morphological changes. The most pronounced alterations were observed in defactinib-treated H1975/DAC and H1975/OSI cell lines (Fig. 5E), whereas C827/GEF cells showed only limited changes compared to baseline (Fig. 5D). Treatment with defactinib resulted in a significant reduction in the relative size of the spheroids in all three drug-resistant cell lines compared to the inhibitor-free control group. In contrast, verteporfin did not significantly affect spheroid size in any of the models (Fig. 5F). Additionally, defactinib treatment significantly suppressed the formation of sprout-like structures in both the dacomitinib- and osimertinib-resistant 3D culture models. While verteporfin also reduced the number of sprouts in H1975/DAC, this effect was less pronounced than that observed with defactinib (Fig. 5G). These results indicate that FAK signaling, rather than YAP1 signaling, plays a crucial role in the invasive and metastatic behaviors of EGFR-TKI-resistant cells and that targeting FAK may be an effective strategy to modulate these behaviors. This observation appears to be applicable to both second- and third-generation EGFR-TKIs.

### ECM-related genes are associated with the poor prognosis and treatment outcomes in EGFR-mutant NSCLC patients

**Fig. 6:**
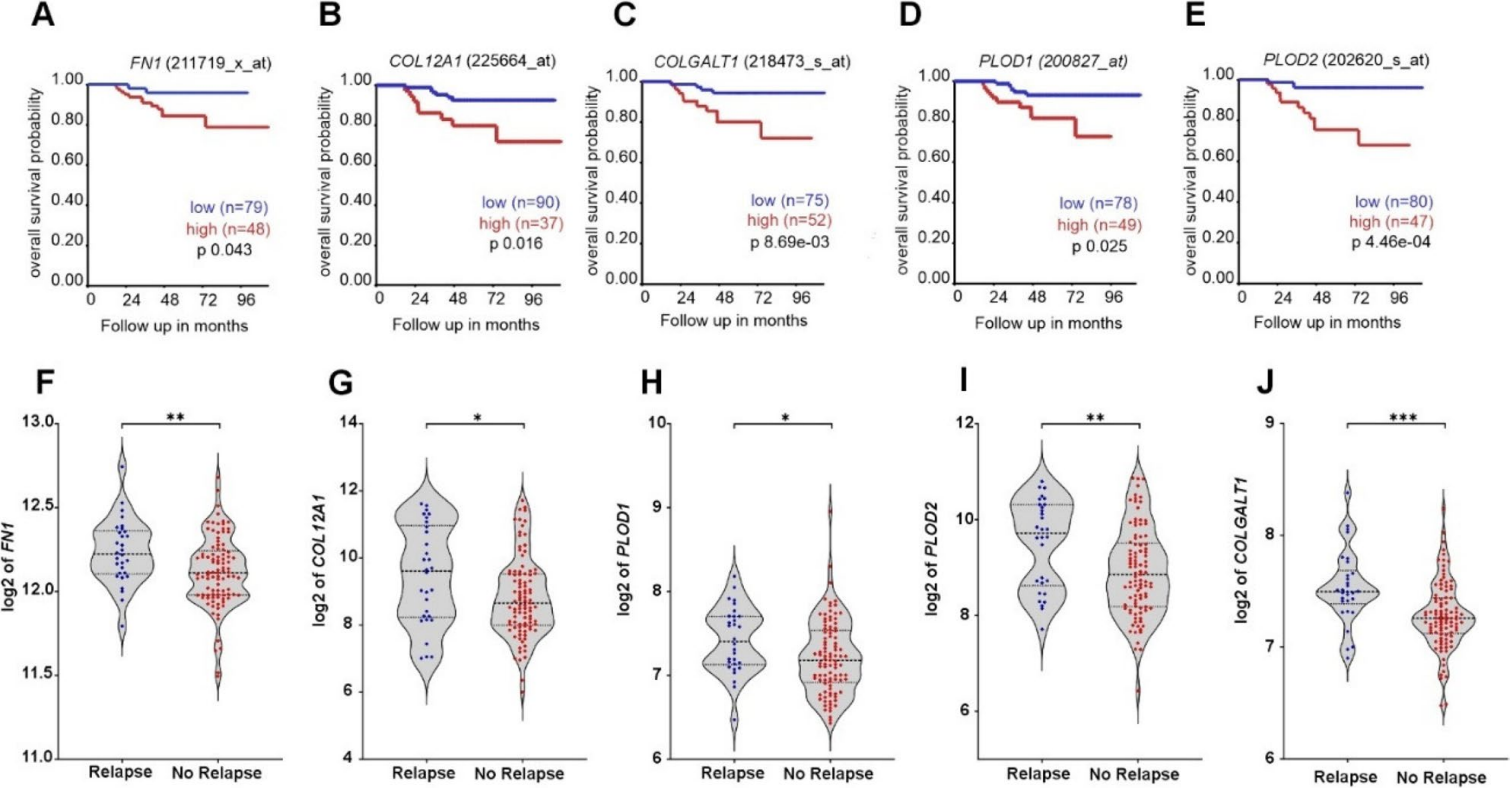
Prognostic significance and expression patterns of ECM-related genes in EGFR-mutant, treatment-resistant NSCLC patients. Kaplan-Meier survival curves for overall survival probability based on expression levels of (**A**) *FN1*, (**B**) *COL12A1*, (**C**) *COLGALT1*, (**D**) *PLOD1*, and (**E**) *PLOD2*. Patients bearing EGFR mutation were divided into low (blue) and high (red) expression groups for each gene, with log-rank p-values indicating significant differences in survival. **F-J** Violin plots of log2-transformed expression levels for (**F**) *FN1*, (**G**) *COL12A1*, (**H**) *PLOD1*, O) *PLOD2*, and (**J**) *COLGALT1*, comparing patients experiencing relapse versus no relapse

The interaction network of ECM-related proteins identified through proteomics data in EGFR-TKI-resistant cell lines highlights several genes and proteins involved in treatment resistance, particularly in EGFR-mutant subtypes of NSCLC. To determine the prognostic value of these ECM-related genes, specifically in EGFR-mutant NSCLC, we analyzed the public transcriptomic dataset GSE31210. Kaplan-Meier analysis revealed that higher expression of the gene signature was significantly associated with poor overall survival in this patient subgroup. This suggests these proteins play a pivotal role in the aggressive progression of EGFR-mutant NSCLC. *FN1*, a major extracellular matrix protein involved in cell adhesion, migration, and survival, was found to be associated with poor survival outcomes (*p* = 0.043) (Fig. 6A), highlighting its potential role in promoting the aggressive nature of these cancers. Further survival analysis of other ECM genes identified in the network revealed similarly significant findings. Elevated expressions of *COL12A1* (*p* = 0.016) (Fig. 6B), *COLGALT1* (*p* = 0.0008) (Fig. 6C), *PLOD1* (*p* = 0.025) (Fig. 6D), and *PLOD2* (*p* = 0.00004) (Fig. 6E) were all significantly associated with poorer overall survival, reinforcing the significant role of these ECM genes in the progression of EGFR-mutant NSCLC.

Given that most NSCLC patients experience relapse, which is related to treatment resistance and poor survival (Fig. S4A), further investigation of these ECM proteins in relapse patients revealed that *FN1*, *COL12A1*, *PLOD1*, *PLOD2*, and *COLGALT1* were significantly upregulated in relapsed tumors compared to non-relapsed tumors (Fig. 6F-J). This emphasizes the critical role of these ECM genes in the relapse and aggressive nature of EGFR-TKI-resistant NSCLC. The upregulation of these proteins in relapsed tumors suggests that they may be involved in the maintenance and progression of resistance mechanisms, providing potential targets for therapeutic intervention in the context of relapse. Overall, our findings highlight the importance of ECM-related genes in driving the progression, treatment resistance, and relapse of EGFR-mutant NSCLC, suggesting that targeting these proteins could offer new therapeutic strategies to improve patient outcomes in this subset of cancer.

## Discussion

Acquired drug resistance has greatly limited the efficacies of EGFR-TKIs in NSCLC treatment. In addition to drug-induced EGFR mutations, EGFR-independent mechanisms, involving alterations in resistance-associated signaling networks [20], has been proposed as significant contributors, though their impacts on tumor defense remain unclear. Furthermore, no single study has examined potential joint resistance mechanisms shared across EGFR-TKI generations yet. ECM, a network enriched with proteins and carbohydrates, is essential for maintaining the structural and biochemical integrity of tissues. Modifications in ECM composition are closely linked to tumor growth, survival, and metastasis, underscoring its potential as a therapeutic target [21]. The main aim of our study was to identify shared EGFR-TKI resistance mechanisms across three clinically used drug generations to potentially enable the development of “one-hit-for-all” therapeutic strategies. Specifically, we explored EGFR-dependent and -independent mechanisms contributing to resistance to gefitinib, dacomitinib, and osimertinib, and assessed the potential of ECM-related pathways as a central hub for overcoming resistance in NSCLC.

We established three drug-resistant NSCLC cell lines through long-term exposure to EGFR-TKIs. According to the clinical guidelines, all NSCLC patients should be routinely stratified based on their EGFR mutational profile prior to receiving EGFR-TKI treatments. Patients with basic receptor mutations are usually treated with first generation drugs, whereas higher generations serve as rescue drugs for the therapy of tumors with acquired mutations [45]. To mimic this essential clinical pattern, we chose two parental cell lines with distinct genetic backgrounds for different EGFR-TKI generations. The C827 cell line, with a basic activating *EGFR* exon 19 deletion mutation, was selected as the model for gefitinib. The H1975 cell line harboring basic L858R and acquired resistance-related T790M mutations was applied for the study with dacomitinib and osimertinib. Noteworthy, using a single parental cell line would allow us to better distinguish background-specific effects from mechanisms that are truly universal across EGFR-TKI generations; however, such an approach would suffer from clinical irrelevance.

Target-dependent mechanisms of EGFR-TKI resistance mainly consist of alterations in the expression of EGFR and/or its secondary mutation(s) [5]. Importantly, in the corresponding drug-resistant models, we did not record the acquisition of secondary gatekeeper T790M or C797S mutations, which are associated with resistance to gefitinib and osimertinib, respectively. Interestingly, we found that H1975 cells lost the wild-type signature at EGFR codon 790 and 858 after generating osimertinib resistance. This loss the heterozygosity might be caused by the selective pressure from osimertinib. EGFR-T790M- and EGFR-L858R-bearing cells are demonstrated to have stronger proliferative abilities than the EGFR wild type ones. Long-term treatment might favor the mutant subpopulation and eventually eliminate the wild-type subpopulation [46, 47]. However, our data suggests that our cell lines represent a unique transitional phase before achieving irreversible mutation-based resistance, a condition that may persist for months in patients. Targeting EGFR-independent mechanisms might be a promising strategy for preventing or overcoming resistance in this phase. Interestingly, significant downregulation of *EGFR* was recorded in all resistant cells, while no significant difference was observed at protein expression level. Within the establishment of drug resistance (including to EGFR-TKIs), cells usually either down-regulate or, conversely, amplify the target to avoid the drug’s action [48–50]. The resistant cells can turn on bypass signaling [51, 52], while retaining or even upregulating the primary target, which loses its oncogenic function. This is frequently not due to transcriptional changes, but increased protein stability or other mechanisms. Such adaptive energy-saving strategy is commonly associated with a dynamic transitional EMT state lacking genetic fixation (i.e., additional acquired target mutations resulting in fully stable resistance) [52, 53]. It allows drug-tolerant persisters to keep “cellular fitness” and the ability to switch back to rely on the target as the key cancer progression driver as soon as the selection pressure of the drug subsides. Comparing this common pattern to our results, we see complete overlap (transitional state without additive genetic fixation, and with upregulated ECM-signaling, suggesting EMT or pre-EMT state; several upregulated bypass pathways/proteins, functionally validated in drug combinations; retained protein expression of target). This concept is consistent with our contradictory results for *EGFR* mRNA and EGFR protein expression.

Cross-resistance may concomitantly occur when cancer cells lose their sensitivities to one drug, thus limiting treatment options across different therapies. Our resistant models exhibited strong cross-resistance to EGFR-TKIs across all generations, except for gefitinib in H1975 cells. The absence of a cross-resistance pattern in this case likely results from the strong intrinsic mutation-based (T790M) resistance in H1975 cells [15], which overwhelms other acquired mechanisms. This hypothesis is supported by the high, clinically irrelevant IC₅₀ of gefitinib observed in the parental H1975 model. Previous studies have reported that osimertinib-resistant cell lines also developed resistance to erlotinib and afatinib, while gefitinib-resistant cells were less sensitive to both afatinib and osimertinib [54, 55]. These findings, coupled with our results, indicate that cross-resistance is a common feature of EGFR-TKIs. This has important clinical implications, suggesting that resistance to current EGFR-TKIs may predict cross-resistance to future generations, highlighting the need for alternative therapeutic strategies after resistance develops.

To identify key EGFR-independent mechanisms of resistance, we performed global LC-MS/MS proteomic analysis on our cell lines, followed by pathway enrichment analysis of DEPs shared within resistant lines. Gefitinib-resistant cells showed fewer overlapping DEPs compared to those observed in dacomitinib- and osimertinib-resistant cells, which may be partly caused by the different origin of parental cells (C827 vs. H1975). Pathways regulating cell survival and metastasis were identified as universal factors in EGFR-TKI resistance across drug generations. Network analysis revealed a signaling hub that impacts cellular ECM, invasion and survival, which may be crucial for the development of EGFR-TKI resistance, especially in the advanced generations.

In contrast to conventional resistance-observational studies, we conducted drug combination experiments to validate whether proteins/pathways, highlighted in our proteomic analysis, can mediate the development of EGFR-TKI resistance. We started from apoptosis-related proteins; their dysregulation is profoundly associated with carcinogenesis and the development of drug resistance [56]. Our data demonstrated that targeting BCL-2 could be a potential shared resistance remedy across advanced drug generations, but not for the first one in generated models. Evidently, the upregulation of this anti-apoptotic protein does not have sufficient power to compete with other mechanisms and guide the resistance behavior in C827/GEF cells. Interestingly, BCL-2 has recently been proposed as a common EGFR-TKI resistance mechanism/target [57–59]; however, our results show that it cannot be considered truly universal. At the same time, they bring an important warning that correlation expression studies cannot reliably judge the true role of putative resistance mechanisms without functional validation.

CSC-related pathways (Hedgehog and Notch pathways) were also suspected as possible shared resistance drivers in our proteomic analysis. They have recently become innovative targets in clinical oncology, while their functions have been verified to be tightly associated with the activities of ECM [40]. We previously introduced sonidegib, a marketed SMO inhibitor, as an effective modulator counteracting ABCB1/ABCG2-mediated chemotherapeutic resistance in NSCLC [31]. In the current work, we demonstrated that sonidegib might have additional resistance-modulatory value, synergistically combatting dacomitinib and osimertinib resistance.

Then, we investigated pharmacokinetic contributions to EGFR-TKI resistance, identifying ABCG2 overexpression in all three resistant cell lines. It is well-known that drug substrates become multidrug resistance (MDR) victims through the upregulation of the respective ABC transporter [60]. As gefitinib, dacomitinib, and osimertinib are ABCG2 substrates, observed upregulation aligns mechanistically with this rule. In our drug combinations, functional ABCG2 inhibition via Ko143 synergistically restored sensitivity to dacomitinib and osimertinib. In fact, several TKIs (e.g., sonidegib, tepotinib, and ensartinib) have been previously shown to inhibit ABCG2 and thus antagonize cytostatic resistance [31, 38, 61]. Substituting Ko143 with these TKIs may offer clinically feasible combination strategies to overcome resistance to advanced generation of EGFR-TKIs. Based on the outcomes of another study [62], ABCG2 might seem to act as a universal EGFR-TKI resistance driver. Nevertheless, similar to BCL-2, a discrepancy between observed ABCG2 upregulation and lack of synergy in drug combinations has been observed in C827/GEF cells, thus refuting this claim. Additionally, this repeated non-correlation with the pattern recorded in H1975 models suggests that the cellular/genetic background is likely a more important factor in shaping the resistance phenotype than the drug’s generation.

Our proteomic analysis revealed dysregulated expression of ECM-associated proteins in the resistant model, suggesting their regulatory involvement in the development of EGFR-TKI resistance. In the follow-up study, we found that FAK and YAP1 signaling pathways are universally upregulated in drug-resistant cells. Both pathways participate in cancer proliferation, invasion, and therapy resistance through their interactions with ECM [63, 64]. Targeting them with specific inhibitors (defactinib and verteporfin, respectively) in combination resistance-reversal assays resulted in synergistic outcomes across all three drug-resistant models, demonstrating their impacts on EGFR-TKI resistance and potentials as therapeutic targets.

To further investigate the relationship between ECM and EGFR-TKI resistance, we cultured cells as 3D-structured spheroids. Our findings showed that the acquisition of resistance to dacomitinib and osimertinib enhanced cellular invasion and migration in collagen matrices, whereas the development of resistance to gefitinib caused fewer morphological changes. The increased invasive activities may be due to the downregulation of EGFR expression in drug-resistant cells, which is aligned with the finding that EGFR inhibition can induce the cellular invasion in 3D environments. Further mechanistic studies showed that epithelial-mesenchymal transition (EMT) can promote the development of EGFR-TKI resistance as well as the increased invasiveness, after EGFR inhibitions [65, 66]. In our proteomic results, gefitinib-resistant cell line did not exhibit the pronounced alterations of EMT-related proteins as the other two resistant variants, which could be the reason for its less morphological changes.

Defactinib monotreatment significantly inhibited NSCLC cell proliferation in spheroid models and suppressed the invasive potential of dacomitinib- and osimertinib-resistant cells, though not in gefitinib-resistant cells. This may be attributed to the unchanged phosphorylated Erk 1/2 levels in C827/GEF cells, a key regulator of invasion in FAK signaling. Notably, defactinib demonstrated anti-NSCLC activity with a favorable tolerability profile in a phase II trial and has received FDA breakthrough therapy designation in combination with VS-6766 for ovarian cancer [67, 68]. These findings highlight FAK inhibition as a potential strategy to overcome EGFR-TKI resistance by simultaneously targeting proliferation and metastasis.

Conversely, verteporfin-mediated YAP1 inhibition reduced invasion in dacomitinib-resistant spheroids but failed to suppress proliferation. Prior studies suggest verteporfin inhibits invasion dose-dependently [69], yet its efficacy in our 3D model may have been limited by the 0.05 µM concentration used, which was effective in two-dimensional (2D) but potentially insufficient in 3D. Despite this, verteporfin has shown promise in preclinical models, demonstrating synergy with EGFR-TKIs in 2D cultures [70]. As YAP1-TEAD signaling regulates proliferation, metastasis, and drug resistance, clinical validation is essential to determine its role in overcoming EGFR-TKI resistance in NSCLC.

Last but not least, our proteomic and western blotting data also suggested that ECM-related proteins and their interaction networks might be particularly crucial in T790M-harbored NSCLC cells after generating drug resistance. Among them, FN1 regulates ECM assembly and interacts extensively with ECM components, playing a crucial role in the communication between the intracellular and extracellular environments [71]. After silencing FN1, drug-resistant cells exhibited similar or greater sensitivities to EGFR-TKIs, compared to the parental cells. We further explore the roles of ECM members in NSCLC development using public dataset (GSE31210) [39]. Aligned with our proteomic data, expression of FN1 and its interactive members was found to be associated with NSCLC poor prognosis and relapse following EGFR-TKI therapy. ECM and its related signaling critically shape cancer biology by driving proliferation, invasion, and therapy resistance. As aforementioned, anti-apoptotic protein (BCL-2), CSC-related signaling (Hedgehog and Notch pathways) and drug efflux transporter (ABCG2) can co-participate in developing EGFR-TKI resistance. Previous studies have clarified that ECM remodeling enhances tumor cell survival by activating downstream, such as integrin/FAK and YAP/TAZ pathways, which suppress apoptosis and promote CSC traits [72]. Besides, ECM stiffness can also upregulate the expression and activity of ABC transporters, enabling drug efflux to induce resistance [73]. In response to our work, we believe that ECM-related signaling plays a central hub role in the regulation of acquired EGFR-TKI resistance.

In summary, we developed and characterized acquired EGFR-TKI-resistant cells in a unique transitional state, which was driven by the signaling pathways alterations, prior to the emergence of secondary EGFR mutations. While high consistency in overlapping expression changes has been seen in advanced generations, outcomes in first generation gefitinib (model derived from different parental cells) varied substantially. Our comparative study with multiple drug generations was conducted by a single team with identical methodologies, which is unique among the studies from the field. Due to this feature, our data show that shared mechanisms are more likely to occur within similar cells, independently of drug generation, suggesting that genetic/cellular background is a major factor affecting the development of resistance mechanisms. In contrast to other conventional transcriptomic/proteomic studies from the field, we functionally validated the promising protein candidates as potential therapeutic targets, bringing new translational results. Similar to expression experiments, synergistic drug combinations showed high consistency for advanced EGFR-TKI generations, whereas validation failed for BCL-2 and ABCG2 in gefitinib-resistant cells. This observation further underlines the power of genetic/cellular background in shaping drug-resistant phenotypes and concomitantly suggests another important message: despite the expression upregulation, particular protein/pathway must not necessarily have a decisive value as resistance driver. Cancer drug resistance is intrinsically highly multifactorial phenomenon [74], which correlates with this finding. Importantly, as a novel finding, we demonstrated that only ECM-related (FAK and YAP1) pathways mediate EGFR-TKI resistance and act as shared potential targets independently on drug generation and genetic background of parental cells. Based on patient-based bioinformatic analysis, other ECM-related members anchored by FN1 are also associated with the malignant progression of NSCLC and poor treatment outcomes. Collectively, these results underline the role of ECM signaling as key mechanism of EGFR-TKI resistance with potential therapeutic value.

Finally, we are aware that our work has several limitations, which it is appropriate to acknowledge and discuss. First, the use of two genetically distinct cell models limits our ability to determine whether the observed similarities and differences between resistant models arise from the cellular background or from drug generation–specific resistance mechanisms. However, the use of a single model would not represent a suitable alternative, considering the pharmacological irrationality of applying models with baseline mutations to higher drug generations and vice versa. The approach of utilizing a single cell line to filter out background effects carries a high risk of identifying false-positive shared mechanisms that may not occur in the human body, as clearly suggested by our data. We therefore believe that only a design reflecting pharmacological and clinical reality can yield translatable results with potential benefit for scientists and mainly oncological patients. Importantly, the perceived “disadvantage” of using two cell lines has provided a valuable demonstration of how critical the genetic and cellular background is for the development of resistance mechanisms. At the same time, it indicates that within a similar background, shared mechanisms are much more likely to occur, independently of drug generation. Notably, owing to this study design, we identified ECM signaling as a truly shared and targetable EGFR-TKI resistance mechanism, independent of cellular background. It is true that ECM signaling, along with few other pathways, has already been reported as a potential EGFR-TKI resistance mechanism in previous studies, which may be considered another limitation. The central aim of our work is to identify shared resistance mechanisms that could potentially enable “one-hit-for-all” therapeutic strategies. In this sense, our study, which aims to address this goal within a unified framework, has an integrative character, spanning more than 25 years of EGFR-TKI clinical history and thus conceptually resembling a review article. Some degree of overlap with existing knowledge is therefore expected. Importantly, in this context, previously described mechanisms should not be interpreted as a lack of originality, but rather as supportive evidence reinforcing their generality across drug generations and experimental models (particularly given that these findings originate from independent teams, drugs, models, and conditions). This may, in effect, substitute for extensive multi-model validation and provide stronger support for truly shared resistance mechanisms. From our perspective, the final major limitation is the absence of direct in vivo experiments as a means of validating the observed findings. Considering the comprehensive scope and already broad range of our study, we propose that in vivo validation should be addressed in a dedicated follow-up investigation. We believe that our bioinformatic analysis of samples derived from human oncological patients provides an appropriate form of pre–in vivo validation of our conclusions.

## Conclusions

Our functional and bioinformatic data highlighted ECM-related signaling as a central driver of EGFR-TKI resistance for all generations, irrespective of the cellular background. Next, we demonstrated that advanced-generation EGFR-TKIs share pharmacodynamic (BCL-2 protein and CSC pathways) and pharmacokinetic (ABCG2 transporter) resistance mechanisms when derived from the same cellular model. The synergistic efficacy of combining EGFR-TKIs with inhibitors targeting these pathways supports dose-reduction strategies to minimize toxicity without compromising therapeutic activity. Future validations in animal models and primary patient-derived models will be important to confirm these findings and refine translatable approaches. Targeting ECM-associated signaling, in particular, may offer a promising strategy to overcome resistance, benefiting EGFR-TKI-resistant NSCLC patients.

## Supplementary Information

Supplementary Material 1. Supplementary Table S1: Information for primary antibodies. Supplementary Figure 1: EGFR mutational status for the C827 and H1975 parental and resistant cell lines. Supplementary Figure 2: Assessment of expression of apoptosis-related genes and drug combinations in C827 parental and GEF-resistant cells. Supplementary Figure 3: FN1 knockdown experiments in H1975/DAC and H1975/OSI. Supplementary Figure 4: Kaplan-Meier overall survival curves for NSCLC patients with EGFR mutations, stratified by relapse status

Supplementary Material 2. Original images from Western blots

## Data Availability

The authors declare that the data generated and analyzed during this study are included in this published article. In addition, datasets generated and/or analyzed during the current study are available from the corresponding author upon reasonable request. Re-usable data from global proteomic analysis were loaded into Zenodo repository (https://doi.org/10.5281/zenodo.20018096).

## Abbreviations

2D: Two dimensional
3D: Three-dimensional
95% CI: 95% Confidence interval
AA: Amino acid
ABC: ATP-binding cassette
ACN: Acetonitrile
AFA: Afatinib
AGC: Automated gain control
C827: HCC827
C827/GEF: Gefitinib-resistant C827
CI: Combination index
CSCs: Cancer stem cells
DAC: Dacomitinib
ddPCR: Droplet digital PCR
DEF: Defactinib
DEPs: Differentially expressed proteins
DMSO: Dimethyl sulfoxide
dNTP: Deoxynucleotide
ECM: Extracellular matrix
EDTA: Ethylenediamine tetraacetic acid
EGFR: Epidermal growth factor receptor
EGFR-TKIs: EGFR-tyrosine kinase inhibitors
EGTA: Ethylene glycol-bis(β-aminoethyl ether)-N, N,N′,N′-tetraacetic acid
EMT: Epithelial-mesenchymal transition
ERL: Erlotinib
F_A_: Fractions of cells affected
FA: Formic acid
FBS: Fetal bovine serum
FWHM: Full width at half maximum
GEF: Gefitinib
GEO: Gene Expression Omnibus
H1975: NCI-H1975
H1975/DAC: Dacomitinib-resistant H1975
H1975/OSI: Osimertinib-resistant H1975
HEPES: 4-(2-hydroxyethyl)-1-piperazineethanesulfonic acid
HRP: Horseradish peroxidase
IC_50_: Half-maximal inhibitory concentration
LC-MS/MS: Liquid chromatography/tandem mass spectrometry
MDR: Multidrug resistance
MEM: Minimum Essential Medium
MTT: 3-(4,5-dimethylthiazol-2-yl)-2,5-diphenyltetrazolium bromide
NC: Negative control
NSCLC: Non-small cell lung cancer
OLM: Olmutinib
OS: Overall survival
OSI: Osimertinib
par: Parental
PBS: Phosphate buffered saline
PFS: Progression-free survival
PVDF: Polyvinylidene difluoride
RO: RO4929097
RPMI-1640: Roswell Park Memorial Institute medium 1640
R_R_: Reversal ratio
SDC: Sodium deoxycholate
SDS-PAGE: Sodium dodecyl sulfate polyacrylamide gel electrophoresis
siRNA-FN1: Small interfering RNA targeting human fibronectin
siRNA-NC: Silencer™ Select Negative Control
SON: Sonidegib
TBST: Tris-buffered saline with 0.1% Tween 20 detergent
TEAB: Triethylammonium bicarbonate
TFA: Pierce™ Trifluoroacetic Acid
TME: Tumor microenvironment
VEN: Venetoclax
VER: Verteporfin
