# Supplementary material for: Unmasking the common enemy: drug resistance mechanisms across three different EGFR inhibitor generations are associated with co-targetable alterations in extracellular matrix signaling
Source: Cell Commun Signal. 2026 May 6;24:376. doi: 10.1186/s12964-026-02927-8 (PMC13312578; doi:10.1186/s12964-026-02927-8)
Supplement: Supplementary file 1 — Supplementary Material 1. Supplementary Table S1: Information for primary antibodies. Supplementary Figure 1: EGFR mutational status for the C827 and H1975 parental and resistant cell lines. Supplementary Figure 2: Assessment of expression of apoptosis-related genes and drug combinations in C827 parental and GEF-resistant cells. Supplementary Figure 3: FN1 knockdown experiments in H1975/DAC and H1975/OSI. Supplementary Figure 4: Kaplan-Meier overall survival curves for NSCLC patients with EGFR mutations, stratified by relapse status [file 12964_2026_2927_MOESM1_ESM.docx]

**Supplementary Table S1**: Information for primary antibodies.

| **Suppliers** | **Target protein**  **(Abbreviation)** | **Cat. no.** | **Working concentration**  **(In 1 × TBST solution)** |
| --- | --- | --- | --- |
| Santa Cruz |  |  |  |
|  | ABCB1 | sc-13131 | 1:200 |
|  | ABCC1 | sc-18835 | 1:250 |
|  | ABCG2 | sc-377176 | 1:2000 |
|  | BCL-2 | sc-7382 | 1:1000 |
|  | GLI1 | sc-515751 | 1:1000 |
|  | GLI2 | sc-271786 | 1:1000 |
|  | SMO | sc-166685 | 1:500 |
|  | Phosphorylated FAK (p-FAK) | sc-81493 | 1:2000 |
|  | FAK | sc-271126 | 1:1000 |
|  | Phosphorylated Erk 1/2  (p-Erk 1/2) | sc-81492 | 1:1000 |
|  | Erk 1/2 | sc-514302 | 1:1000 |
|  | Fibronectin (FN1) | sc-8422 | 1:1000 |
|  | Integrin β6 (ITGB6) | sc-517598 | 1:1000 |
|  | CD44 | sc-7297 | 1:1000 |
|  | PLOD1 | sc-271640 | 1:1000 |
|  | MMP14 | sc-377097 | 1:1000 |
|  | VIMENTIN | sc-6260 | 1:1000 |
|  | Ep-CAM | sc-25308 | 1:1000 |
| Abcam |  |  |  |
|  | Phosphorylated YAP1 (p-YAP1) | ab76252 | 1:10000 |
|  | β-actin | ab8226 | 1:10000 |
| Cell Signaling Technology |  |  |  |
|  | NOTCH-1 | 3608s | 1:1000 |
|  | cleaved NOTCH-1 | 4147s | 1:1000 |
|  | RBPSUH (CSL)  EGFR | 5313s  2239t | 1:1000  1:1000 |
| Abclonal |  |  |  |
|  | YAP1 | A19134 | 1:1000 |


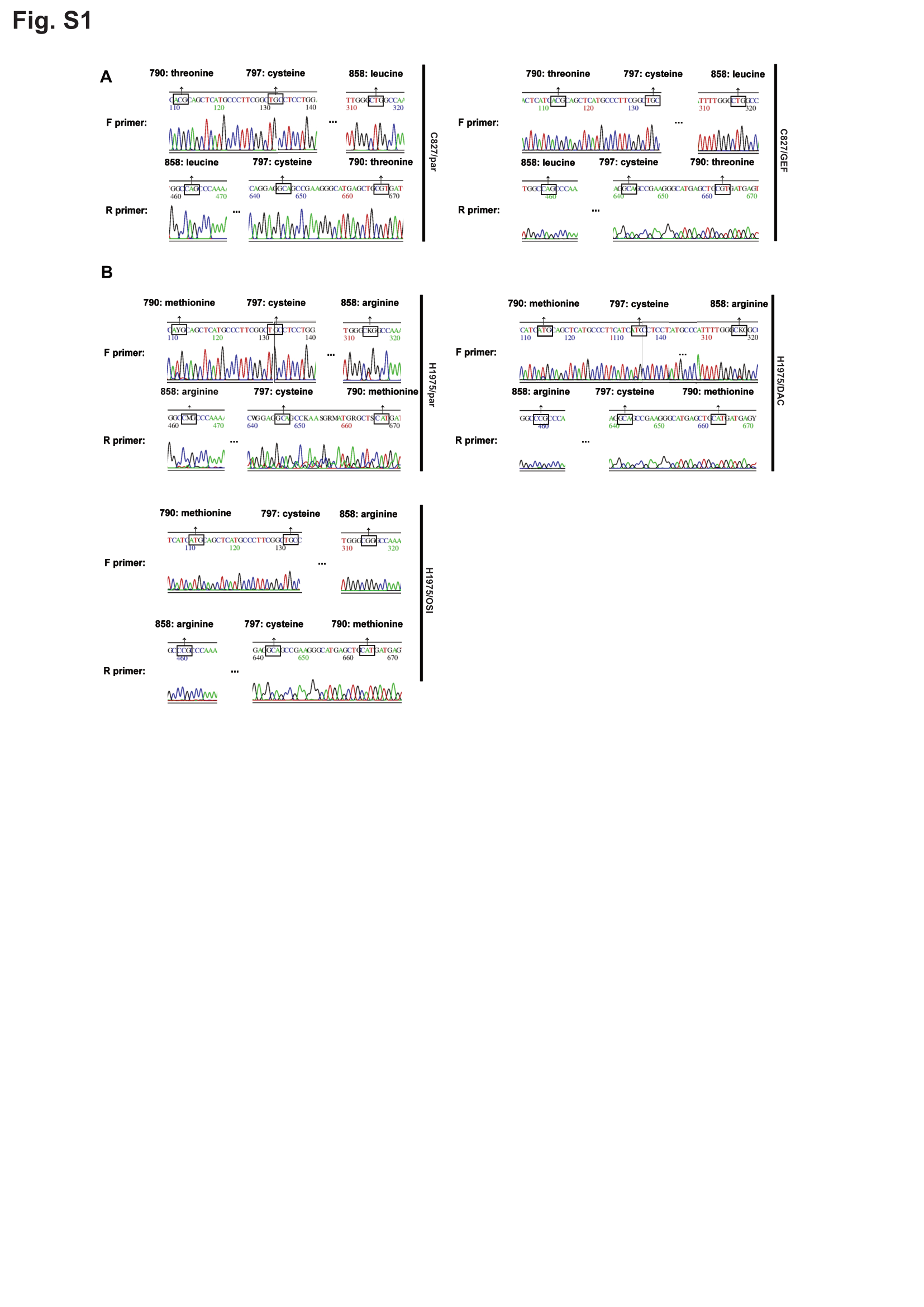


**Supplementary Figure 1**: *EGFR* mutational status for the C827 and H1975 parental and resistant cell lines. (A) Sequencing chromatograms showing the AA sequences at positions 790, 797, and 858 in the EGFR gene for the C827 parental cell lines (C827/par) and gefitinib-resistant cells (C827/GEF). The chromatograms confirm the wild-type sequences in C827 cells, with no additional change after acquiring resistance. (B) Sequencing chromatograms demonstrating *EGFR* mutational status in parental H1975 cells and their dacomitinib- (H1975/DAC) and osimertinib-resistant (H1975/OSI) subvariants. Presence of common activating L858R mutation and first generation TKI resistance-related T790M mutation has been confirmed in H1975 parental model, with no additional change after acquiring dacomitinib resistance. Osimertinib resistant cells lost the heterozygosity at amino-acid positions 790 and 858, with no appearance of C797S mutation.


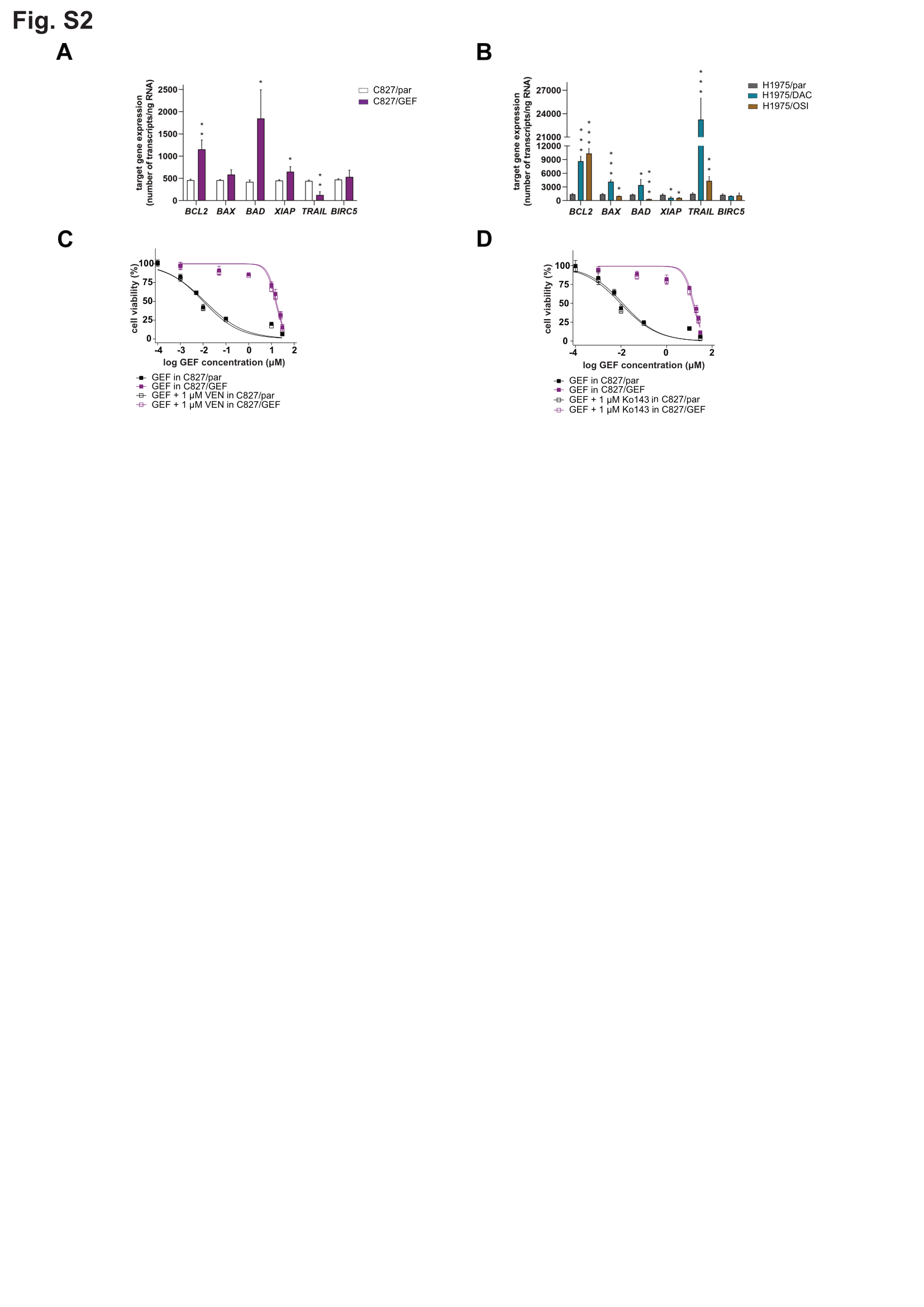


**Supplementary Figure 2:** Assessment of expression of apoptosis-related genes and drug combinations in C827 parental and GEF-resistant cells.

(A, B) RNA expression levels of apoptosis-related genes (*BCL2*, *BAX*, *BAD*, *XIAP*, *TRAIL*, and *BIRC5*) in parental (C827/par, H1975/par) and resistant (C827/GEF, H1975/DAC, and H1975/OSI) cell lines. Data are presented as mean ± SD of the number of transcripts/ng RNA, with significant differences relative to parental lines indicated.

(C, D) Dose-response curves showing cell viability with increasing concentrations of gefitinib in C827/par and C827/GEF cell lines. The addition of 1 µM venetoclax (VEN) or Ko143 failed to significantly enhance the sensitivity of resistant cell line to gefitinib.


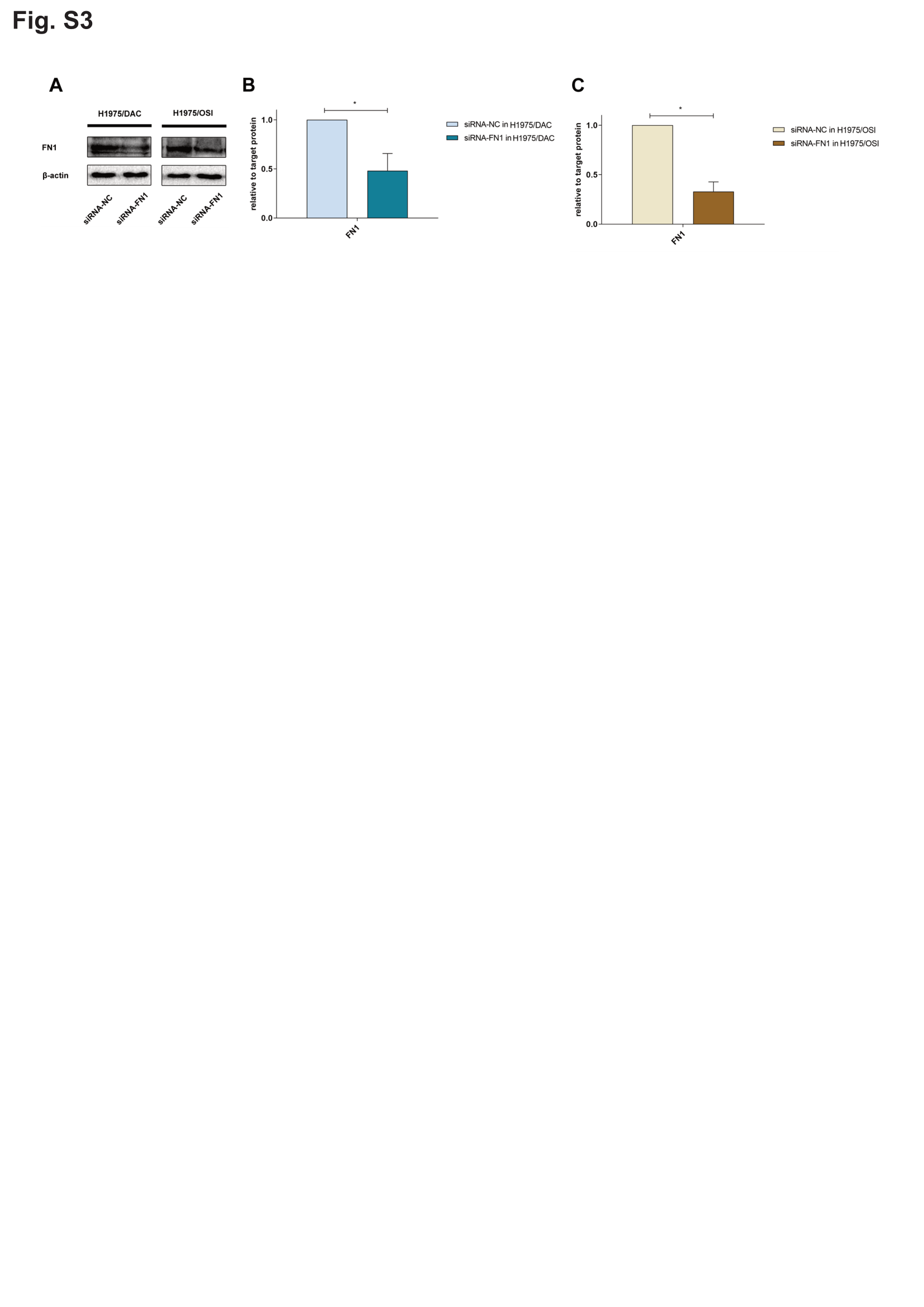


**Supplementary Figure 3:** FN1 knockdown experiments in H1975/DAC and H1975/OSI. Tested cells were transfected with 25 nM siRNA-FN1 or siRNA-NC for 24h. Western blotting assay was performed for determining the gene knockdown effects of siRNA. (A) Representative images from western blotting. (B, C) Quantitative analysis for the band intensities in H1975/DAC and H1975/OSI.


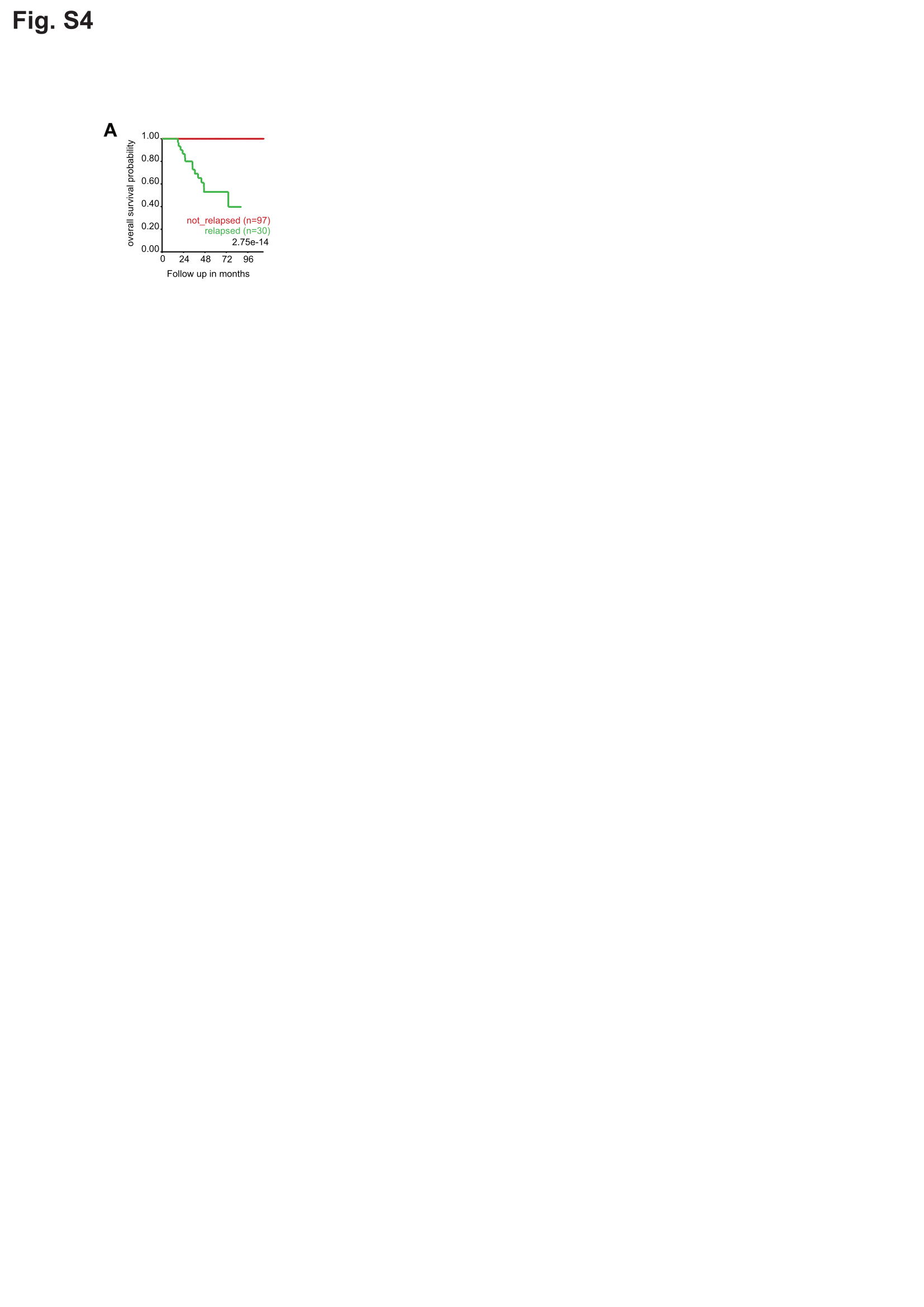


**Supplementary Figure 4**: Kaplan-Meier overall survival curves for NSCLC patients with EGFR mutations, stratified by relapse status. Patients, who did not relapse (red line, n = 97), exhibited significantly better overall survival compared to those, who relapsed (green line, n = 30). Statistical significance was determined using a log-rank test (p = 2.75 × 10⁻¹⁴).
