# Supplementary material for: Unmasking the common enemy: drug resistance mechanisms across three different EGFR inhibitor generations are associated with co-targetable alterations in extracellular matrix signaling
Source: Cell Commun Signal. 2026 May 6;24:376. doi: 10.1186/s12964-026-02927-8 (PMC13312578; doi:10.1186/s12964-026-02927-8)
Supplement: Supplementary file 2 — Supplementary Material 2. Original images from Western blots [file 12964_2026_2927_MOESM2_ESM.docx]

Fig. 1H

EGFR


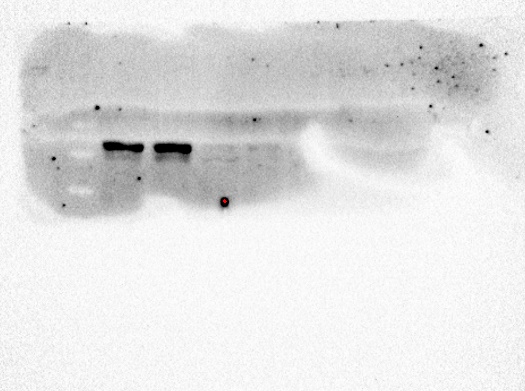

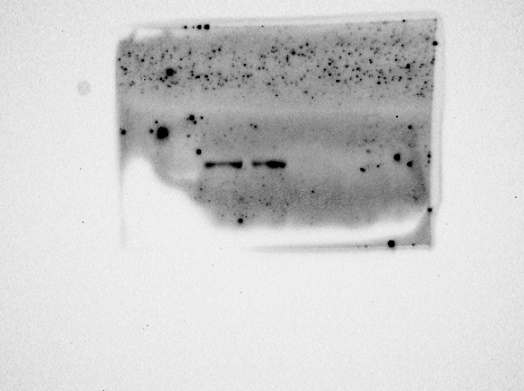


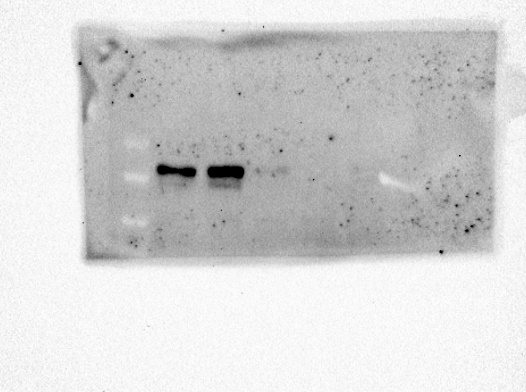


β-actin


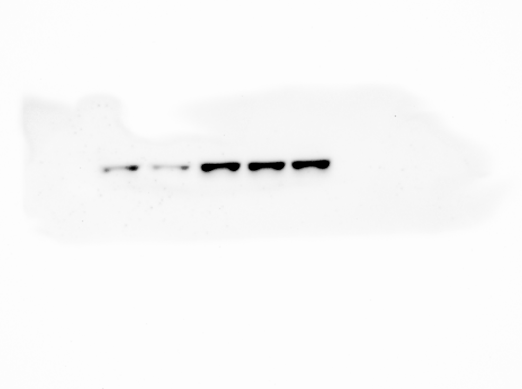

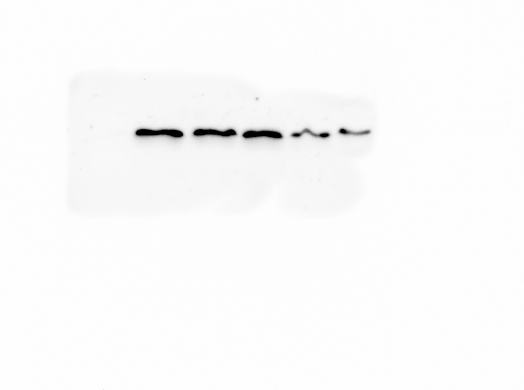

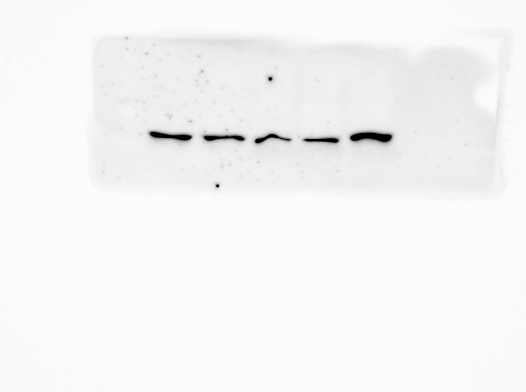


Fig. 3A

BCL-2


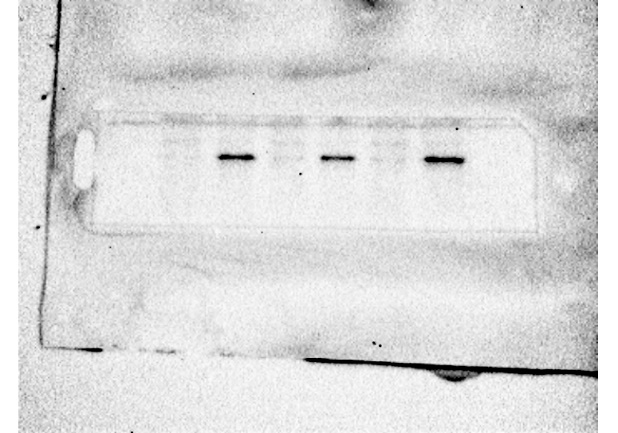

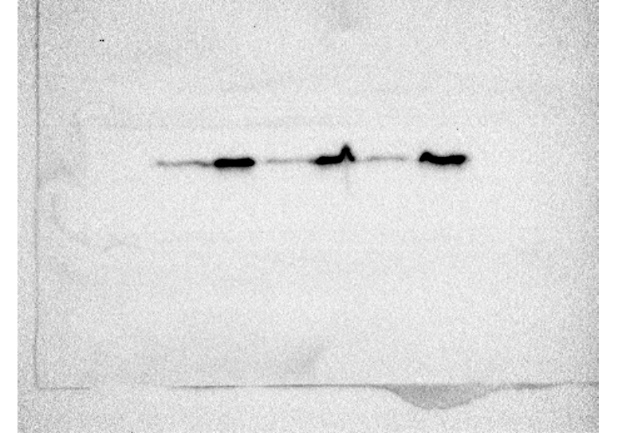


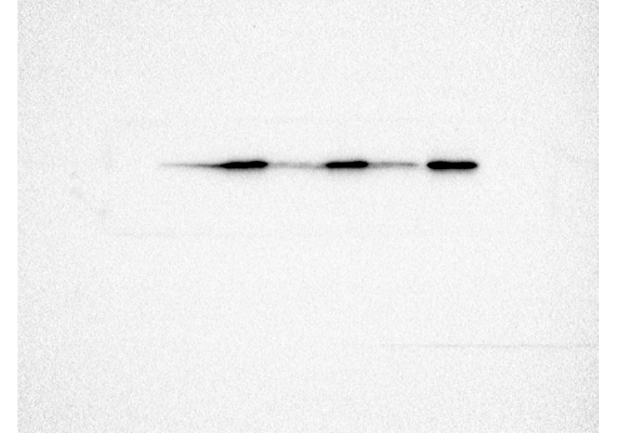


β-actin


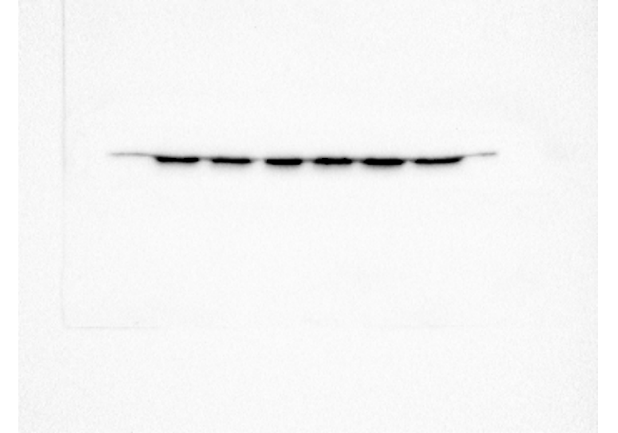

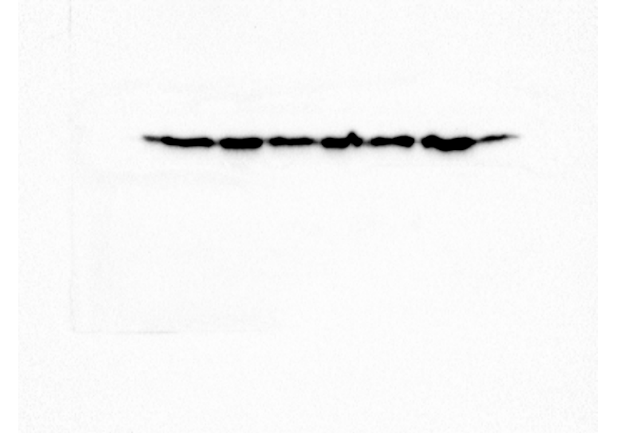


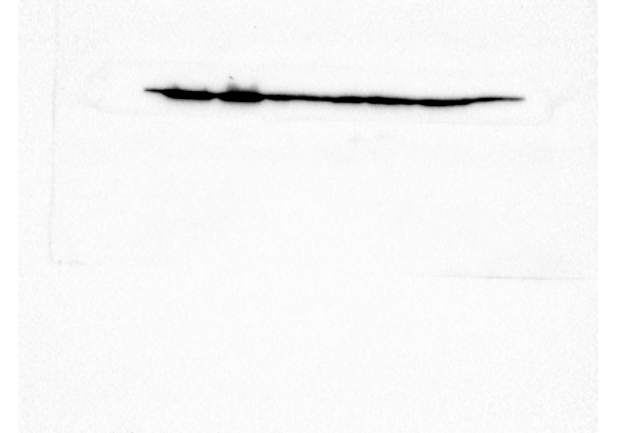


Fig. 3E

GLI1


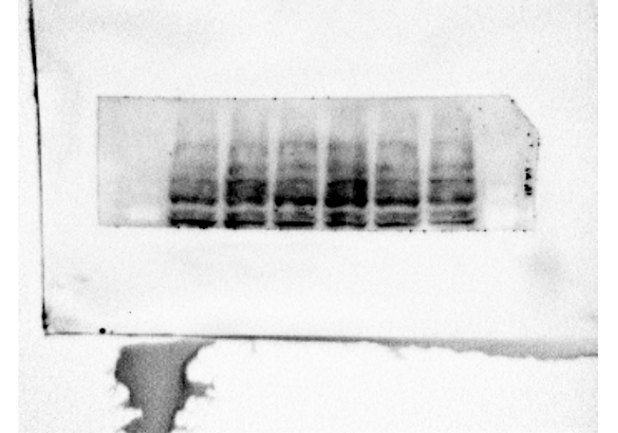

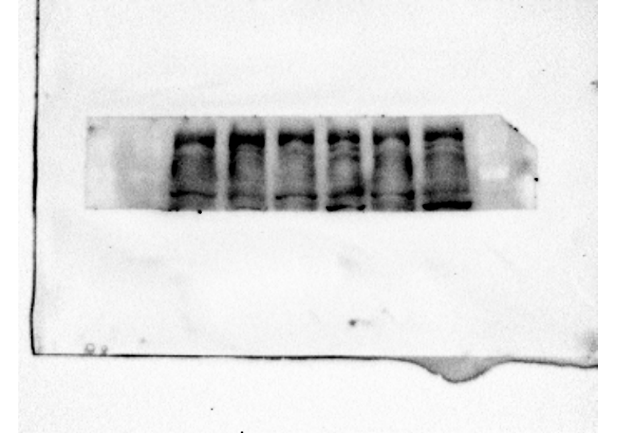


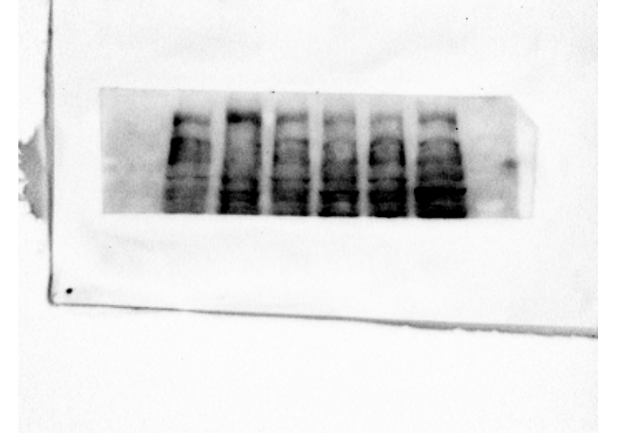


GLI2


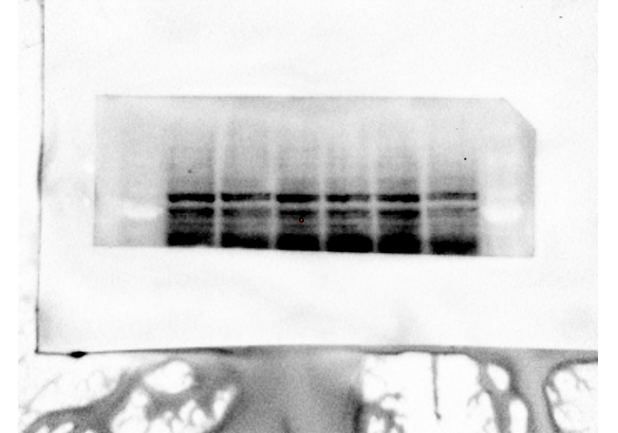

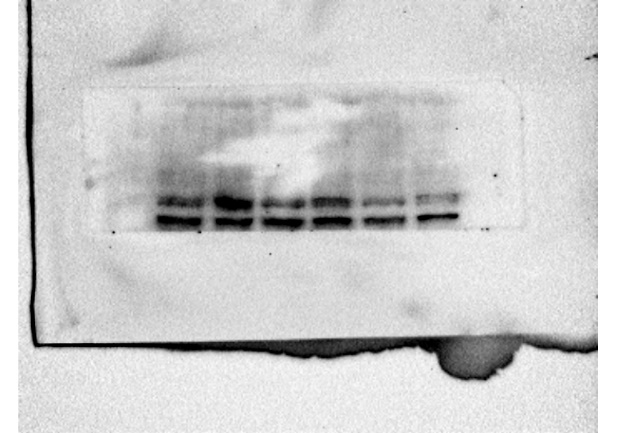


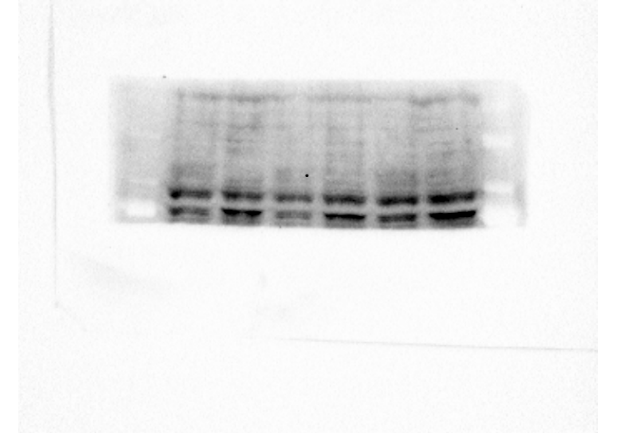


SMO


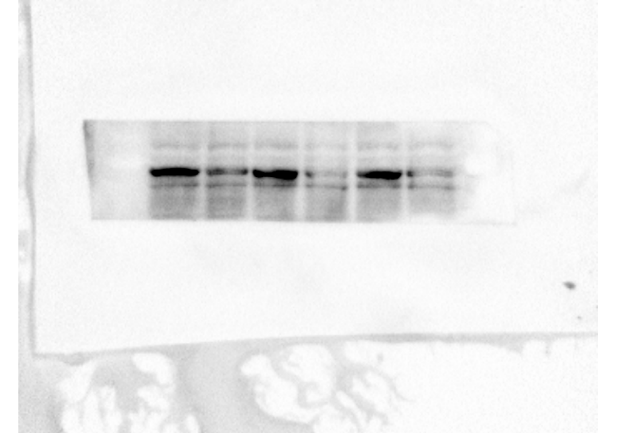

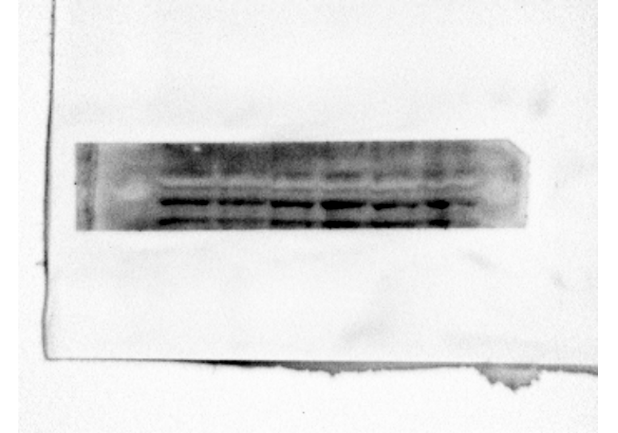


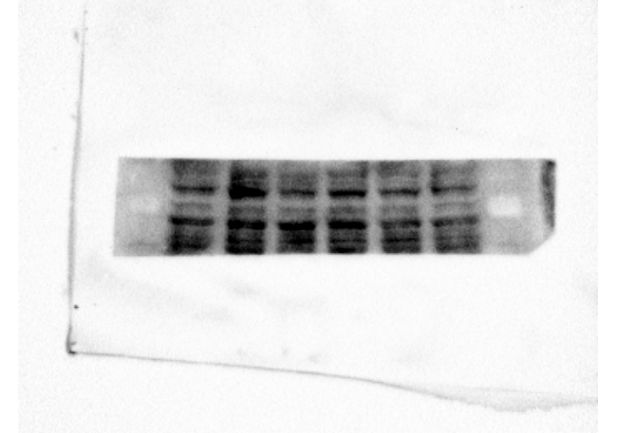


β-actin


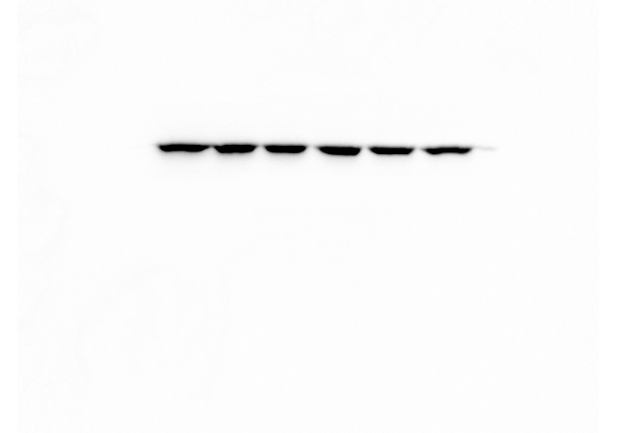

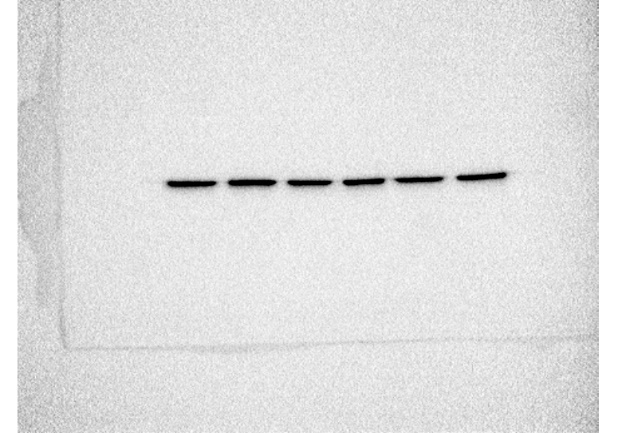


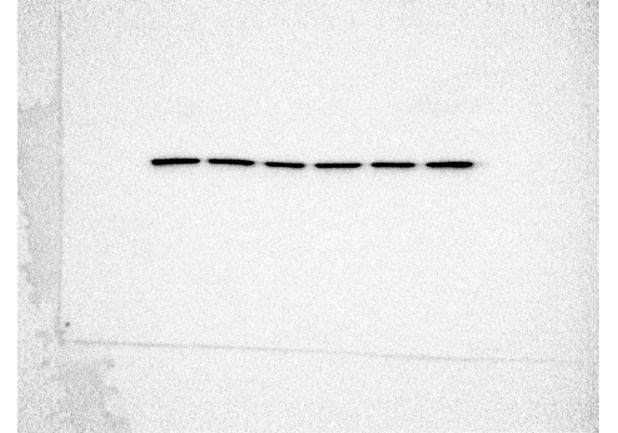


Fig. 3I

NOTCH-1


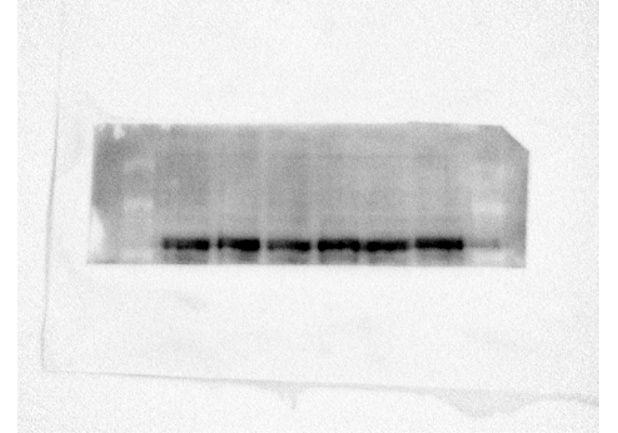

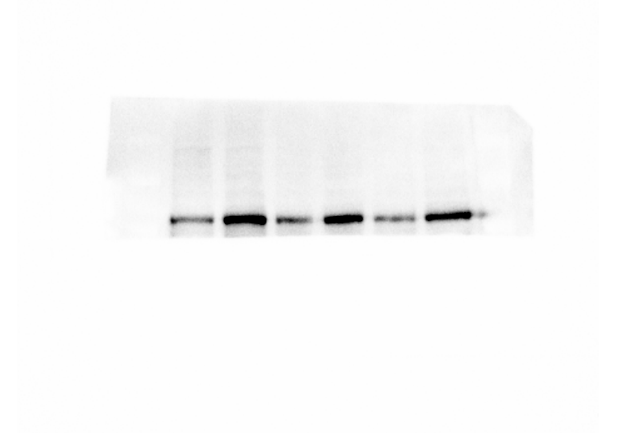


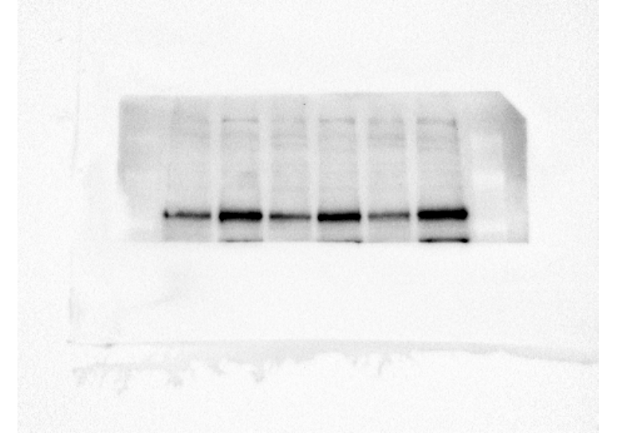


cleaved-NOTCH-1


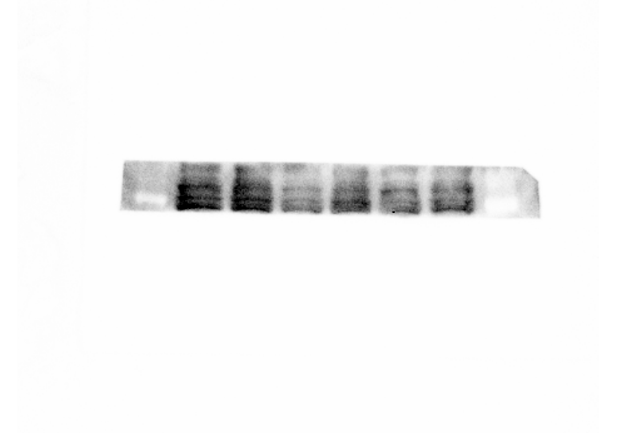

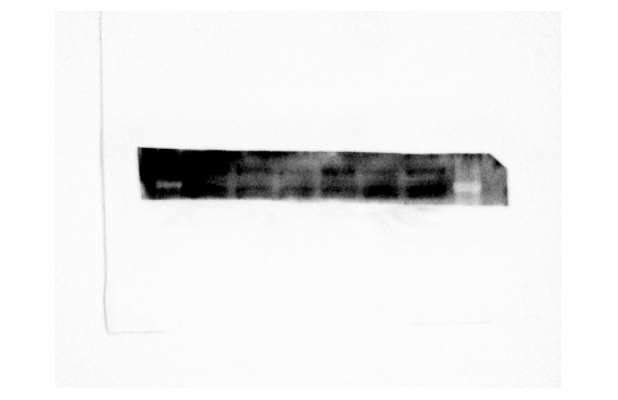


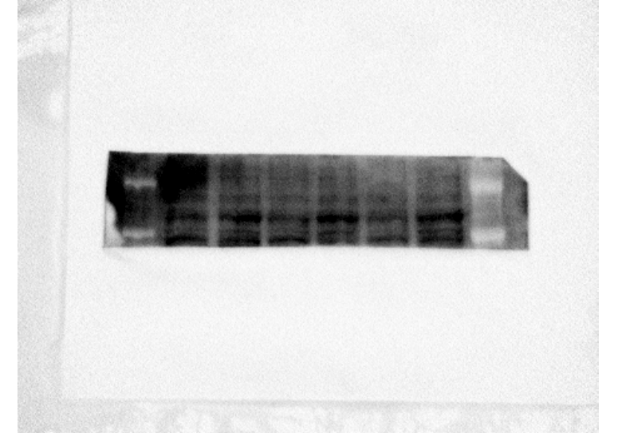


CSL


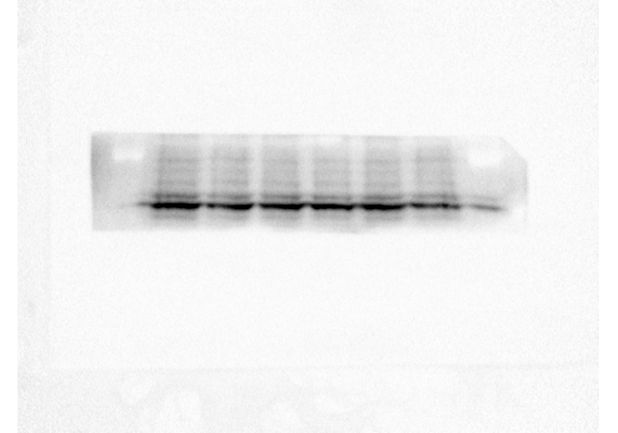

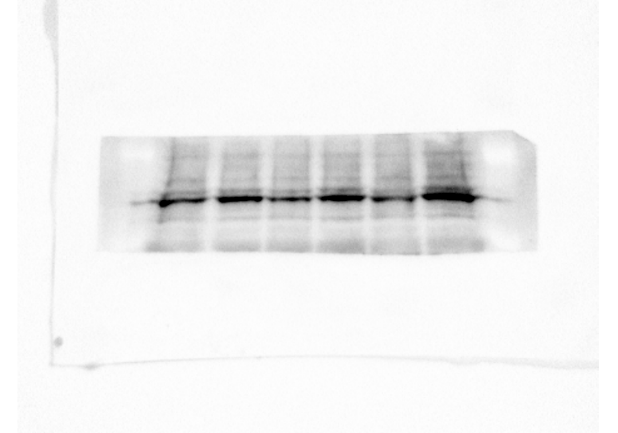


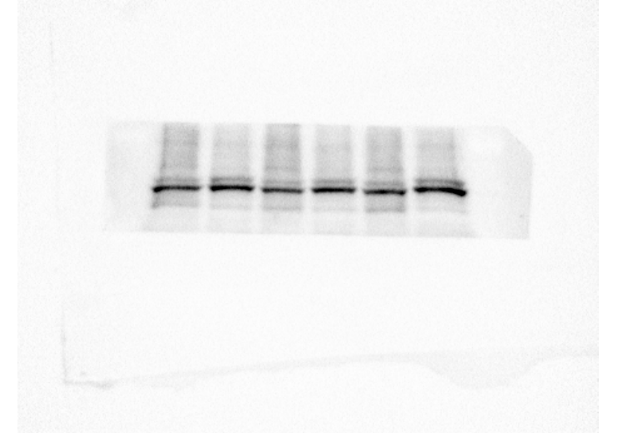


β-actin


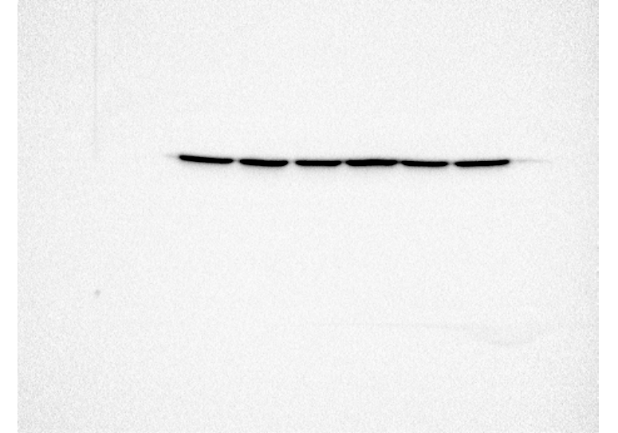

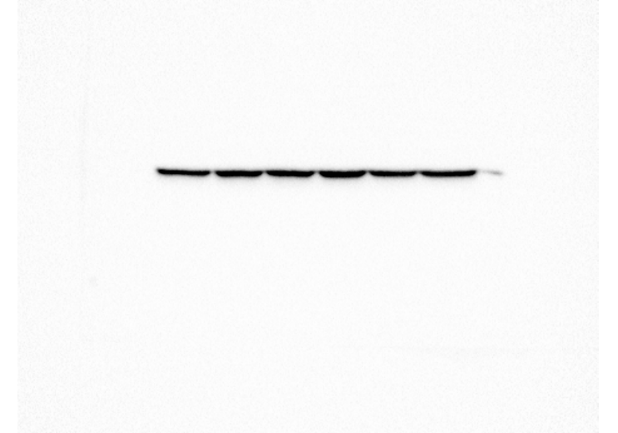


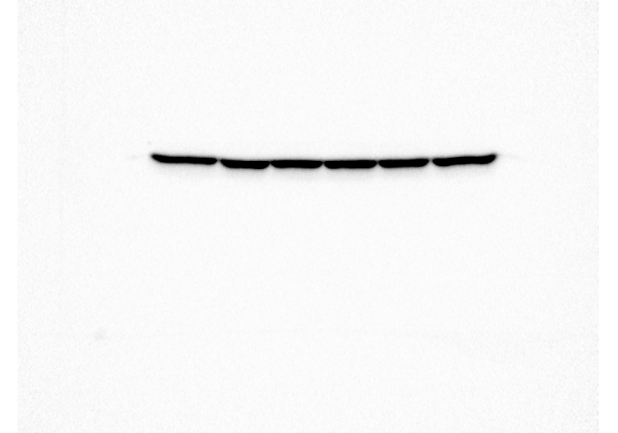


Fig. 3M

ABCB1


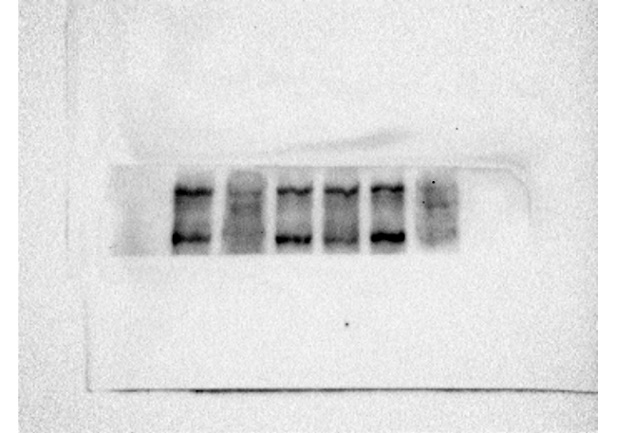

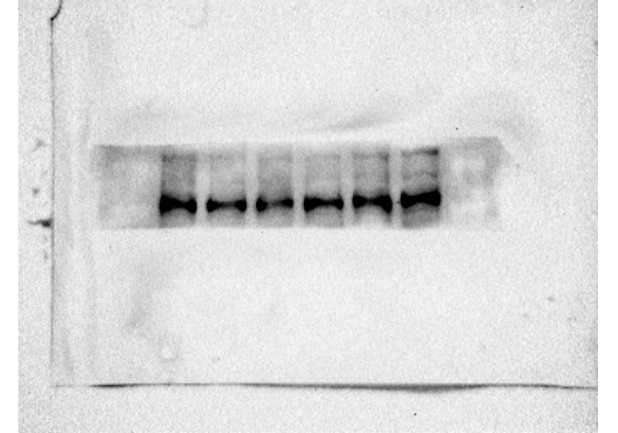


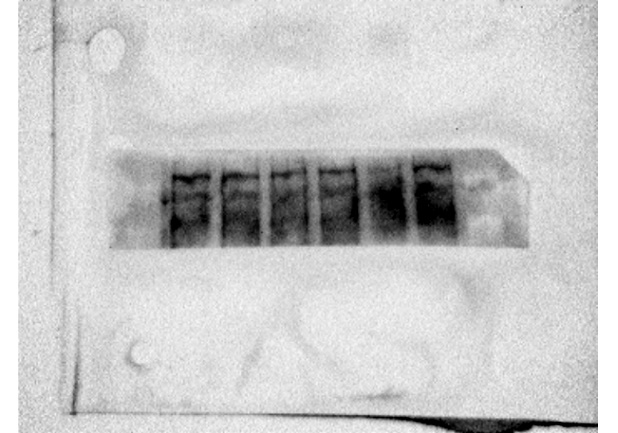


ABCC1


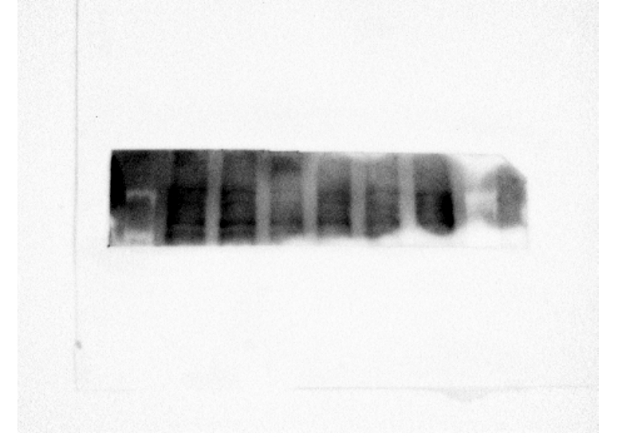

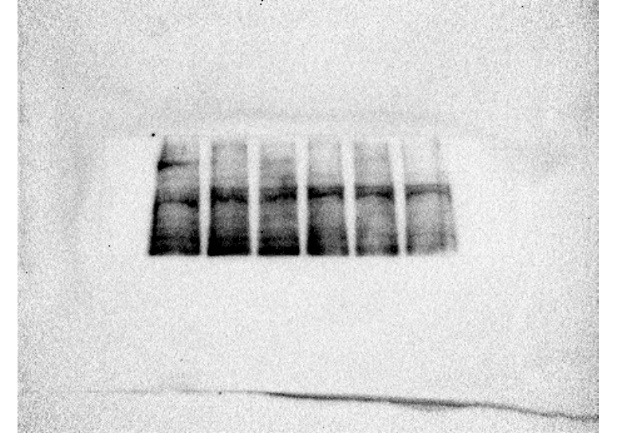


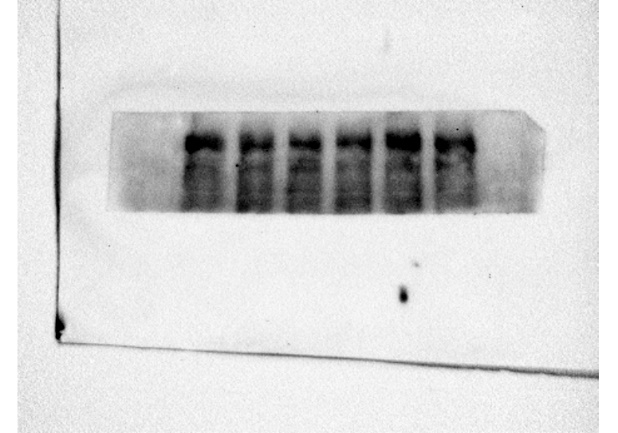


ABCG2


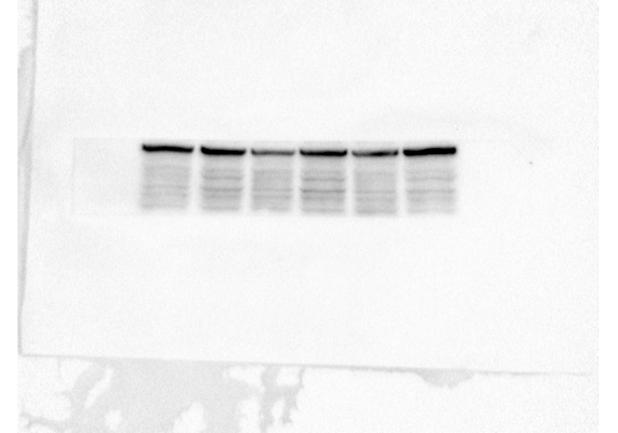

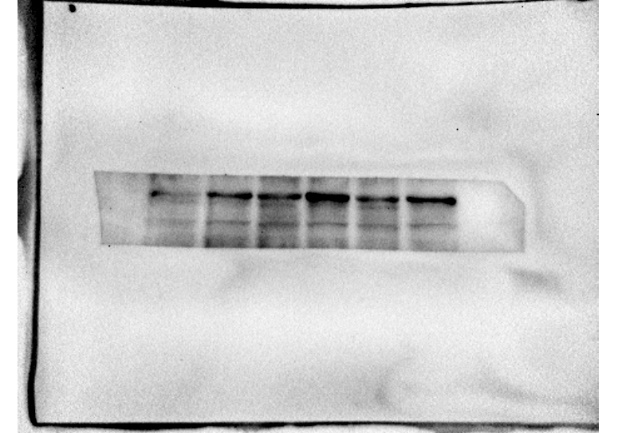


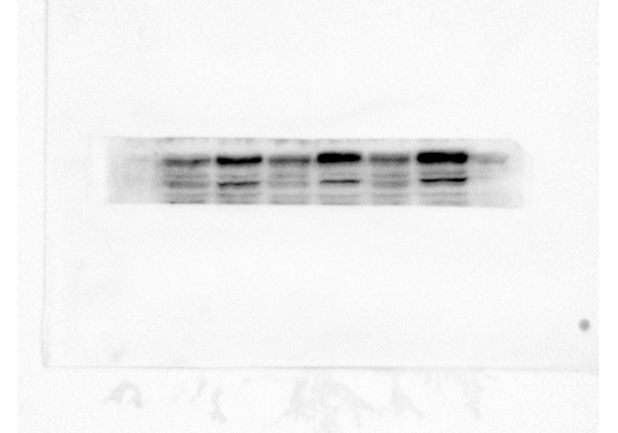


β-actin


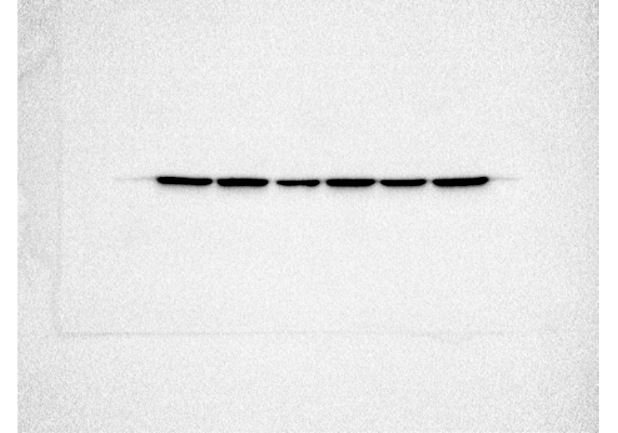

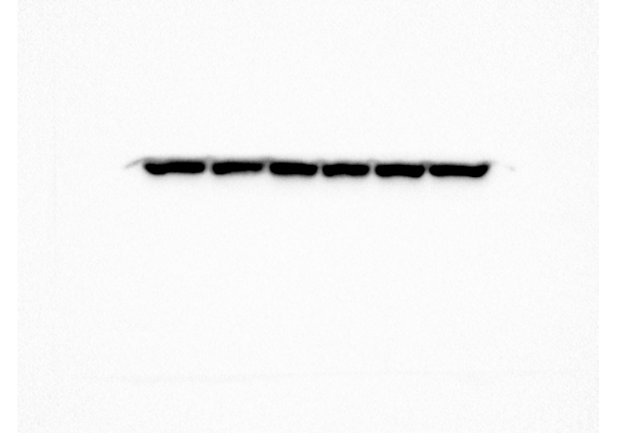


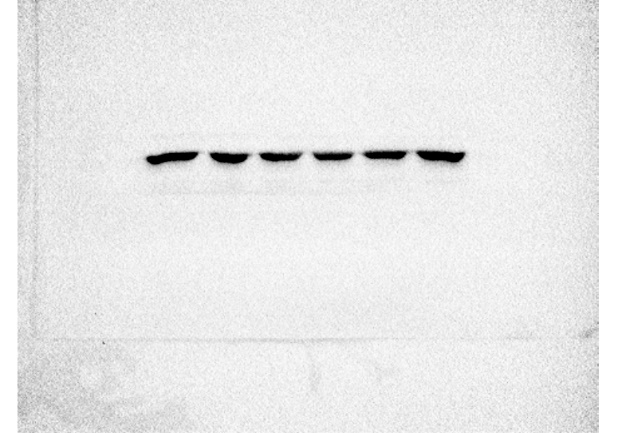


Fig. 4A

p-FAK


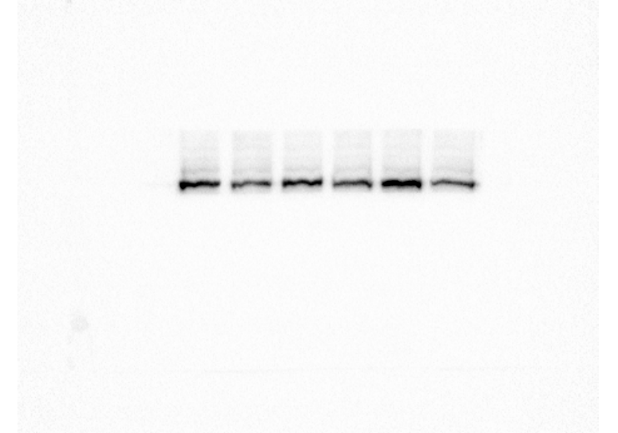

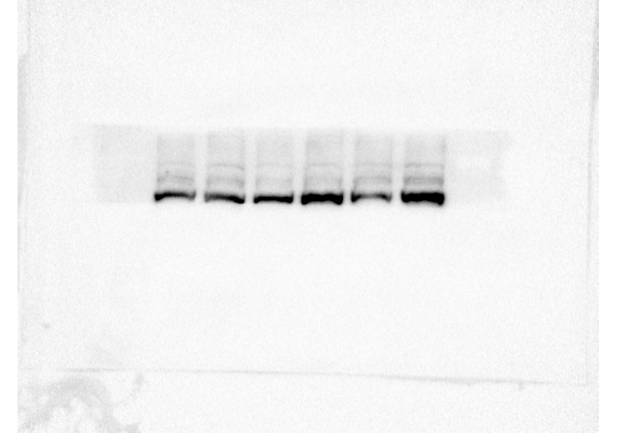


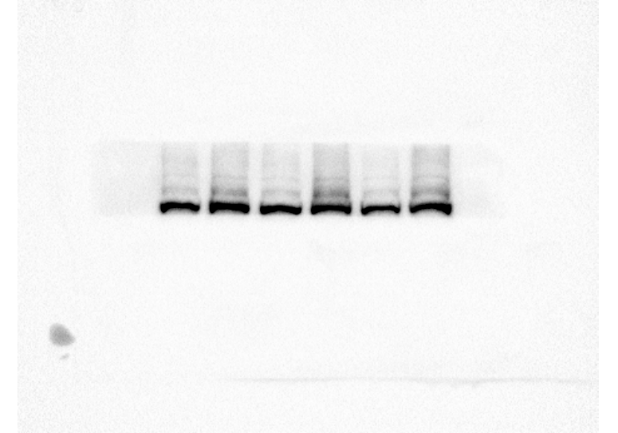


FAK


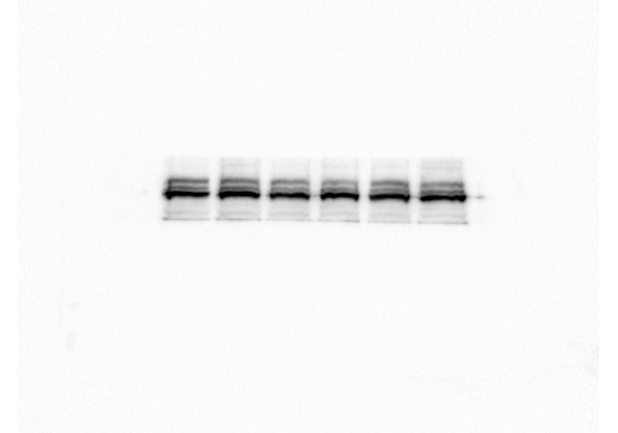

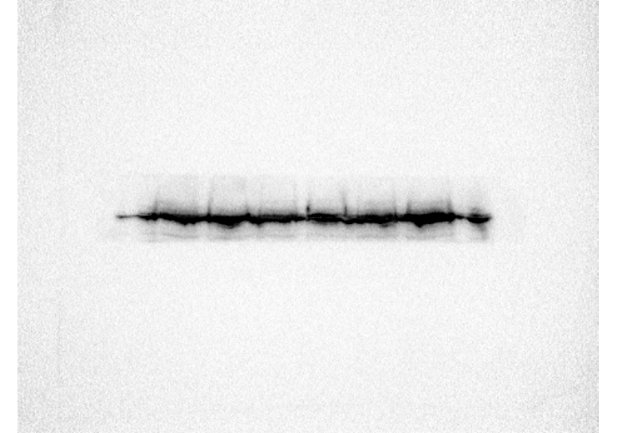


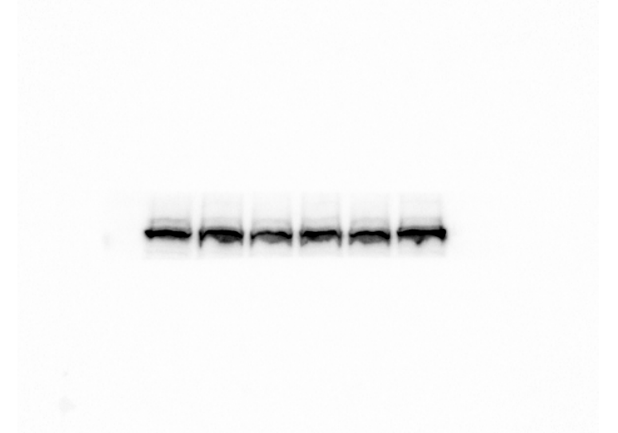


p-Erk 1/2


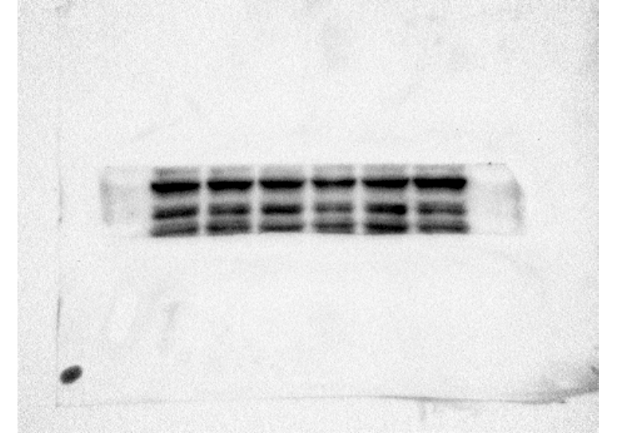

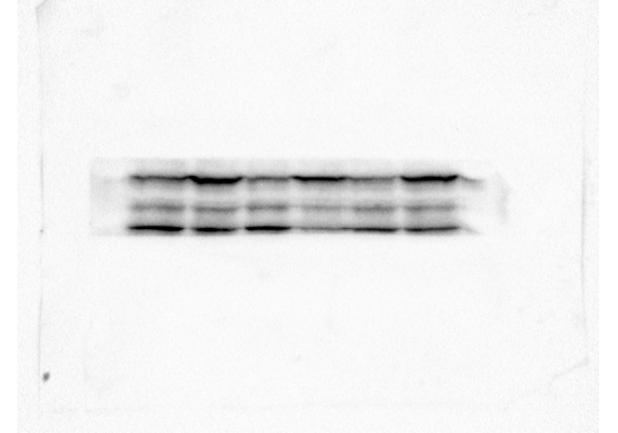


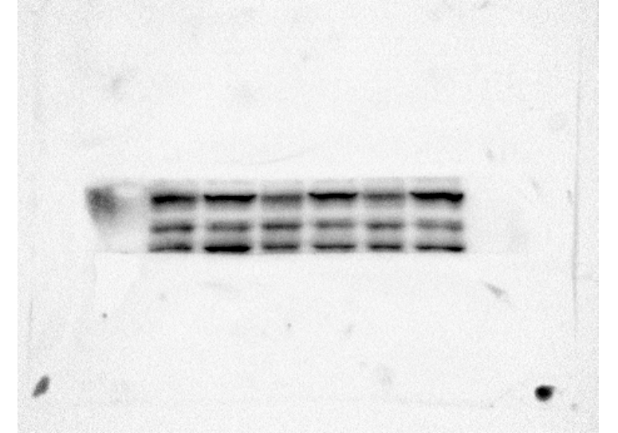


Erk 1/2


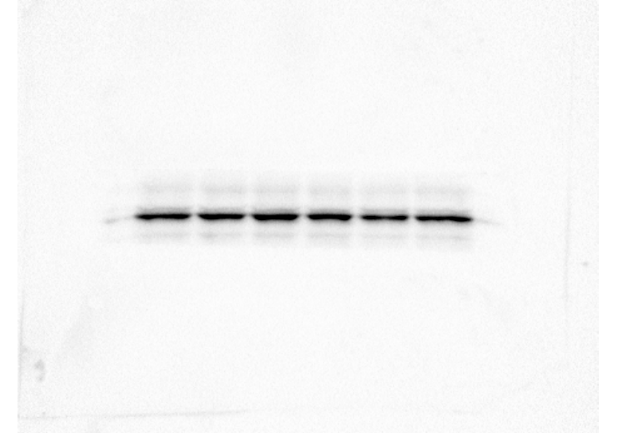

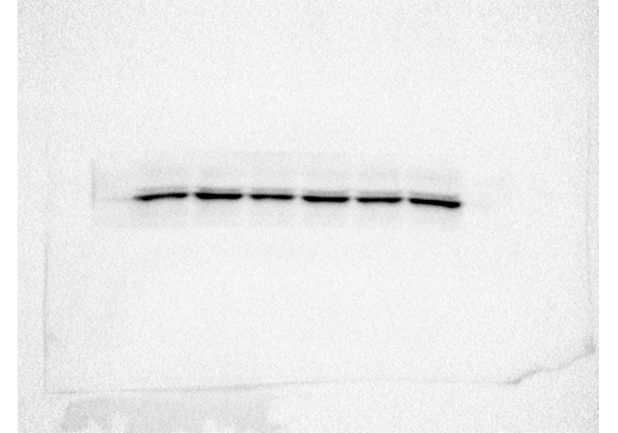


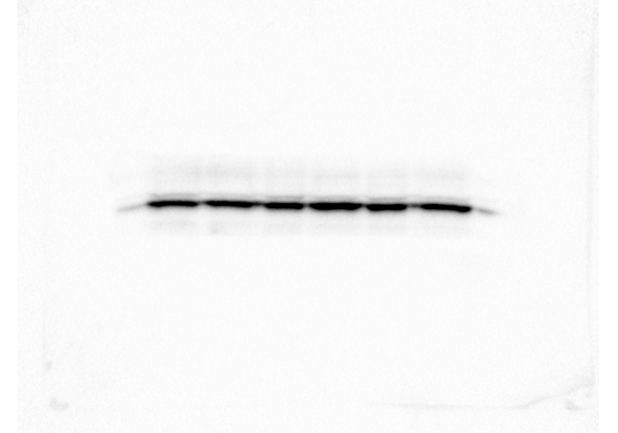


β-actin


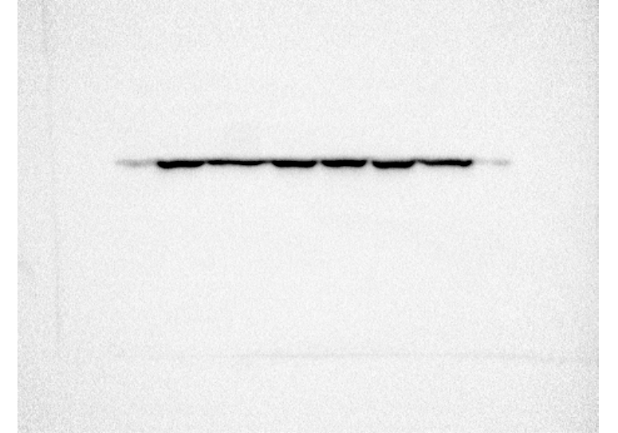

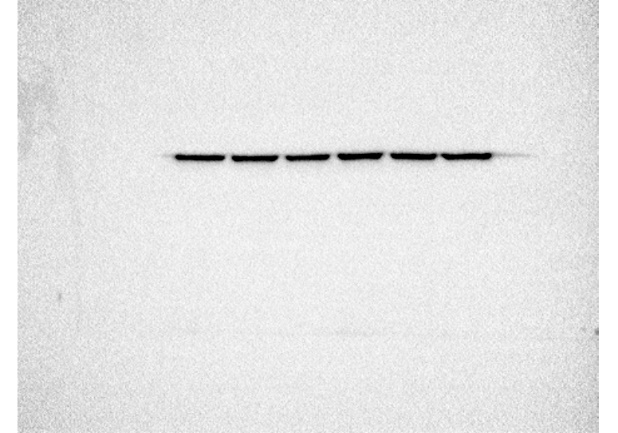


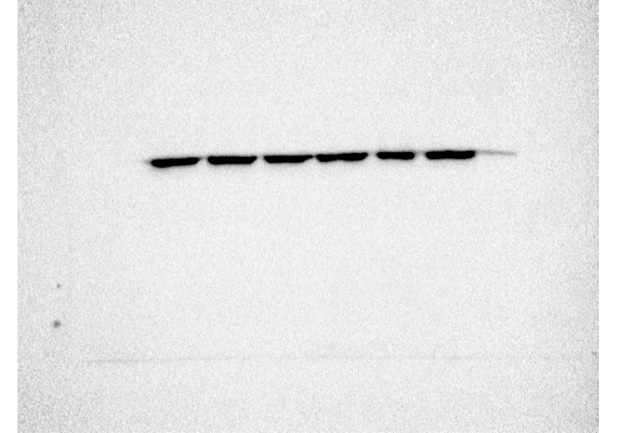


Fig. 3H

p-YAP1


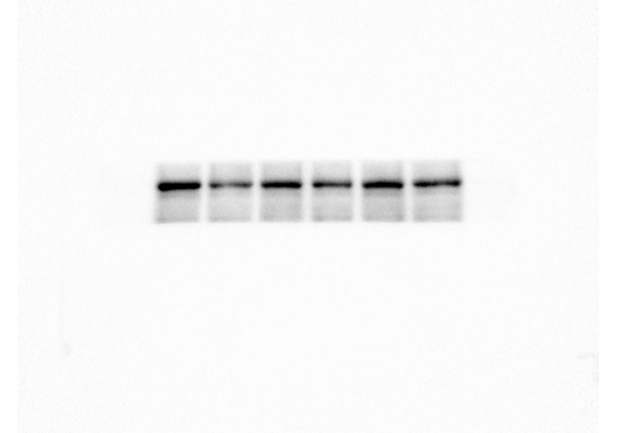

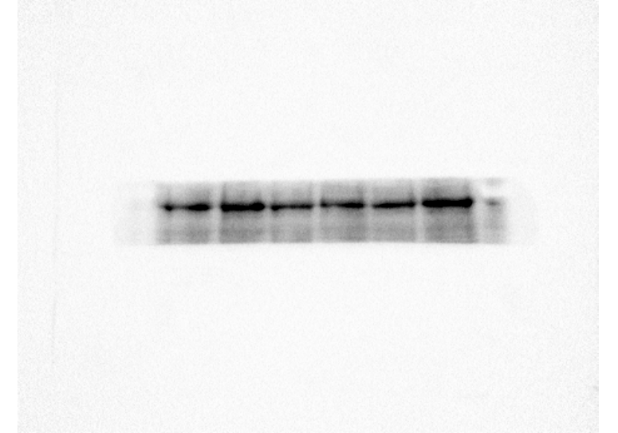


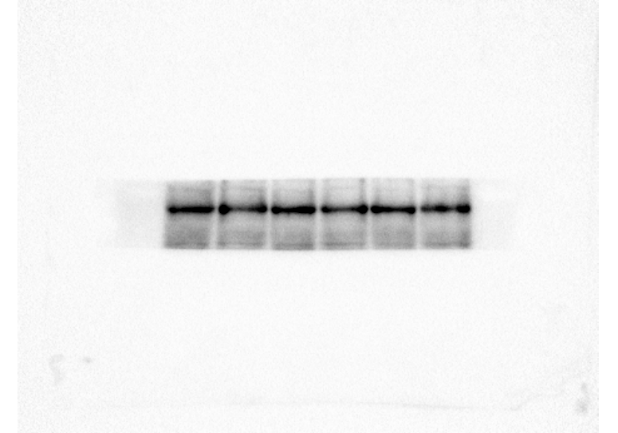


YAP1


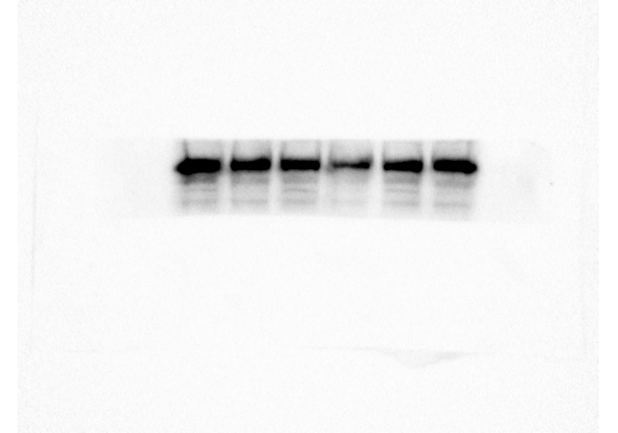

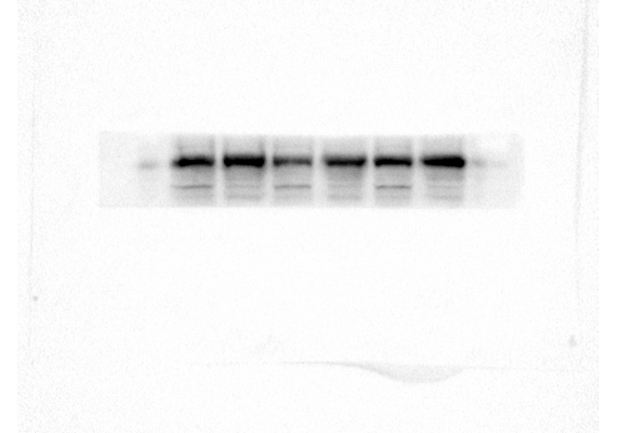


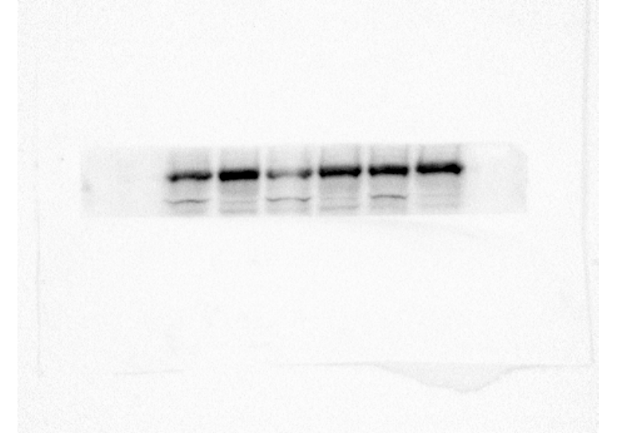


β-actin


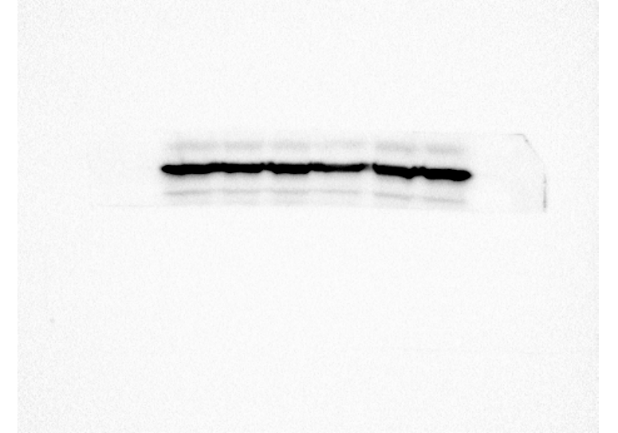

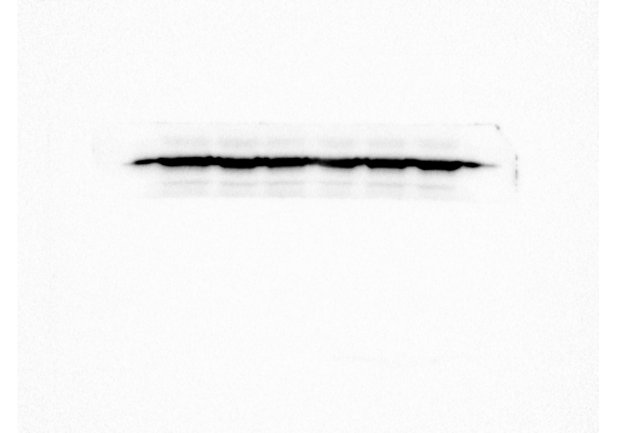


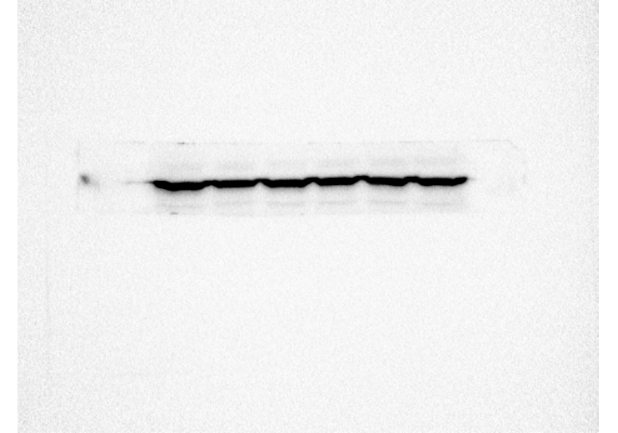


Fig. 4O

FN1


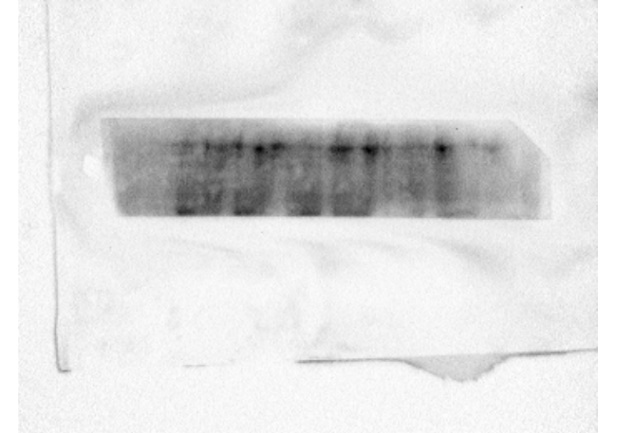

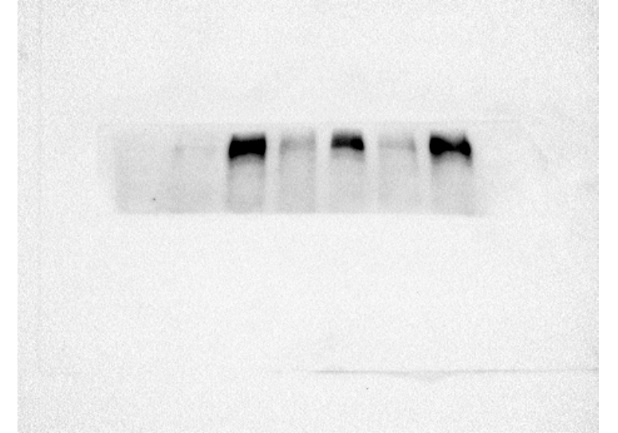


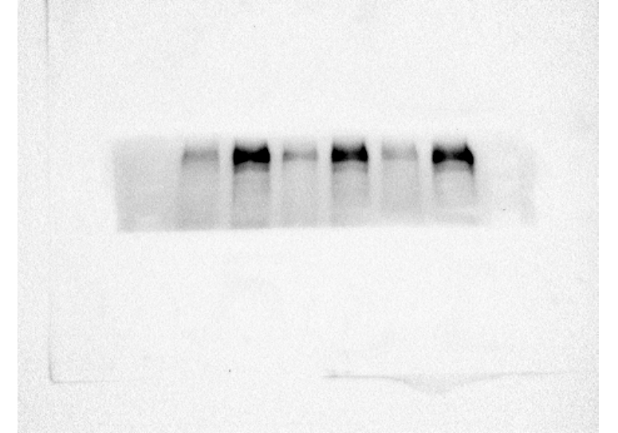


ITGB6


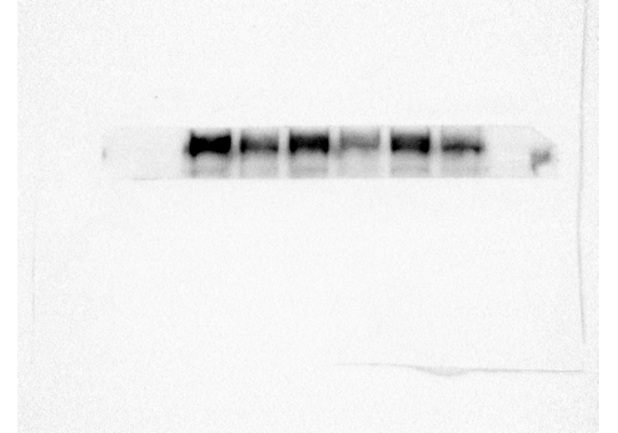

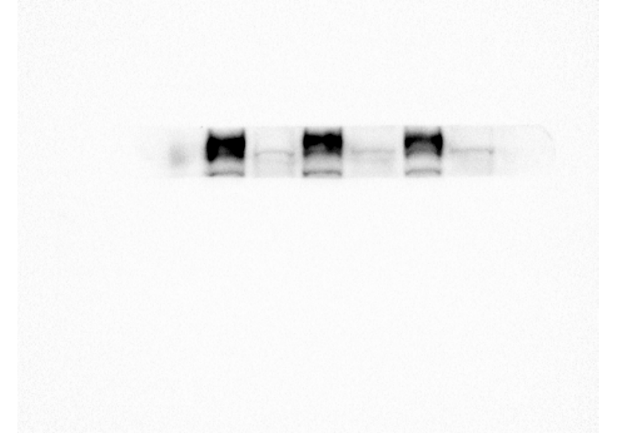


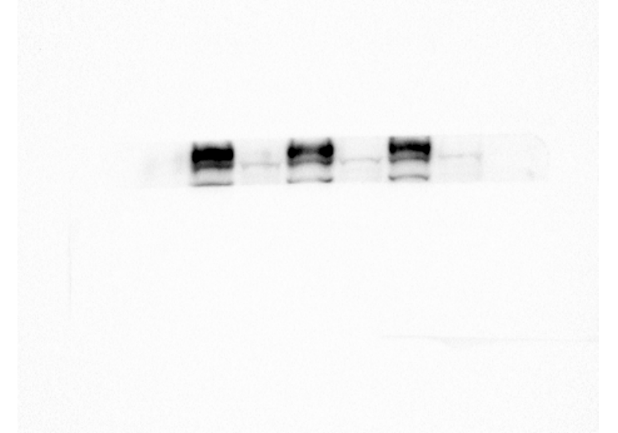


CD44


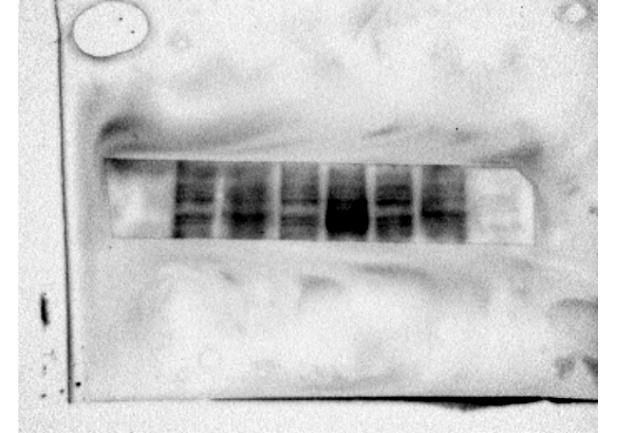

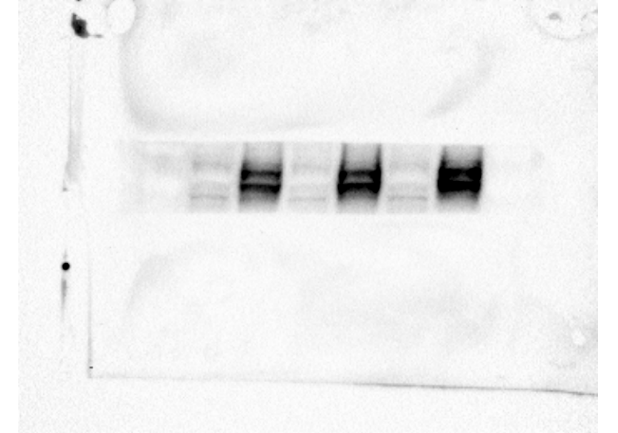


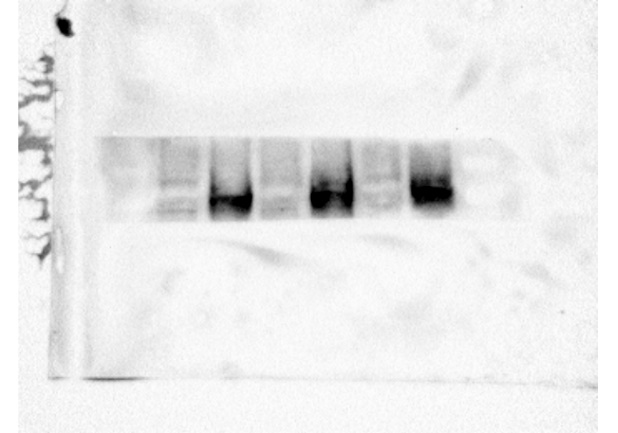


MMP9


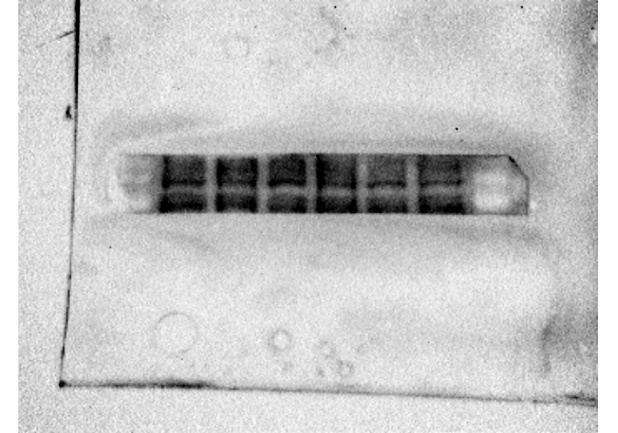

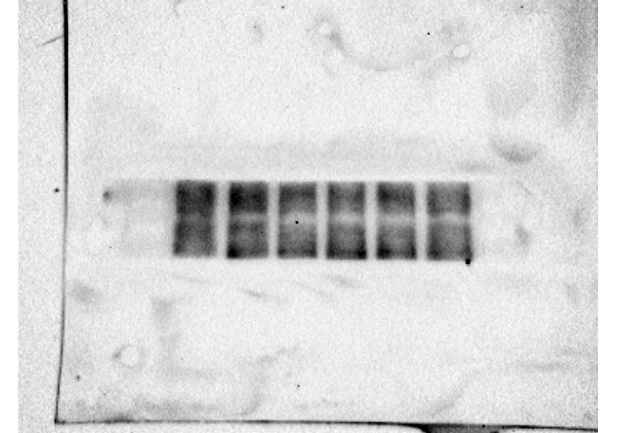


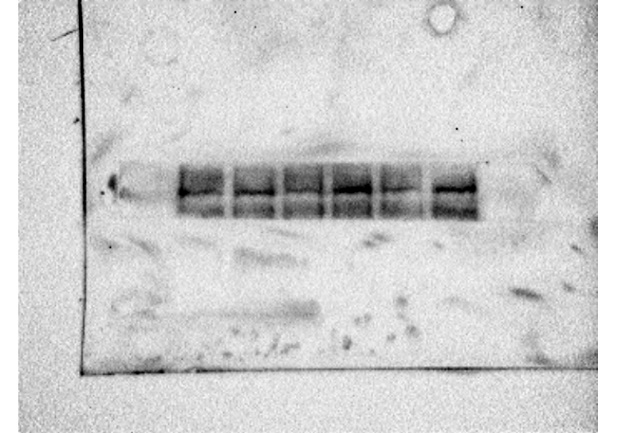


PLOD1


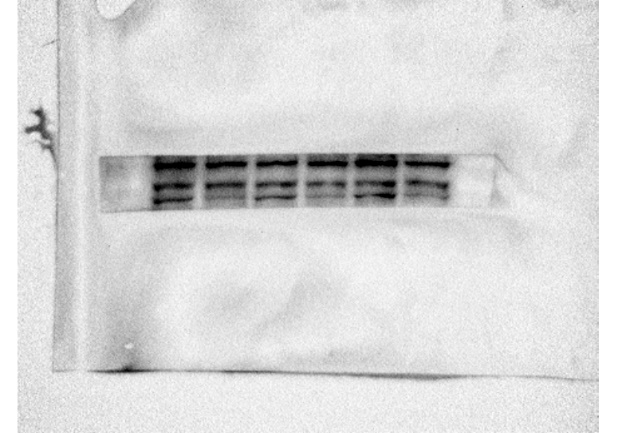

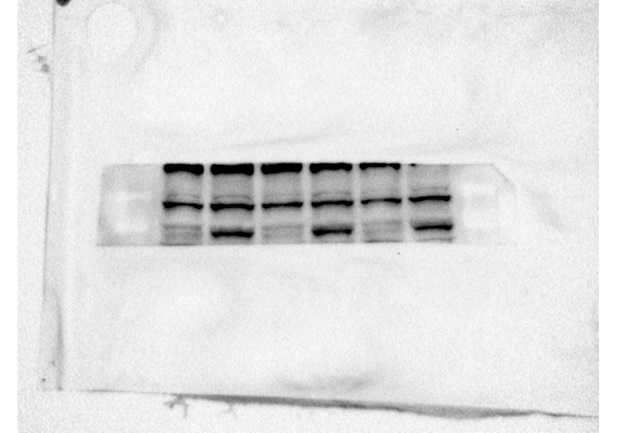


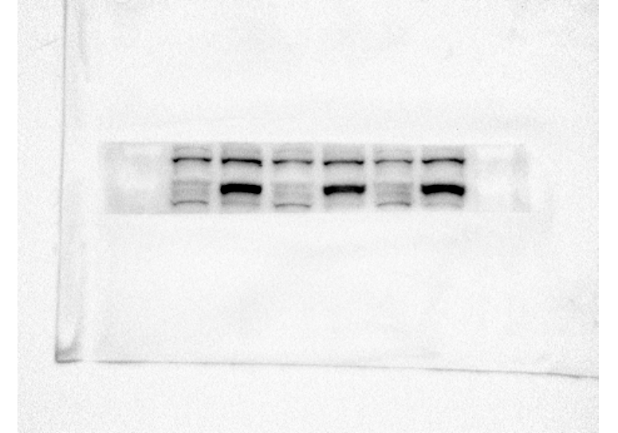


MMP2


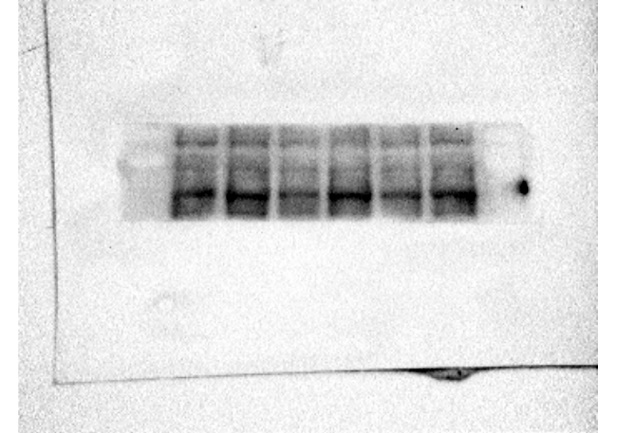

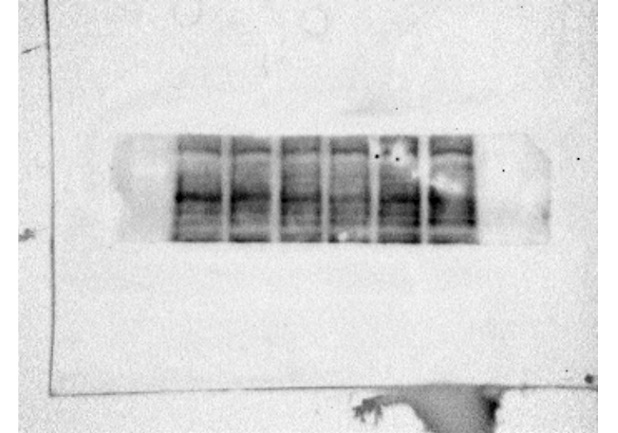


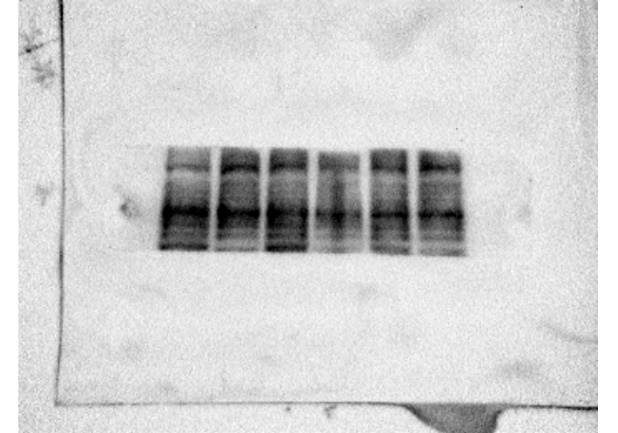


MMP14


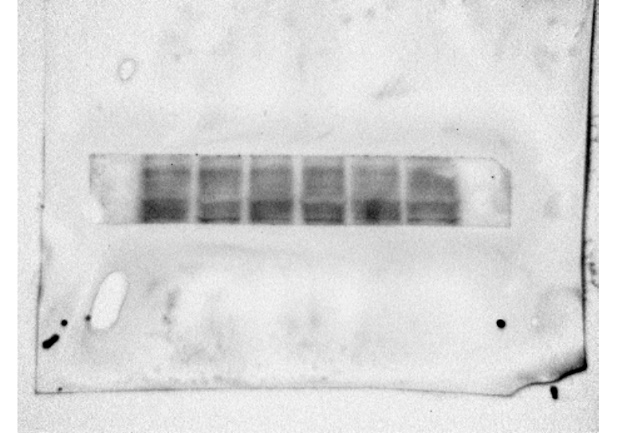

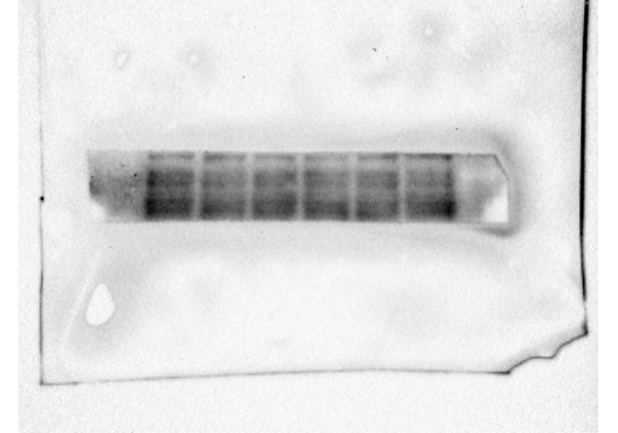


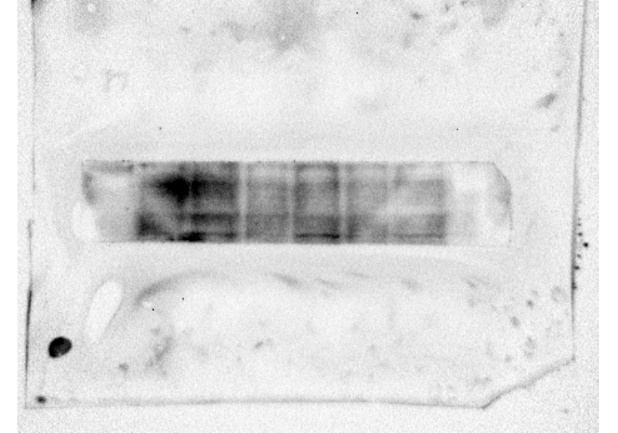


VIMENTIN


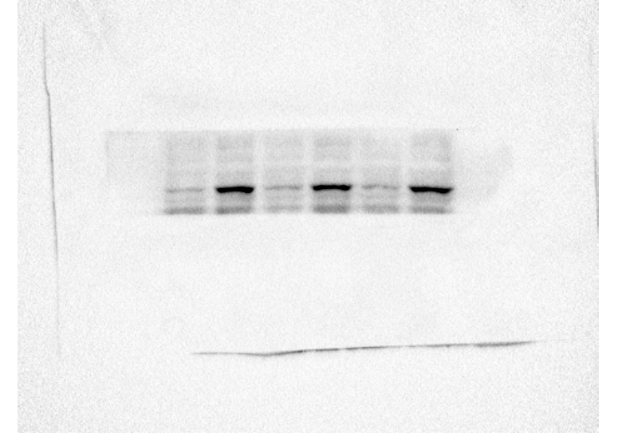

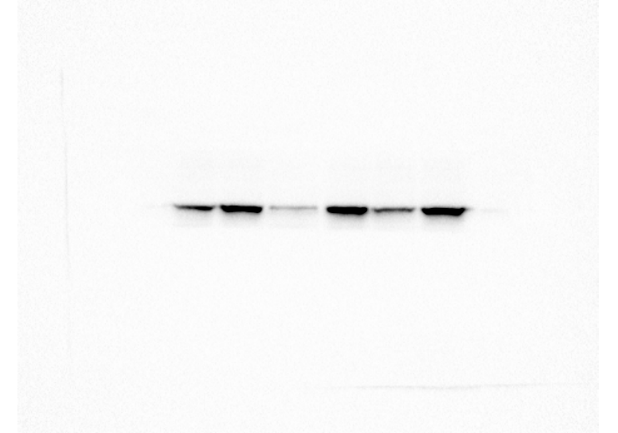


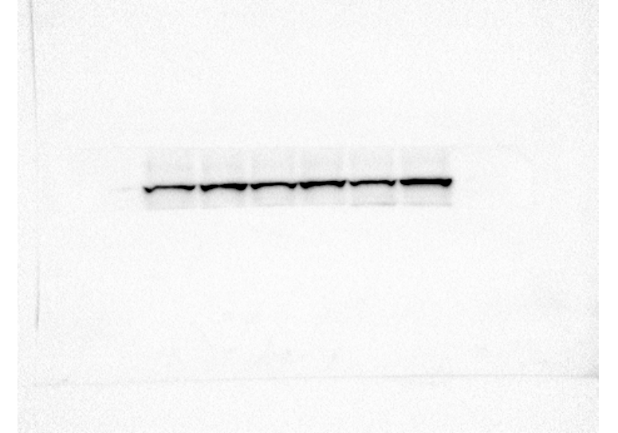


Ep-CAM


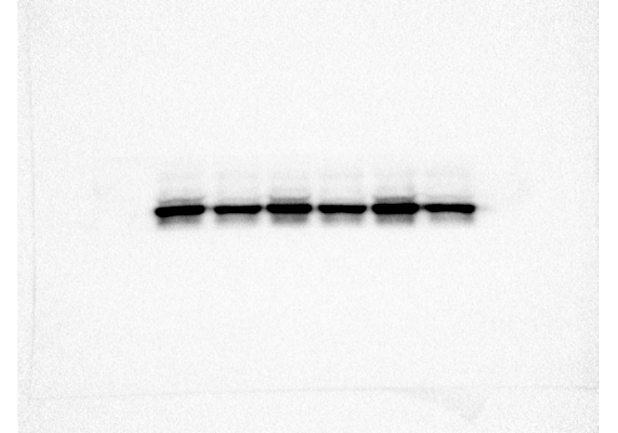

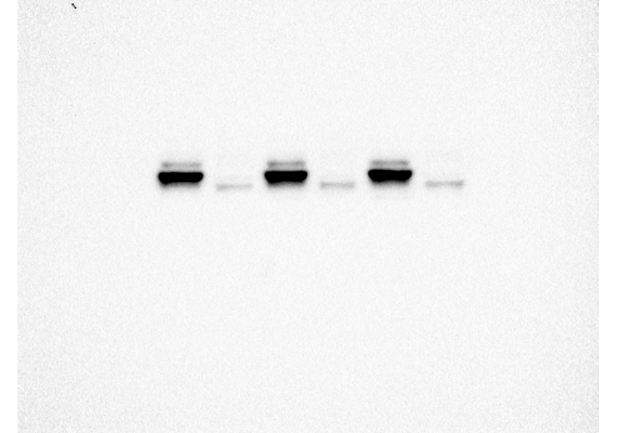


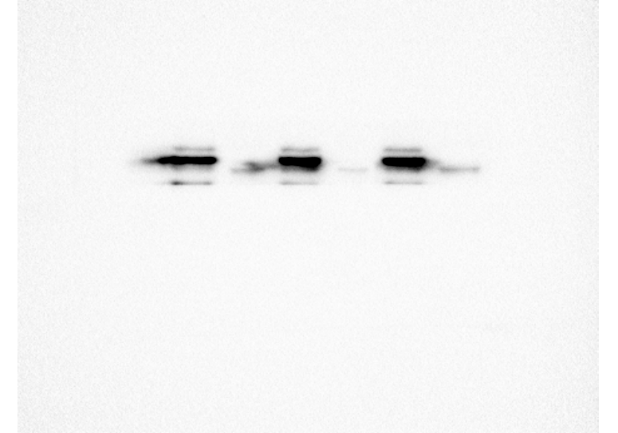


β-actin


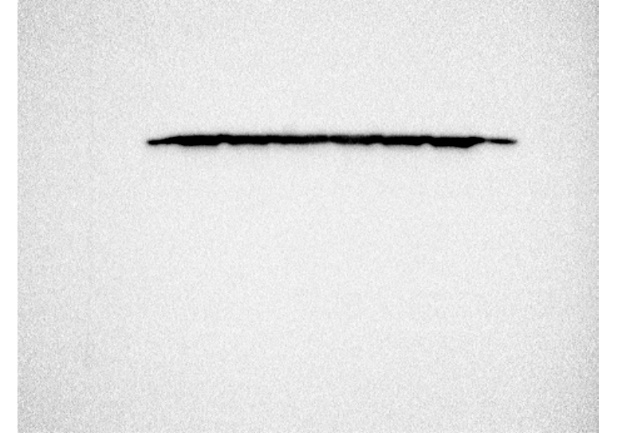


Fig. S3A

FN1

β-actin
